# Brain network biomarkers for diagnosis and clinical stratification in multiple sclerosis: a longitudinal study integrating individualised structural covariance MRI with high-density EEG

**DOI:** 10.1016/j.ebiom.2026.106438

**Published:** 2026-08-19

**Authors:** Ruiping Gao, Xuegang Song, Hanyue Zheng, Yi Men, Jiajian Li, Yingtao Wang, Yuan Qi, Jun Sun, Yinan Zhao, Junwei Hao

**Affiliations:** a Department of Neurology, The First Hospital of Hebei Medical University, Shijiazhuang, Hebei, China; b Department of Neurology, Xuanwu Hospital, Capital Medical University, National Center for Neurological Disorders, Beijing, China; c Hebei Medical University, Shijiazhuang, China; d Beijing Municipal Geriatric Medical Research Center, Beijing, China; e Key Laboratory for Neurodegenerative Diseases of Ministry of Education, Beijing, China; f School of Biomedical Engineering, Guangzhou Medical University, Guangzhou, China

**Keywords:** Multiple sclerosis, Individualised structural covariance network, High-density EEG, Graph theory, Clinical stratification, Machine learning

## Abstract

**Background:**

Multiple sclerosis (MS) is increasingly recognised as a disorder of large-scale brain network reorganisation rather than a disease explained solely by focal demyelinating lesions. However, the relevance of structural network abnormalities to clinical heterogeneity and progression remains unclear.

**Methods:**

We analysed 3T magnetic resonance imaging (MRI) from two independent MS cohorts (total n = 635): Dataset 1 from the Chinese neuroimmunological diseases (NIDBase) cohort (163 MS and 248 healthy controls [HC]) and Dataset 2 from the UK Biobank (117 MS and 107 HC). A subset of Dataset 1 underwent 256-channel resting-state high-density electroencephalography (hd-EEG) (135 MS and 80 HC). We constructed structural covariance networks (iSCNs) from 3D T1-weighted MRI using Kullback–Leibler similarity across 170 Automated Anatomical Labelling atlas 3 (AAL3) regions. Patients were stratified by disability, cognition, and disease activity; a subgroup with no evidence of disease activity (NEDA) with 1-year follow-up MRI (n = 33) was analysed longitudinally. Linear support vector machine classifiers evaluated topological and connectivity features for diagnosis and clinical stratification.

**Findings:**

MS showed reproducible topological and connectivity abnormalities, involving thalamic and subcortical hubs and altered visual-network-related couplings. Disability showed the broadest abnormalities, whereas cognitive impairment and disease activity were associated with more selective changes. Patients meeting NEDA criteria showed subtle longitudinal nodal changes. Connectivity features performed best for MS-vs-HC discrimination, whereas topological features performed better for clinical stratification.

**Interpretation:**

The iSCN approach identified reliable patterns of topological and connectivity impairment across MS and its clinical stratifications, providing insight into MS neuropathology and guiding future diagnostic and therapeutic biomarker development.

**Funding:**

This work was funded by Beijing Research Ward Excellence Program (BRWEP2024W022010104, BRWEP2024W022010109), Beijing Scholar program (No. 106), the Project for Innovation and Development of Beijing Municipal Geriatric Medical Research Center (11000023T000002041657), Dengfeng Talent Program (DFL20220701), 10.13039/501100001809National Natural Science Foundation of China (82571539, 82501612), Xuanwu Hospital Talent Convergence Program–Leading Talents (HZ2021ZCLJ008), the Beijing Hospitals Authority's Ascent Plan (DFL20240801), and Beijing "Huizhi" Talent Program, Cultivation Program–Leading Talents (HZ2025PYLJ003).

**Research in context**

**Evidence before this study**

We searched PubMed, Embase, and Web of Science from database inception to May 25, 2025, without language restrictions. Search terms covered multiple sclerosis, brain networks, structural covariance, individualised or subject-specific networks, multimodal neuroimaging, MRI, EEG, cognition, disability, disease activity, and NEDA. We included human MS studies using network-based neuroimaging approaches (functional connectivity, diffusion-based structural networks, or structural covariance networks) with quantitative network metrics and comparisons across MS phenotypes or clinical outcomes; we excluded non-human studies, non-MS populations, non-network imaging reports, and small descriptive case reports. Prior work has consistently shown widespread network abnormalities in MS, predominantly from group-level functional connectivity or diffusion-derived connectomes. Structural covariance studies in MS are fewer, and most have been group-level, with limited individualised modelling and scarce multimodal validation. Overall evidence is constrained by cross-sectional designs, heterogeneous pipelines and parcellations, modest sample sizes, and limited external validation, which reduces confidence in patient-level stratification. Formal meta-analysis was not undertaken because of methodological and outcome heterogeneity.

**Added value of this study**

We evaluated the clinical relevance of individualised structural covariance networks in MS using subject-specific modelling in two independent MRI cohorts, with external validation, robustness analyses using an alternative atlas and similarity metric, and cross-modal support from 256-channel resting-state EEG in a subset. We show that iSCN measures distinguish MS from healthy controls and enable clinically meaningful stratification by disability severity, processing-speed impairment, and disease activity. Machine-learning analyses indicate that topology-derived features are particularly informative for clinical stratification, whereas connectivity features are more informative for diagnosis-related differences. Longitudinal analyses in clinically stable patients meeting NEDA criteria showed subtle nodal changes, suggesting that the iSCN approach may detect subclinical network evolution not captured by routine clinical or radiological assessments.

**Implications of all the available evidence**

Together with existing evidence, our findings support MS as a disorder of system-level network reorganisation rather than isolated focal pathology. Individualised network profiling may complement conventional MRI by improving characterisation of heterogeneity across disability, cognition, and disease activity, and by providing quantitative markers sensitive to subclinical change in clinically stable patients. With further longitudinal validation and standardised pipelines, iSCN-based measures could refine patient stratification and support mechanistic studies and trial designs addressing subclinical progression in MS.

## Introduction

Multiple sclerosis (MS) is a chronic immune-mediated disease characterised by demyelination and axonal loss within the central nervous system (CNS),^1^ involving both white and grey matter structures.^2^ Increasing evidence suggests that MS involves widespread disruption of large-scale brain networks and cannot be fully explained by focal lesions alone.^3^ Such distributed abnormalities alter structural and functional network organisation and may contribute to heterogeneous impairment across motor, cognitive, and sensory domains.^4^, ^5^, ^6^

Magnetic resonance imaging (MRI) remains central to MS research and clinical assessment, yet conventional region-based measures provide limited insight into the brain's systems-level organisation. Network-based imaging studies have extended MS research to connectivity, but most have focused on white matter structural connectivity or resting-state functional connectivity,^5^^,^^7^ while grey matter structural networks, particularly individualised structural covariance networks (iSCNs), remain underexplored in MS.^8^ This gap is important because individualised grey matter networks may capture subtle network alterations associated with clinical heterogeneity.^9^ Furthermore, many previous studies have relied on modest single-centre datasets and lacked external replication or multimodal support,^10^^,^^11^ limiting reproducibility and clinical translation.

iSCNs derived from subject-specific morphology provide higher sensitivity to heterogeneity, subtle regional alterations and treatment responses.^12^^,^^13^ High-density electroencephalography (hd-EEG) provides a complementary approach for functional network analysis.^14^^,^^15^ Source-level hd-EEG enables the assessment of large-scale functional networks with high temporal resolution and may help interpret MRI-derived structural abnormalities in relation to functional network organisation.^16^^,^^17^

To address this gap, we investigated brain network organisation in two independent case–control cohorts: 163 patients with MS and 248 healthy controls (HC) from Xuanwu Hospital (Chinese Neuroimmunological Diseases [NIDBase] cohort; Dataset 1, discovery cohort), and 117 patients with MS and 107 HC from the UK Biobank (Dataset 2, external validation cohort).^18^ We analysed network topology and connectivity patterns, evaluated their discriminative performance for MS diagnosis and predefined clinical stratification using a support vector machine (SVM), and examined their associations with clinical measures, including the Expanded Disability Status Scale (EDSS), Symbol Digit Modalities Test (SDMT), and disease activity status defined by No Evidence of Disease Activity-3 (NEDA-3). In the NEDA-3 subgroup, longitudinal comparison between baseline and 1-year follow-up MRI was used to characterise subtle network reorganisation and early structural progression.^19^ We chose SVM because of its suitability for high-dimensional neuroimaging data and its widespread use in neuroimaging classification studies.^20^ We further sought cross-modal corroboration of the MRI findings using hd-EEG functional networks,^21^ examining both topological properties and connectivity patterns. Robustness of the iSCN findings was additionally assessed using the Brainnetome atlas and Jensen–Shannon divergence (JSD)-based sensitivity analyses.^22^^,^^23^ Overall, this study aimed to clarify the diagnostic, stratification, and longitudinal relevance of individualised structural network abnormalities in MS.

## Methods

### Participants and study design

**Fig. 1:**
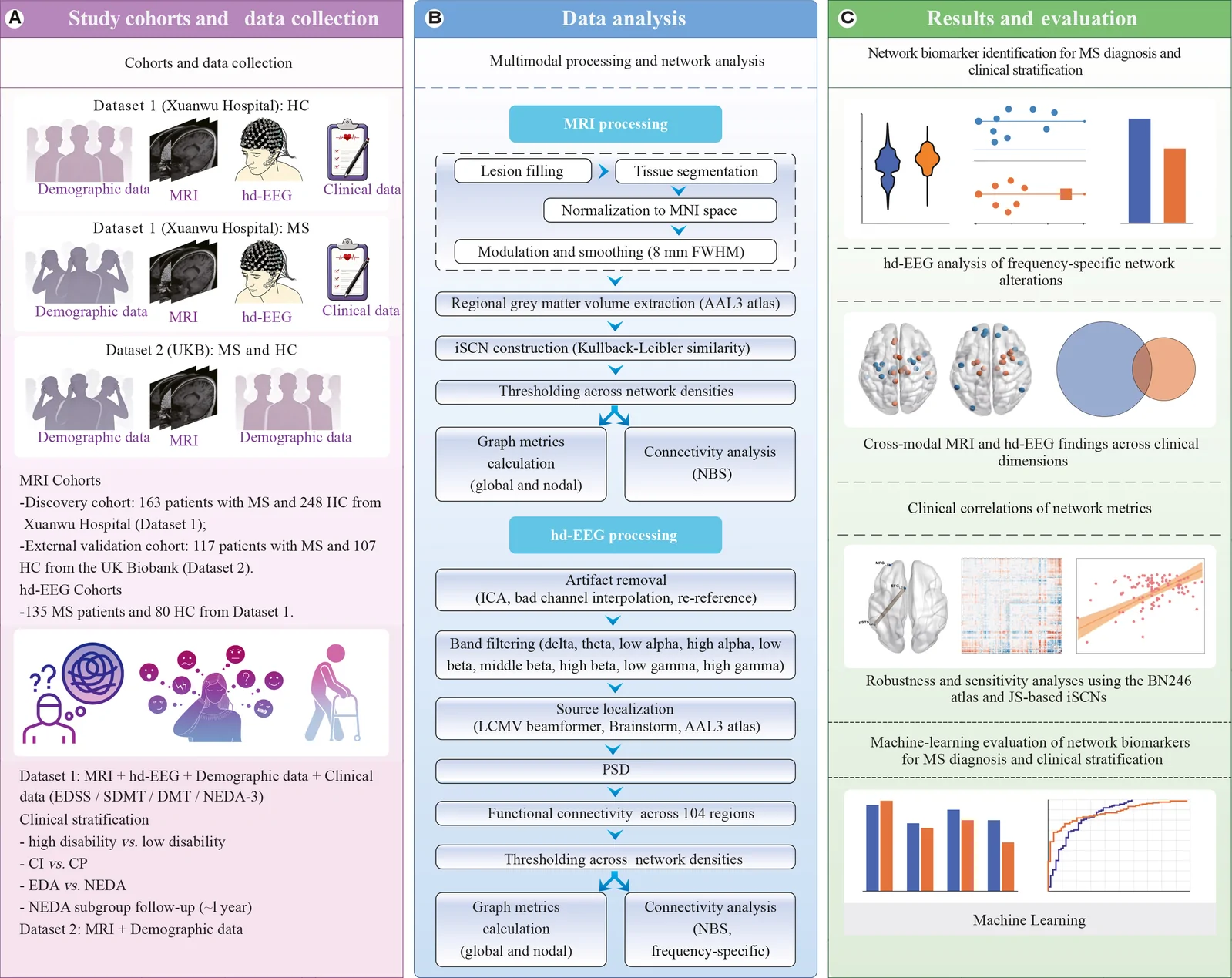
**Study design and analytical workflow. (A)** Study cohorts and data collection. Dataset 1 from Xuanwu Hospital served as the discovery cohort and included 163 patients with MS and 248 HC for MRI analyses. A multimodal subset of Dataset 1 further included 135 patients with MS and 80 HC with hd-EEG data. Prespecified clinical stratification in Dataset 1 included disability, cognition, and disease activity, and a subgroup of patients meeting NEDA criteria who had follow-up MRI at approximately one year was included for longitudinal analysis. Dataset 2 from UKB served as an independent external validation cohort and included 117 patients with MS and 107 HC with structural MRI and demographic data. **(B)** Data analysis. Structural MRI underwent lesion filling, tissue segmentation, normalisation to MNI space, modulation, and smoothing, followed by regional grey matter volume extraction using AAL3. iSCNs were constructed using Kullback–Leibler similarity, thresholded across a range of network densities, and analysed using graph-theoretical metrics and NBS. hd-EEG data underwent artefact removal, source localisation, and Pearson-correlation-based functional connectivity estimation of regional power spectral density across 104 regions, followed by thresholding across a range of network densities and graph-theoretical and connectivity analyses. **(C)** Results and evaluation. The analytical framework enabled identification of network abnormalities related to MS diagnosis and clinical stratification, cross-modal comparison of MRI and hd-EEG findings, clinical correlation analyses, robustness and sensitivity analyses using BN246 and JS-based iSCNs, and machine-learning evaluation of topology- and connectivity-based biomarkers. **Abbreviations:** AAL3, Automated Anatomical Labelling atlas 3; BN246, Brainnetome atlas with 246 regions; HC, healthy controls; hd-EEG, high-density electroencephalography; iSCN, individualised structural covariance network; JS, Jensen–Shannon; MNI, Montreal Neurological Institute; MS, multiple sclerosis; NBS, network-based statistics; NEDA, no evidence of disease activity; UKB, UK Biobank.

Dataset 1 comprised 163 patients with MS and 248 HC recruited from Xuanwu Hospital, Capital Medical University. Recruitment and data collection for Dataset 1 were conducted from February 2024 to March 2026. MS diagnosis followed the 2017 McDonald criteria.^24^ All participants underwent standardised 3D T1-weighted and 3D T2-weighted fluid-attenuated inversion recovery (T2-FLAIR) MRI using identical protocols; HC were recruited during the same period. Exclusion criteria included age <18 or >65 years and other neurological or psychiatric disorders. Dataset 2 was derived from the UK Biobank (Application ID: 917687177) and included 117 patients with MS and 107 HC with available 3D T1-weighted MRI and demographic data. A subset of Dataset 1 participants (135 MS and 80 HC) also underwent 256-channel resting-state hd-EEG, forming the multimodal EEG cohort (Fig. 1). Full inclusion and exclusion criteria are provided in Supplementary Methods S1.

### Clinical assessment

In Dataset 1, disability, cognitive processing speed, and disease activity were assessed using the EDSS, SDMT, and NEDA-3 criteria, respectively.^25^ Age, sex, education, body mass index (BMI), total intracranial volume (TIV), disease duration, and disease-modifying therapy (DMT) status were recorded. Sex was self-reported by participants at enrolment in Dataset 1 and was obtained from UK Biobank records in Dataset 2. Sex was recorded as female or male; gender identity was not collected. Sex was considered during study design through sex matching of the healthy control group in Dataset 1 and was included as a prespecified covariate in the relevant analyses. Cognitive status was stratified using age-, sex-, and education-adjusted normative SDMT z-scores derived from the HC group. In Dataset 2, only age, sex, BMI, and TIV were available as covariates; EDSS and SDMT data were unavailable. Because Dataset 2 was analysed separately as an external validation cohort, no cross-cohort harmonisation for site effects was performed.

### MRI acquisition and processing

All MRI scans were acquired on 3T scanners using cohort-specific protocols. Dataset 1 used a GE Discovery MR750 scanner (8-channel head coil): 3D T1-weighted BRAVO (TR = 7.8 ms, TE = 2.9 ms, TI = 743 ms, 1 × 1 × 1 mm^3^) and 3D T2-FLAIR CUBE (TR = 6300 ms, TE = 105 ms, TI = 1775 ms, 1.0 × 1.0 × 1.4 mm^3^). Dataset 2 used a 3D T1-weighted MPRAGE sequence (TR = 2000 ms, TE = 2.01 ms, TI = 880 ms, 1 × 1 × 1 mm^3^). Preprocessing was performed using the SPM toolbox (http://rfmri.org/SPM).^26^ In Dataset 1, T2-FLAIR was co-registered to T1 and used for lesion segmentation/filling with conventional SPM12-based LST rather than LST-AI, followed by visual quality control before CAT12 processing.^27^ In Dataset 2, white matter lesions were manually delineated using ITK-SNAP, lesion-filled T1 images were generated, and the filled T1 images then underwent CAT12-based segmentation. Images were segmented into grey matter (GM), white matter and cerebrospinal fluid, normalised to Montreal Neurological Institute (MNI) space via diffeomorphic anatomical registration through exponentiated Lie algebra (DARTEL), modulated, and smoothed with an 8 mm Gaussian kernel. Regional grey matter volume (GMV) maps were extracted for subsequent network construction. The full MRI processing pipeline is provided in Supplementary Method S2.

### iSCN construction and graph-theoretical analysis

Each participant's iSCN was constructed using 170 cortical/subcortical regions from the AAL3 atlas. GM volume probability distributions were estimated via kernel density estimation (KDE); interregional similarity was quantified by Kullback–Leibler divergence (KLD) and converted to Kullback–Leibler similarity (KLS = e−KLD), forming 170 × 170 similarity matrices. JSD sensitivity analysis was also performed (Supplementary Methods S3).^28^ The calculation of KLD/JSD included three steps: (1) extracting voxel-wise grey matter volume values within each brain region, (2) estimating the probability density function (PDF) of each brain region using a normal kernel function with a fixed number of sampling points (MATLAB function, ksdensity), and (3) calculating KLD/JSD between every pair of regional PDFs. This procedure represents each ROI as a normalised GMV distribution and reduces dependence on the absolute number of voxels in each ROI; therefore, KLD/KLS quantifies interregional similarity in GMV distributional shape rather than raw ROI volume.

KLD was calculated as follows:

$$KLD\left(P,Q\right)=\sum_{i=1}^{n}\left(P\left(i\right)\log\frac{P\left(i\right)}{Q\left(i\right)}+Q\left(i\right)\log\frac{Q\left(i\right)}{P\left(i\right)}\right)$$

JSD was first calculated and then transformed into JS similarity as follows:

$$JSDs\left(P,Q\right)=1-\sqrt{JSD\left(P\|Q\right)}$$

$$JSD\left(P\|Q\right)=\frac{1}{2}\sum_{i=1}^{n}\left(P\left(i\right)\log\frac{P\left(i\right)}{\frac{1}{2}\left(P\left(i\right)+Q\left(i\right)\right)}+Q\left(i\right)\log\frac{Q\left(i\right)}{\frac{1}{2}\left(P\left(i\right)+Q\left(i\right)\right)}\right)$$

For graph-theoretical analyses, each weighted similarity matrix was thresholded and binarised across a predefined density range using a sparsity-based approach that retained the strongest K% of edges. Thresholds ranged from 10% to 40% (1% increments), selected to preserve network connectedness and small-world organisation (σ > 1.1). To reduce dependence on any single threshold, global and nodal graph metrics were summarised as area under the curve (AUC) across thresholds for subsequent statistical analyses (Supplementary Methods S3). Global metrics included the normalised clustering coefficient (γ), normalised characteristic path length (λ), small-worldness (σ = γ/λ), clustering coefficient (Cp), characteristic path length (Lp), global efficiency (Eg), local efficiency (Eloc), modularity (Q), and assortativity (r), reflecting network segregation, integration, small-world organisation, modular structure, and resilience. Nodal metrics included nodal degree (Dc), nodal betweenness (Bc), nodal efficiency (Ne), characterising the relative importance and information flow capacity of individual brain regions.^29^ Formal definitions of all metrics are provided in Supplementary Method S4.

### Analysis of connectivity patterns of iSCN

Network-based statistics (NBS) was used to test between-group differences in structural covariance connectivity.^30^ An initial edge-wise threshold of two-tailed *p* < 0.001 was applied, and statistical significance was assessed at the component level using 10,000 permutations, with family-wise error-corrected *p* (*p*_FWE_) < 0.05 considered significant. Significant NBS-derived subnetworks were then mapped onto the seven canonical intrinsic functional networks described by Yeo et al.^31^, ^32^, ^33^, ^34^: the default mode network (DMN), ventral attention network (VAN), visual network (VN), dorsal attention network (DAN), somatomotor network (SMN), frontoparietal network (FPN), and limbic network (LN).^35^ At the network-pair level, altered edges were summarised for each within-network and between-network pair. Relative connection weight was defined as the number of altered edges divided by the maximum possible number of edges for that network pair. Statistical significance of network-pair-level over-representation was assessed as described in Supplementary Method S5, and FDR-corrected *p* values are reported as *q* values.

### hd-EEG acquisition and analysis

hd-EEG was recorded with a 256-channel system (sampling rate 1000 Hz, bandpass 0.1–104 Hz) in shielded rooms during alternating eyes-open and eyes-closed periods for a total of 30 min, as detailed in the Supplementary Methods. Resting-state analyses were performed on eyes-closed data. Source-space signals were parcellated using the AAL3 atlas, of which 104 cortical and subcortical regions could be reliably reconstructed after source localisation (Supplementary Table S14). Preprocessing in EEGLAB included band-pass filtering, spherical spline interpolation, independent component analysis (ICA)-based artefact removal, and linearly constrained minimum variance (LCMV) beamformer source localisation. EEG functional networks were constructed for nine frequency bands using Pearson correlation of regional power spectral density across epochs. Graph-theoretical and NBS analyses followed the same framework as the iSCN analyses. Significant NBS-derived altered edges were further summarised at the functional-network level across the same seven canonical networks, with relative connection weight and FDR-corrected *q* values calculated as described for the MRI analyses. Full details are provided in Supplementary Methods S7.

### Subgroup definition

Patients in Dataset 1 were stratified by disability severity using the EDSS as low disability (EDSS ≤3.5) and high disability (EDSS ≥4.0).^36^ Cognitive status was defined using age-, sex-, and education-adjusted SDMT z-scores, with z-scores ≤ −1.5 indicating cognitively impaired (CI) and z-scores > −1.5 indicating cognitively preserved (CP).^32^ Disease activity status was determined according to the established NEDA-3 criteria, defined as the absence of (1) clinical relapse, (2) disability progression confirmed by an increase of ≥1 point in EDSS(≥0.5 if baseline EDSS >5.5), and (3) new or enlarging T2 lesions or gadolinium-enhancing lesions on MRI during the one-year follow-up period.^25^ Patients fulfilling all three criteria were classified as NEDA, whereas those with any evidence of disease activity were classified as EDA. A subset of patients meeting NEDA criteria who had available follow-up MRI was included in the longitudinal analyses. The same analytical framework and significance threshold were applied to both MRI- and EEG-derived network measures.

### Individual-level classification performance of SVM tasks

In addition to group-level analyses, we assessed the classification performance of topology-based and connectivity-based features derived from MRI-based iSCNs and EEG networks. In Dataset 1, four MRI-based classification tasks were evaluated: MS vs HC (163 vs 248), high vs low disability (33 vs 130), CI vs CP (44 vs 119), and EDA vs NEDA (69 vs 94). The MRI-based MS-vs-HC task was further examined in the independent Dataset 2 cohort (117 vs 107). In the EEG cohort, an additional MS-vs-HC classification task (135 vs 80) was performed, with features modelled separately for each of the nine frequency bands. Separate linear SVM models were trained for topology-based and connectivity-based features in each task (Supplementary Methods S6). Stratified 10-fold cross-validation was used, and feature scaling, feature selection, and hyperparameter tuning were performed within the training data of each fold to avoid information leakage. Model performance was evaluated using accuracy, sensitivity, specificity, balanced accuracy, and the area under the receiver operating characteristic curve (AUC); 95% confidence intervals and permutation-based comparisons with chance-level performance are reported in the Supplementary Materials. To summarise feature importance, we calculated the mean absolute weight of each feature across cross-validation folds for each model. The ten highest-contributing regions were identified and mapped. Because feature contributions in brain-based predictive models are often long-tailed, focussing on the top ten regions facilitated interpretation while reducing the influence of weakly contributing regions.^37^ For topology-based models, regional contribution maps were derived from nodal features by averaging the absolute weights of the three nodal centrality measures for each region; global topological features were not included in the regional mapping. For connectivity-based models, regional contributions were calculated by averaging the absolute weights of all connections associated with each region.

### Reproducibility and sensitivity analyses

To assess robustness, we performed additional analyses using alternative methodological choices. First, to examine whether results were influenced by node definition, we repeated the iSCN analyses using the Brainnetome atlas (BN246; 246 cortical and subcortical regions). Second, to evaluate whether findings depended on the interregional similarity metric, we reconstructed individual networks using JS divergence and repeated the main topological and connectivity analyses under the same statistical framework. Third, to test the effect of threshold selection, we repeated the graph-theoretical analyses using two additional density ranges, K = 0.20–0.50 and K = 0.30–0.60, defined independently of the small-worldness criterion.

### Ethics

The study protocol was approved by the Ethics Committee of Xuanwu Hospital, Capital Medical University (Approval No: 2021-017-002). Written informed consent was obtained from all Dataset 1 participants at enrolment. The study was conducted in accordance with the Declaration of Helsinki (2024 revision).

### Statistics

#### Sample-size determination, randomisation, and blinding

No formal a priori sample-size calculation was performed. The sample size was determined by the number of eligible participants with complete imaging and required clinical or demographic data available in the prespecified cohorts. Randomisation and allocation concealment were not applicable because this was an observational study. No formal blinding was used during participant recruitment, data collection, image processing, or statistical analysis.

#### Group comparisons and multiple-comparison correction

Normality and variance homogeneity were assessed using the Shapiro–Wilk and Levene's tests, respectively. Continuous variables were compared using independent-samples t tests or Mann–Whitney U tests, as appropriate, and categorical variables using χ^2^ tests. Data are presented as mean ± standard deviation (SD), median (interquartile range [IQR]), or n (%). All tests were two-sided. In the longitudinal NEDA subgroup, baseline and follow-up nodal metrics were compared using paired t tests. Multiple comparisons were controlled using false discovery rate (FDR) correction, and FDR-corrected *p* values are reported as q values. Effect sizes for significant findings are reported as Cohen's d.

#### Covariate adjustment

For both MRI- and EEG-derived topological and connectivity measures, covariates were prespecified according to the comparison of interest. In MRI-based case–control analyses (MS vs HC), models were adjusted for age, sex, BMI, and TIV; in EEG-based case–control analyses, models were adjusted for age, sex, and BMI. Within the MS cohort, disability and disease-activity subgroup analyses were adjusted for age and sex, with additional adjustment for TIV in MRI-based models. Cognitive subgroup analyses were adjusted for TIV, disease duration, and DMT status in MRI, and for disease duration and DMT status in EEG. Age, sex, and education were not re-entered in the cognitive subgroup models because cognitive status was defined using age-, sex-, and education-adjusted normative SDMT z-scores. The same comparison-specific covariate structure was used for both topological and connectivity analyses within each modality.

#### Correlation between network metrics and clinical variables

In Dataset 1, partial correlations were used to assess associations between network metrics and continuous clinical variables (EDSS and SDMT), whereas logistic regression was used for the categorical clinical variable of disease activity status (NEDA/EDA). Covariates were prespecified according to modality and clinical variable. For MRI-based analyses, associations with EDSS and SDMT were adjusted for age, sex, TIV, education, disease duration, and DMT status. For EEG-based analyses, associations were adjusted for age, sex, education, disease duration, and DMT status. Multiple comparisons were controlled using false discovery rate (FDR) correction.

### Role of funders

The funders had no role in study design; data collection, analysis, or interpretation; writing of the report; or the decision to submit the manuscript for publication.

## Results

### Demographic and clinical characteristics

Participant characteristics are summarised in Tables 1 and 2 and Supplementary Tables S1. Dataset 1 comprised 248 HC and 163 patients with MS; prespecified MRI subgroup analyses included high vs low disability (33 vs 130), cognitively impaired vs cognitively preserved (44 vs 119), EDA vs NEDA (69 vs 94), and a longitudinal NEDA subgroup (n = 33). Dataset 2 comprised 107 HC and 117 patients with MS. The hd-EEG subset of Dataset 1 included 80 HC and 135 patients with MS, with corresponding subgroup analyses for disability (108 vs 27), cognition (101 vs 34), and disease activity (78 vs 57). Detailed baseline characteristics for MRI and hd-EEG subgroups are provided in Supplementary Tables S1 and S13.

**Table 1:** Demographic and clinical characteristics of Dataset 1 from Xuanwu Hospital.

Variables	HC (n = 248)	MS (n = 163)	*p v*alue
Age at MRI, years, median (IQR)	35.0 (26.0–51.0)	34.0 (27.0–42.7)	0.160^a^
Sex, n (%)			0.928^b^
Female	176 (70.97)	114 (69.94)	
Male	72 (29.03)	49 (30.06)	
BMI, kg/m^2^, median (IQR)	23.03 (20.95–25.50)	22.48 (20.76–25.18)	0.445^a^
Education years, median (IQR)	15.0 (12.0–16.0)^d^	16.0 (12.0–16.0)	0.276^a^
Clinical phenotype, n (RRMS/SPMS/PPMS/CIS)	–	142/3/3/15	–
Disease duration, years, median (IQR)	–	3.5 (0.5–9.0)	–
EDSS at baseline, median (range)	–	2.00 (0.00–6.50)	–
SDMT z-score, mean ± SD	0.00 ± 1.00^e^	−0.39 ± 1.23	0.041^c^
DMT at baseline, n (%)	–		–
No		49 (30.06)	
Moderate^f^		54 (33.13)	
High^g^		60 (36.80)	
NEDA/EDA status at 1-year follow-up, n	–	94/69	–
TIV, cm^3^, mean ± SD	1442.00 ± 137.54	1403.90 ± 171.50	0.014^c^

**Abbreviations:** BMI, body mass index; CIS, clinically isolated syndrome; DMT, disease-modifying therapy; EDA, evidence of disease activity; EDSS, Expanded Disability Status Scale; HC, healthy controls; IQR, interquartile range; MRI, magnetic resonance imaging; MS, multiple sclerosis; NEDA, no evidence of disease activity; PPMS, primary progressive multiple sclerosis; RRMS, relapsing-remitting multiple sclerosis; SD, standard deviation; SDMT, Symbol Digit Modalities Test; SPMS, secondary progressive multiple sclerosis; TIV, total intracranial volume.

**Notes**: Data are presented as median (IQR), mean ± SD, or n (%), as appropriate. Disease-specific variables are presented for the MS group only. *P* values were not calculated for disease-specific variables. An em dash (−) indicates not applicable.

a *p* value derived from the Mann–Whitney U test (age at MRI, BMI, and years of education).

b *p* value derived from Pearson's chi-square test (sex).

c *p* value derived from the independent-samples t-test (SDMT z-score and TIV).

d Years of education were available for 204 healthy controls.

e SDMT z-scores were calculated using the mean and standard deviation of the 158 healthy controls with available SDMT data; therefore, the HC group has a mean of 0 and SD of 1 by definition.

f Moderate-efficacy DMTs included glatiramer acetate, interferon-beta, teriflunomide, dimethyl fumarate, and azathioprine.

g High-efficacy DMTs included fingolimod, natalizumab, rituximab, and mitoxantrone.

**Table 2:** Demographic and imaging characteristics of Dataset 2 from the UK Biobank.

Variables	HC (n = 107)	MS (n = 117)	*p* value
Age at MRI, years, median (IQR)	54.0 (48.0–60.0)	52.0 (46.0–57.0)	0.052^a^
Sex, n (%)			0.004^b^
Female	61 (57.01)	88 (75.21)	
Male	46 (42.99)	29 (24.79)	
BMI, kg/m^2^, median (IQR)	26.04 (23.53–28.43)	26.09 (23.17–29.77)	0.815^a^
TIV, cm^3^, mean ± SD	1526.41 ± 152.74	1460.63 ± 116.88	<0.001^c^

**Abbreviations:** BMI, body mass index; HC, healthy controls; IQR, interquartile range; MRI, magnetic resonance imaging; MS, multiple sclerosis; SD, standard deviation; TIV, total intracranial volume.

**Notes:** Data are presented as median (IQR), mean ± SD, or n (%), as appropriate.

a *p* value derived from the Mann–Whitney U test (age at MRI and BMI).

b *p* value derived from Pearson's chi-square test (sex).

c *p* value derived from the independent-samples t-test (TIV).

### Network topological abnormalities of iSCNs

**Fig. 2:**
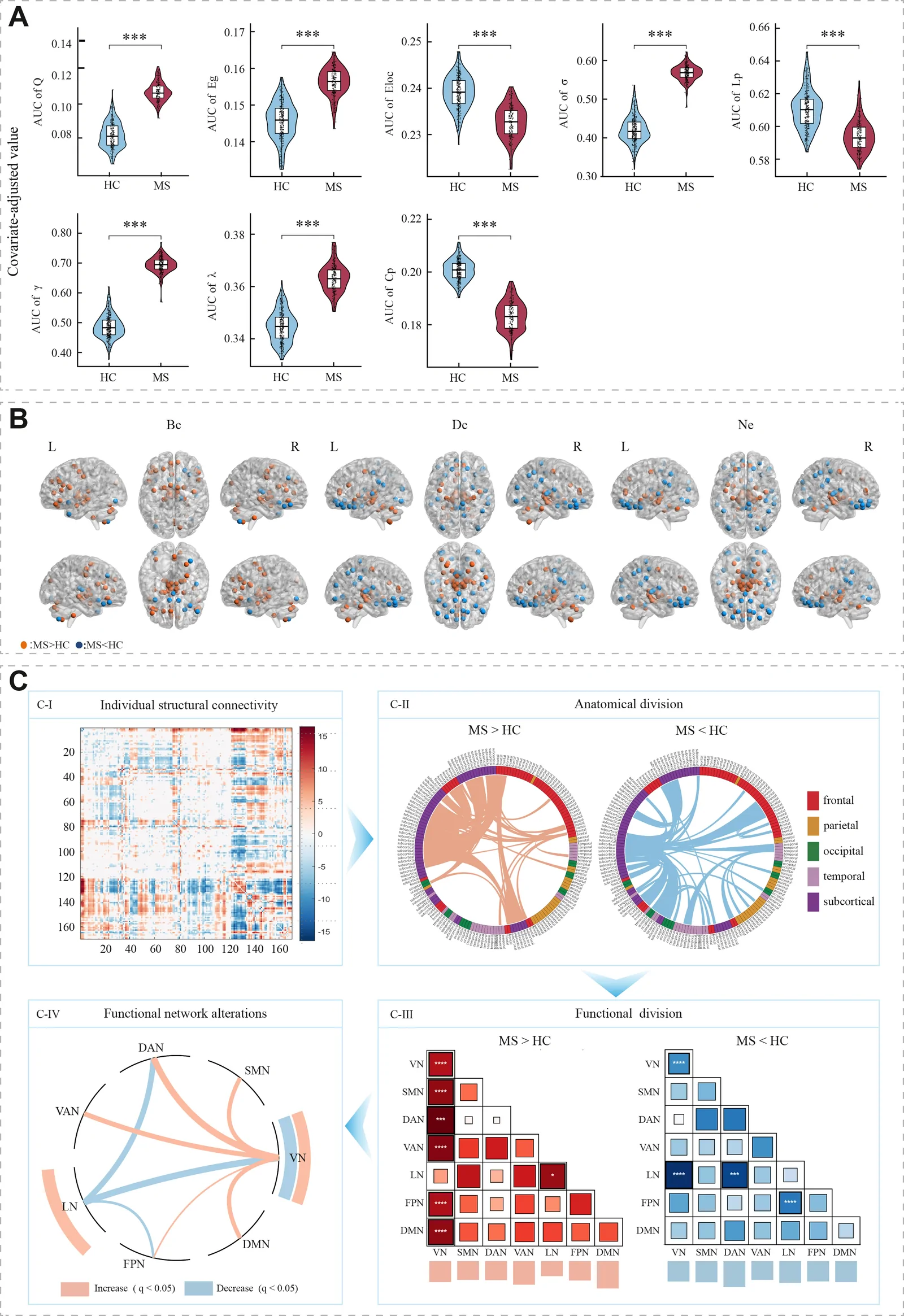
**MRI-derived iSCN alterations in MS** vs **healthy controls. (A)** Comparison of global topological network metrics between MS and HC. ∗∗∗: *q* < 0.001 (K = 0.10–0.40). **(B)** Surface maps showing the significant brain regions in nodal topological metrics. Orange indicates MS > HC, and blue indicates MS < HC. **(C)** Network connectivity abnormalities of iSCNs between HC and MS. **(C-I)** Edge-wise structural covariance connectivity results. Orange/red indicates MS > HC, and blue indicates MS < HC. **(C-II)** Focal connected network components and anatomical division of altered edges. **(C-III)** Functional-network matrices summarise the distribution of significant NBS-derived altered edges across seven canonical networks. Darker colours and larger squares indicate greater relative connection weight, calculated as the observed number of altered edges divided by the maximum possible number of edges for each network pair. Asterisks denote FDR-corrected q values from the network-pair analysis (∗: *q* < 0.05, ∗∗: *q* < 0.01, ∗∗∗: *q* < 0.001, ∗∗∗∗: *q* < 0.0001). The histograms below show the summed relative connection weight across network pairs. **(C-IV)** Direction of the functional-network alterations. Orange indicates MS > HC, and blue indicates MS < HC. **Abbreviations:** MS, multiple sclerosis; HC, healthy controls; iSCN, individualised structural covariance network; DMN, default mode network; FPN, frontoparietal network; VAN, ventral attention network; DAN, dorsal attention network; SMN, somatomotor network; LN, limbic network; VN, visual network. Eg, global efficiency; Eloc, local efficiency; σ, small-worldness; γ, normalised clustering coefficient; λ, normalised characteristic path length; Q, modularity; Cp, clustering coefficient; Dc, degree centrality; Ne, nodal efficiency; Bc, betweenness centrality; Lp, characteristic path length.

In Dataset 1, compared with HC, MS showed lower Cp, Lp, and Eloc, together with higher γ, λ, σ, Q, and Eg (all *q* < 0.001), whereas r was not significant (Fig. 2A; Supplementary Tables S4 and S5). At the nodal level, MS > HC effects were most prominent in precentral and medial frontal regions, as well as in subcortical and cerebellar nodes, particularly the amygdala, pallidum, medial geniculate, pulvinar, mediodorsal thalamus, and red nucleus. By contrast, MS < HC effects were concentrated mainly in orbitofrontal, posterior cingulate, and occipital regions, with the strongest decreases involving the lateral orbital gyrus, posterior cingulate gyrus, and lingual gyrus (Fig. 2B; Supplementary Figure S4, Supplementary Tables S2, S4, and S7).

**Fig. 3:**
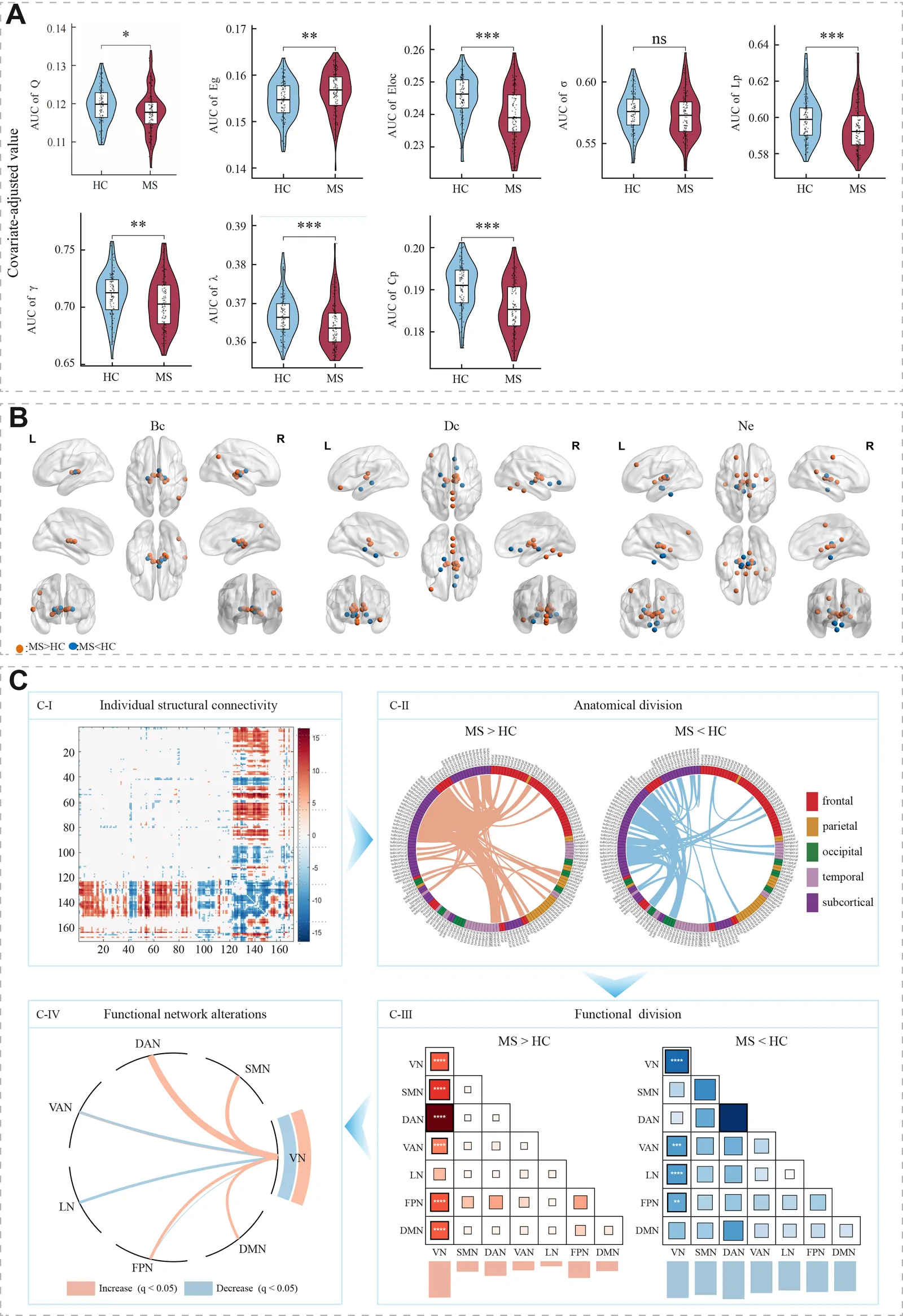
**MRI-derived iSCN alterations in the UK Biobank MS cohort. (A)** Comparison of global topological network metrics between MS and HC. ∗: *q* < 0.05, ∗∗: *q* < 0.01, ∗∗∗: *q* < 0.001, ∗∗∗∗: *q* < 0.0001, ns, not significant (K = 0.10–0.40). **(B)** Surface maps showing the significant brain regions in nodal topological metrics. Orange indicates MS > HC, and blue indicates MS < HC. **(C)** Network connectivity abnormalities of iSCN between HC and MS. **(C-I)** Edge-wise structural covariance connectivity results. Orange indicates MS > HC, and blue indicates MS < HC. **(C-II)** Focal connected network components and anatomical division of altered edges. **(C-III)** Functional-network matrices summarise the distribution of significant NBS-derived altered edges across seven canonical networks. Darker colours and larger squares indicate greater relative connection weight, calculated as the observed number of altered edges divided by the maximum possible number of edges for each network pair. Asterisks denote FDR-corrected *q* values from the network-pair analysis (∗: *q* < 0.05, ∗∗: *q* < 0.01, ∗∗∗: *q* < 0.001, ∗∗∗∗: *q* < 0.0001). The histograms below show the summed relative connection weight across network pairs. **(C-IV)** Direction of the functional-network alterations. Orange indicates MS > HC, and blue indicates MS < HC**. Abbreviations:** MS, multiple sclerosis; HC, healthy controls; iSCN, individualised structural covariance network; DMN, default mode network; FPN, frontoparietal network; VAN, ventral attention network; DAN, dorsal attention network; SMN, somatomotor network; LN, limbic network; VN, visual network. Eg, global efficiency; Eloc, local efficiency; σ, small-worldness; γ, normalised clustering coefficient; λ, normalised characteristic path length; Q, modularity; Cp, clustering coefficient; Dc, degree centrality; Ne, nodal efficiency; Bc, betweenness centrality; Lp, characteristic path length.

In Dataset 2, MS showed higher Eg and lower Lp, γ, λ, Cp, Eloc, and Q than HC, while r and σ were nonsignificant (Fig. 3A; Supplementary Table S6). Nodal abnormalities were predominantly subcortical, especially in thalamic subnuclei. MS > HC effects involved bilateral mediodorsal and pulvinar thalamic subdivisions, with additional pallidal, cingulate, precentral, and dorsolateral superior frontal involvement. MS < HC effects were less extensive, centred on the bilateral inferior pulvinar, with additional decreases in the locus coeruleus, substantia nigra pars compacta, and other thalamic nuclei (Fig. 3B; Supplementary Table S3).

**Fig. 4:**
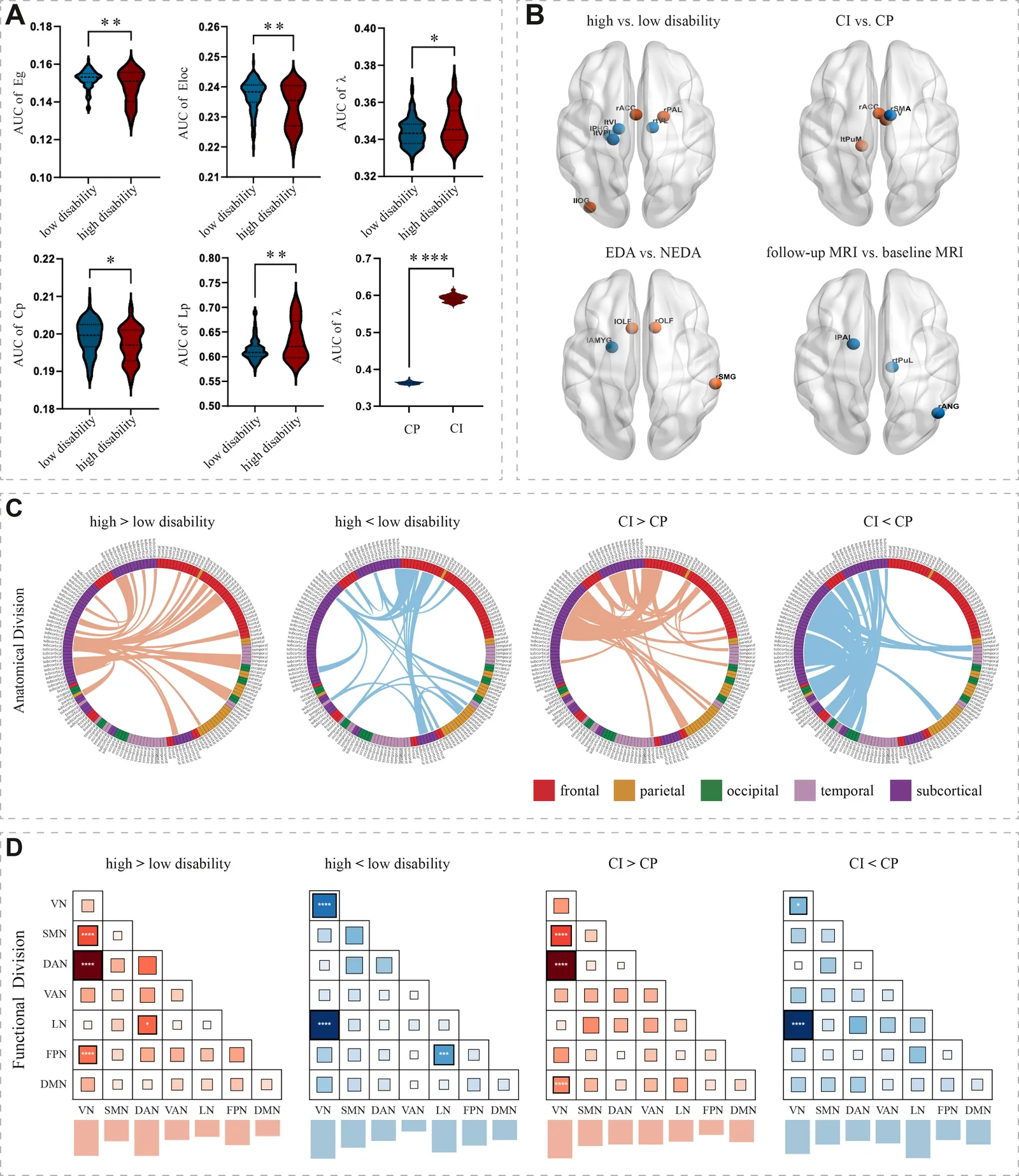
**MRI-derived iSCN alterations across MS subgroups. (A)** Comparisons of global topological network metrics in disability stratification and cognitive stratification. ∗: *q* < 0.05, ∗∗: *q* < 0.01, ∗∗∗∗: *q* < 0.0001. **(B)** Significant nodal topological abnormalities in disability stratification (high vs low disability), cognitive stratification (CI vs CP), disease-activity stratification (EDA vs NEDA), and the longitudinal NEDA subgroup (follow-up MRI vs baseline MRI). Orange indicates higher values in the first-mentioned group, and blue indicates lower values in the first-mentioned group. **(C)** Circos plots showing the anatomical distribution of significantly altered structural covariance connectivity in disability stratification and cognitive stratification. Brain regions are colour-coded by anatomical division (red = frontal, orange = parietal, green = occipital, blue = temporal, purple = subcortical). Orange edges indicate higher connectivity in the first-mentioned group, and blue edges indicate lower connectivity in the first-mentioned group. **(D)** Functional-network matrices summarise the distribution of significant NBS-derived altered edges across seven canonical networks (VN, SMN, DAN, VAN, LN, FPN, and DMN). Darker colours and larger squares indicate higher relative connection weight, defined as the observed number of altered edges divided by the maximum possible number of edges for each network pair. Asterisks denote FDR-corrected *q* values from the network-pair analysis (∗: *q* < 0.05, ∗∗: *q* < 0.01, ∗∗∗: *q* < 0.001, ∗∗∗∗: *q* < 0.0001). The histograms below show the summed relative connection weight across network pairs. **Abbreviations:** CI, cognitively impaired; CP, cognitively preserved; NEDA, no evidence of disease activity; EDA, evidence of disease activity; DMN, default mode network; FPN, frontoparietal network; VAN, ventral attention network; DAN, dorsal attention network; SMN, somatomotor network; LN, limbic network; VN, visual network. Eg, global efficiency; Eloc, local efficiency; λ, normalised characteristic path length; Cp, clustering coefficient; Lp, characteristic path length.

In disability stratification, the high-disability group showed higher λ (Cohen's *d* = 0.12, *q* = 0.04), Lp (*d* = 0.32, *q* = 0.001), and Eg (*d* = 0.20, *q* = 0.009), together with lower Cp (*d* = −0.12, *q* = 0.040) and Eloc (*d* = −0.25, *q* = 0.008); γ, σ, and Q were not significant after correction (Fig. 4A; Supplementary Table S4). Nodal abnormalities involved seven regions: increased nodal degree in the right anterior cingulate/paracingulate gyrus (*d* = 0.60, *q* = 0.011) and left inferior occipital gyrus (*d* = 0.56, *q* = 0.013), increased nodal efficiency in the right pallidum (*d* = 0.63, *q* = 0.003), reduced nodal betweenness in the left parahippocampal gyrus (*d* = −0.60, *q* = 0.011), reduced nodal degree in the left ventral lateral thalamus (*d* = −0.68, *q* < 0.001) and right ventral lateral thalamus (*d* = −0.52, *q* = 0.010), and reduced nodal efficiency in the left ventral posterolateral thalamus (*d* = −0.49, *q* = 0.016) (Fig. 4B; Supplementary Table S8).

In cognitive stratification, the CI group showed higher λ than the CP group. Nodal abnormalities were limited to four regions: increased nodal degree in the right anterior cingulate/paracingulate gyrus (*d* = 0.60, *q* = 0.037), increased nodal efficiency in the left pulvinar medial (*d* = 0.65, *q* = 0.021) and right thalamic anteroventral nucleus (*d* = 0.64, *q* = 0.020), and decreased nodal efficiency in the right supplementary motor area (*d* = −0.70, *q* = 0.019) (Fig. 4A and B; Supplementary Table S9).

In disease-activity stratification, no global topological metric survived correction. Nodal abnormalities involved four regions: increased nodal betweenness and nodal degree in the left olfactory cortex (*d* = 0.59, *q* = 0.022; *d* = 0.44, *q* = 0.033, respectively), increased nodal efficiency in the right olfactory cortex (*d* = 0.59, *q* = 0.022), increased nodal betweenness in the right supramarginal gyrus (*d* = 0.62, *q* = 0.023), and decreased nodal degree in the left amygdala (*d* = −0.48, *q* = 0.031) (Fig. 4B; Supplementary Table S10).

### Longitudinal NEDA subgroup

In this subgroup, significant longitudinal changes were observed only at the nodal level, including reduced nodal degree in the right angular gyrus (*d* = −0.65, *q* = 0.045), reduced nodal betweenness and nodal efficiency in the left pallidum (*d* = −0.76, *q* = 0.021; *d* = −0.62, *q* = 0.049, respectively), and reduced nodal betweenness in the right lateral pulvinar (*d* = −0.69, *q* = 0.035) (Supplementary Table S11).

### Network connectivity abnormalities of iSCNs

In Dataset 1, altered edges were broadly distributed across frontal, temporal, parietal, occipital, and subcortical divisions (Fig. 2C-I–II). At the connectivity level, NBS identified one increased component and one decreased component (both *p*_FWE_ = 0.001). The increased component involved a broad VN-centred increased pattern, particularly involving DMN–VN (584 edges, 83 nodes), VN–VN (526 edges, 55 nodes), FPN–VN (426 edges, 77 nodes), SMN–VN (374 edges, 73 nodes), and VAN–VN (362 edges, 73 nodes), with additional DAN–VN and LN–LN involvement. The decreased pattern mainly involved LN–VN (694 edges, 80 nodes), VN–VN (565 edges, 56 nodes), FPN–LN (209 edges, 45 nodes), and LN–DAN (58 edges, 21 nodes) (all *q* < 0.05) (Fig. 2C-III–IV).

In the external validation cohort (Dataset 2), altered edges were also distributed across cortical and subcortical regions (Fig. 3C-I–II). At the connectivity level, NBS identified one increased component and one decreased component (both *p*_FWE_ < 0.001). The increased component involved VN–VN (460 edges, 55 nodes), SMN–VN (363 edges, 52 nodes), DAN–VN (150 edges, 40 nodes), VAN–VN (228 edges, 52 nodes), FPN–VN (379 edges, 66 nodes), and DMN–VN (471 edges, 69 nodes). The decreased component included VN–VN (501 edges, 53 nodes), VAN–VN (246 edges, 69 nodes), LN–VN (346 edges, 77 nodes), and FPN–VN (294 edges, 73 nodes). VN–VN was significant in both directions (all q < 0.05) (Fig. 3C-III–IV).

In disability stratification, altered edges involved frontal-subcortical, frontoparietal, frontotemporal, fronto-occipital, and posterior cortical-subcortical connections (Fig. 4C). NBS identified one increased component (*p*_FWE_ = 0.0010) and one decreased component (*p*_FWE_ = 0.0050). Functional-network decomposition showed that the increased component involved significant SMN-VN (235 edges, 54 nodes, *q* < 0.0001), DAN-VN (109 edges, 38 nodes, *q* < 0.0001), LN-DAN (25 edges, 16 nodes, *q* = 0.012), and FPN-VN (242 edges, 68 nodes, *q* < 0.0001) blocks. The decreased component mainly involved VN-VN (291 edges, 53 nodes, *q* < 0.0001), LN-VN (330 edges, 63 nodes, *q* < 0.0001), and FPN-LN (78 edges, 30 nodes, *q* = 0.000638) blocks (Fig. 4D).

In cognitive stratification, altered edges involved subcortical-frontal, subcortical-temporal, subcortical-parietal, and subcortical-occipital connections, with additional intra-frontal involvement (Fig. 4C). NBS identified one increased component (*p*_FWE_ = 0.0030) and one decreased component (*p*_FWE_ = 0.0050). Yeo 7-network decomposition showed that the increased pattern mainly involved SMN-VN (181 edges, 61 nodes, *q* < 0.0001), DAN-VN (81 edges, 30 nodes, *q* < 0.0001), and DMN-VN (197 edges, 60 nodes, *q* < 0.0001), whereas the decreased pattern mainly involved VN-VN (166 edges, 49 nodes, *q* = 0.0304) and LN-VN (307 edges, 71 nodes, *q* < 0.0001) (Fig. 4D).

No significant network connectivity abnormalities were identified for disease-activity stratification.

### Common and specific network metrics of MRI and EEG

**Fig. 5:**
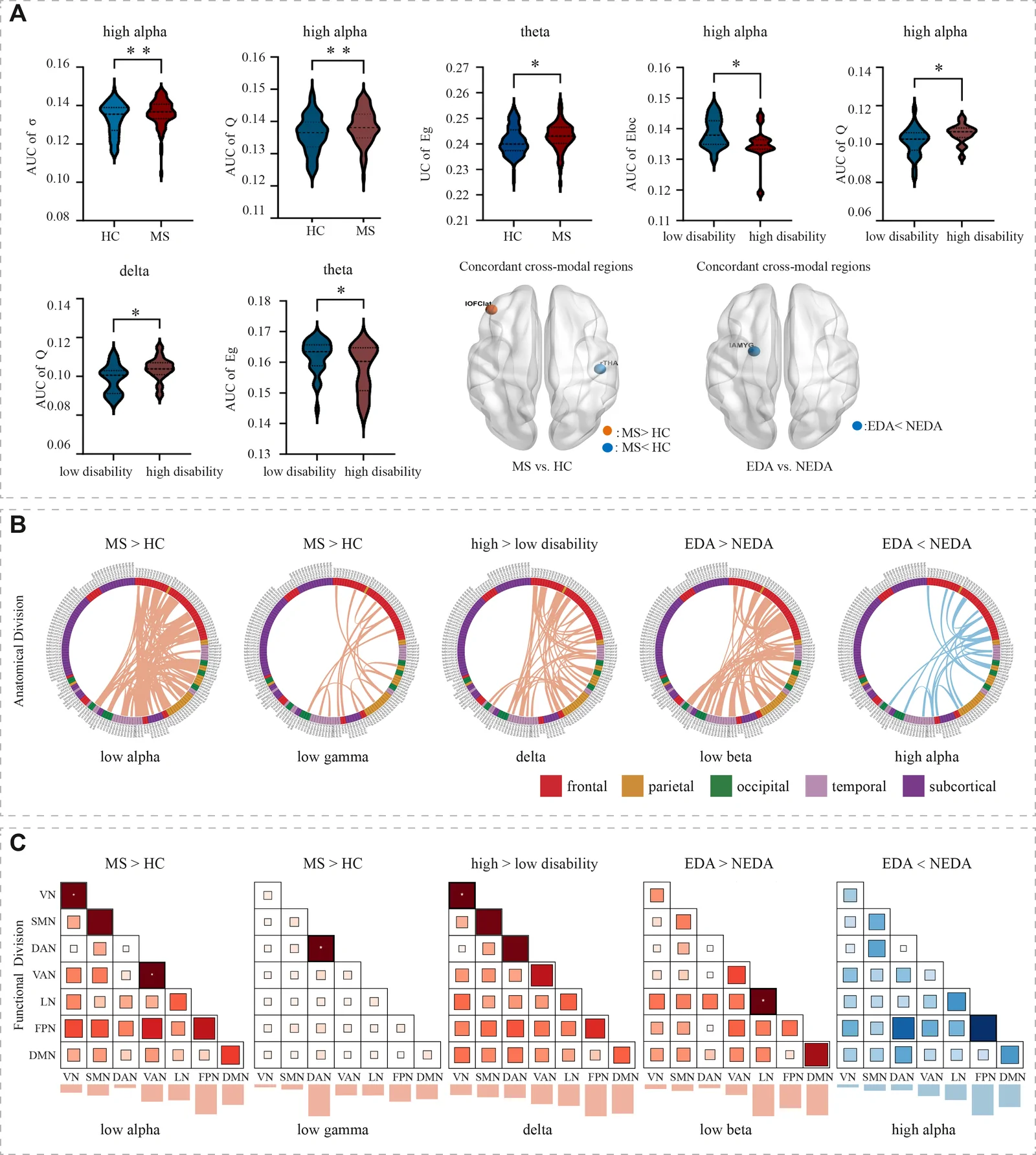
**Common and specific network abnormalities identified by MRI and hd-EEG. (A)** Concordant cross-modal alterations in global and nodal topological metrics. Violin plots show frequency-specific hd-EEG global metrics with effects in the same direction as the corresponding MRI-derived abnormalities. In the MS vs HC comparison, hd-EEG showed higher AUC of σ and Q in the high-alpha band and higher AUC of Eg in the theta band in MS. In the high-vs low-disability comparison, hd-EEG showed lower AUC of Eloc in the high-alpha band, higher AUC of Q in the high-alpha and delta bands, and lower AUC of Eg in the theta band in the high-disability group. Brain surface renderings show concordant nodal abnormalities across MRI and hd-EEG, including a left lateral orbital region (MS > HC) and a right thalamic region (MS < HC) in the MS-vs-HC comparison, and a left amygdala region (EDA < NEDA) in the EDA-vs-NEDA comparison. Orange indicates higher nodal metric values in the first group, and blue indicates lower nodal metric values in the first group. (∗: *q* < 0.05, ∗∗: *q* < 0.01). **(B)** Circos plots show the anatomical distribution of significantly altered hd-EEG functional connections for the comparisons and frequency bands indicated: MS > HC in the low alpha band, MS > HC in the low gamma band, high > low disability in the delta band, EDA > NEDA in the low beta band, and EDA < NEDA in the high alpha band. Brain regions are colour-coded by anatomical division (frontal, parietal, occipital, temporal, and subcortical). Orange edges indicate stronger connectivity in the first-mentioned group, whereas blue edges indicate weaker connectivity. **(C)** Functional-network matrices summarise the distribution of significant NBS-derived altered hd-EEG connections across seven canonical networks (VN, SMN, DAN, VAN, LN, FPN, and DMN). Colour intensity and square size represent the relative connection weight, calculated as the observed number of altered edges divided by the maximum possible number of edges for each network pair. Orange indicates increased connectivity and blue indicates decreased connectivity in the first-mentioned group. Asterisks denote FDR-corrected q values from the network-pair analysis (∗: *q* < 0.05, ∗∗: *q* < 0.01, ∗∗∗: *q* < 0.001, ∗∗∗∗: *q* < 0.0001). Bars below the matrices summarise the total relative connection weight across network pairs. **Abbreviations:** MRI, magnetic resonance imaging; hd-EEG, high-density electroencephalography; MS, multiple sclerosis; HC, healthy controls; EDA, evidence of disease activity; NEDA, no evidence of disease activity; VN, visual network; SMN, somatomotor network; DAN, dorsal attention network; VAN, ventral attention network; LN, limbic network; FPN, frontoparietal network; DMN, default mode network; Eg, global efficiency; Eloc, local efficiency; σ, small-worldness; Q, modularity.

In the multimodal subset, MRI and hd-EEG showed partly overlapping abnormalities (Fig. 5). In the MS-vs-HC comparison, hd-EEG showed higher Eg in the theta band and higher σ and Q in the high-alpha band, together with increased connectivity in the low-alpha and low-gamma bands. Across subgroup analyses, the high-disability group showed the most extensive hd-EEG abnormalities, involving global, nodal, and connectivity levels, whereas cognitive stratification was more focal and disease-activity stratification showed nodal and frequency-specific connectivity abnormalities without significant global changes. Cross-modal nodal overlap was observed in the lateral orbitofrontal cortex (MS > HC) and right thalamic (MS < HC) region in the MS-vs-HC comparison, and in the left amygdala (EDA < NEDA) in the EDA-vs-NEDA comparison (Fig. 5A). Detailed hd-EEG results are provided in Supplementary Results S4.

### Correlation between network metrics and clinical variables

**Fig. 6:**
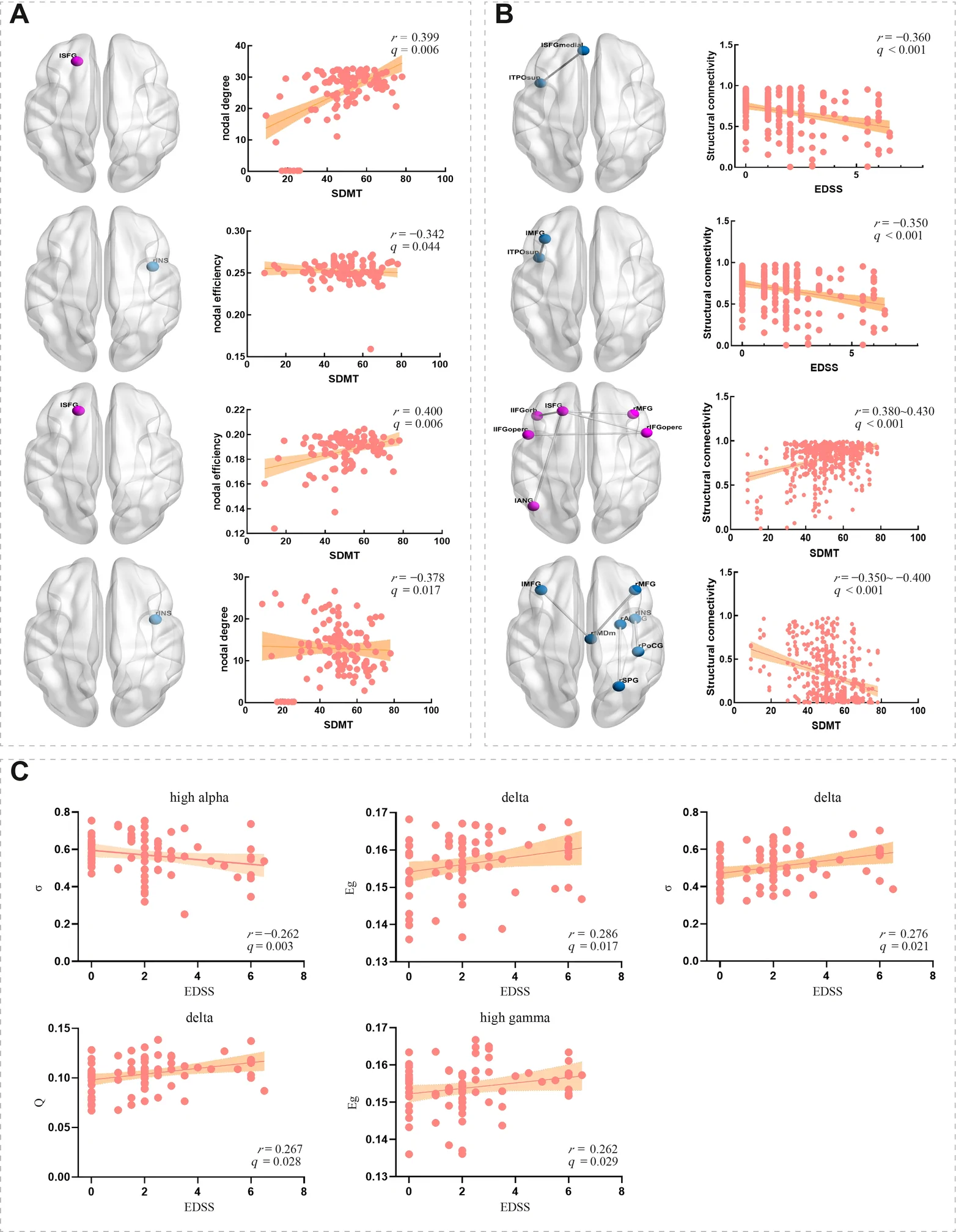
**Correlation between network metrics and clinical variables. (A)** MRI-derived nodal topological metrics showing significant partial correlations with SDMT. Analyses were adjusted for age, sex, TIV, education, disease duration, and DMT status. The nodal degree and nodal efficiency of the lSFG were significantly positively correlated with SDMT scores, respectively (*r* = 0.399, *q* = 0.006; *r* = 0.400, *q* = 0.006). In contrast, the nodal degree and nodal efficiency of the rINS were significantly negatively correlated with SDMT scores, respectively (*r* = −0.378, *q* = 0.017; *r* = −0.342, *q* = 0.044). Scatterplots show the direction and magnitude of these associations. **(B)** MRI-derived structural connectivity showing significant associations with disability and cognition. Analyses were adjusted for age, sex, TIV, education, disease duration, and DMT status. Connections between lSFGmedial–rSTG (*r* = −0.360, *q* < 0.001) and lMFG–rSTG (*r* = −0.350, *q* < 0.001) were negatively correlated with EDSS. Positive associations with SDMT were observed for lSFG–rMFG, lSFG–rIFGoperc, lIFGoperc–rIFGoperc, lSFG–lIFGorb, and lSFG–lANG (*r* = 0.380–0.430, *q* < 0.001). Negative associations with SDMT were observed for rPoCG–rINS, rSPG–rAMYG, lMFG–rtMDm, and rMFG–rtMDm (*r* = −0.350 to −0.400, *q* < 0.001). Brain surface renderings highlight the principal regions contributing to these associations. **(C)** hd-EEG-derived global topological metrics showing frequency-specific associations with disability severity. Analyses were adjusted for age, sex, education, disease duration, and DMT status. Significant partial correlations between EDSS and EEG metrics, including σ, Eg, and Q, are shown with fitted regression lines and 95% confidence intervals. **Abbreviations:** DMT, disease-modifying therapy; EDSS, Expanded Disability Status Scale; Eg, global efficiency; hd-EEG, high-density electroencephalography; lANG, left angular gyrus; lIFGoperc, left inferior frontal gyrus, opercular part; lIFGorb, left inferior frontal gyrus, orbital part; lMFG, left middle frontal gyrus; lSFG, left superior frontal gyrus; lSFGmedial, left superior frontal gyrus, medial part; *q*, FDR-corrected *p* value; Q, modularity; rAMYG, right amygdala; rINS, right insula; rMFG, right middle frontal gyrus; rPoCG, right postcentral gyrus; rSPG, right superior parietal gyrus; rSTG, right superior temporal gyrus; rtMDm, right mediodorsal thalamus, medial magnocellular part; SDMT, Symbol Digit Modalities Test; σ, small-worldness; TIV, total intracranial volume.

MRI-derived nodal metrics were significantly associated with SDMT. Nodal degree and nodal efficiency in the left superior frontal gyrus were positively correlated with SDMT (*r* = 0.399, *q* = 0.006; *r* = 0.400, *q* = 0.006), whereas nodal degree and nodal efficiency in the right insula were negatively correlated with SDMT (*r* = −0.378, *q* = 0.017; *r* = −0.342, *q* = 0.044) (Fig. 6A). At the connectivity level, the left superior frontal gyrus-medial–right superior temporal gyrus and left middle frontal gyrus–right superior temporal gyrus connections were negatively correlated with EDSS (both *q* < 0.001). Associations with SDMT were also observed across frontal, frontotemporal, parietal–insular, amygdalar, and thalamic-related connections, in both positive and negative directions (Fig. 6B). In hd-EEG, EDSS correlated negatively with high-alpha σ (*r* = −0.262, *q* = 0.003) and positively with delta-band Eg/σ/Q and high-gamma Eg (*r* = 0.262–0.286, *q* = 0.017–0.029) (Fig. 6C).

### Individual-level classification performance

Six classification tasks were evaluated; full performance metrics are summarised in Supplementary Table S19.

**Fig. 7:**
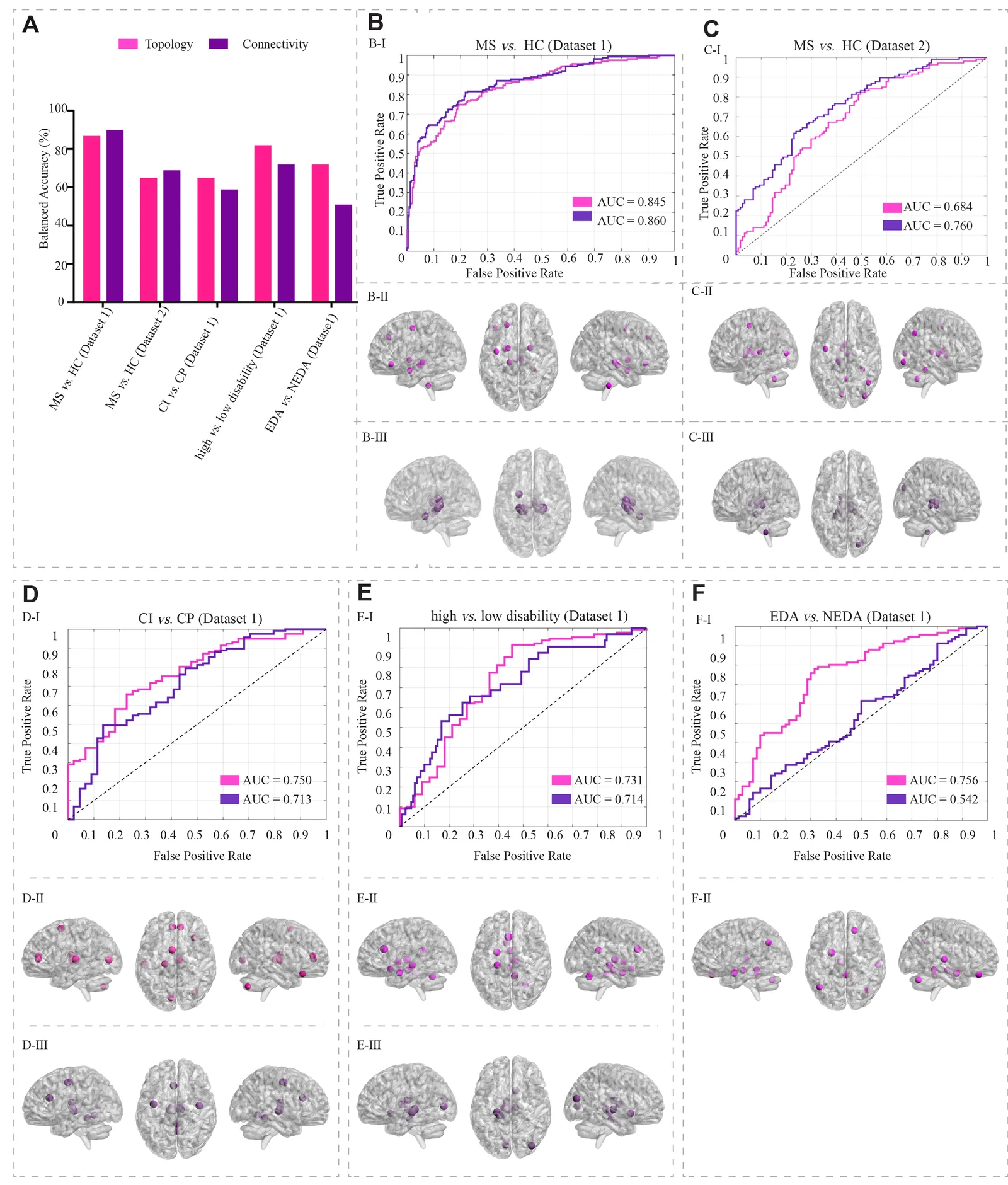
**MRI-based classification performance and regional feature-weight maps of topology- and connectivity-based iSCN models. (A)** Comparison of balanced accuracy between topology-based and connectivity-based models across five MRI-based classification tasks: MS vs HC in Dataset 1, MS vs HC in Dataset 2, CI vs CP in Dataset 1, high vs low disability in Dataset 1, and EDA vs NEDA in Dataset 1. **(B-I, C-I, D-I, E-I, F-I)** Cross-validated receiver operating characteristic (ROC) curves showing the classification performance of topology-based and connectivity-based models for each task**. (B-II, C-II, D-II, E-II, F-II)** Brain maps showing the top contributing regions in the topology-based models, as identified by regional feature-weight mapping**. (B-III, C-III, D-III, E-III)** Brain maps showing the top contributing regions in the connectivity-based models, as identified by regional feature-weight mapping. No corresponding F-III panel is shown for the EDA vs NEDA task because the connectivity-based model showed limited discriminative performance and no robust regional contribution pattern. **Abbreviations:** MS, multiple sclerosis; HC, healthy controls; CI, cognitively impaired; CP, cognitively preserved; EDA, evidence of disease activity; NEDA, no evidence of disease activity; ROC, receiver operating characteristic; AUC, area under the ROC curve.

For MS-vs-HC classification, in Dataset 1 topological features achieved an accuracy of 84.8% (95% CI: 80.6–88.6%, AUC = 0.845), whereas connectivity features achieved a higher accuracy of 89.5% (95% CI: 86.2–92.1%, AUC = 0.860) (Fig. 7B). In Dataset 2, topological features achieved an accuracy of 65.2% (95% CI: 58.7–71.1%, AUC = 0.684), whereas connectivity features achieved a higher accuracy of 75.0% (95% CI: 68.9–80.2%, AUC = 0.760) (Fig. 7C).

For disability stratification, topological features achieved an accuracy of 82.1% (95% CI: 75.5–87.2%, AUC = 0.731), compared with 75.9% (95% CI: 68.8–81.9%, AUC = 0.714) for connectivity features (Fig. 7E). For cognitive stratification, topological features achieved an accuracy of 75.2% (95% CI: 66.8–80.6%, AUC = 0.750), compared with 71.8% (95% CI: 64.4–78.1%, AUC = 0.713) for connectivity features (Fig. 7D). For disease-activity stratification, topological features achieved an accuracy of 70.8% (95% CI: 63.4–77.3%, AUC = 0.756), whereas connectivity features showed limited discrimination, with an accuracy of 56.4% (95% CI: 47.5–62.6%, AUC = 0.542) (Fig. 7F). Across MRI models with interpretable contribution maps, discriminative regions were centred mainly on thalamic and related subcortical structures, together with anterior cingulate, frontal, pallidal, and cerebellar regions (Fig. 7; Supplementary Tables S20–S24).

EEG-based MS-vs-HC classification results, including the band-specific analysis, are presented in Supplementary Results S5, Supplementary Figure S2, and Supplementary Table S19.

### Reproducibility and sensitivity analyses

The principal findings were broadly retained in the BN246 reproducibility analysis and in the JSD-based sensitivity analysis, although the latter showed a less extensive pattern. Additional density-range sensitivity analyses using K = 0.20–0.50 and K = 0.30–0.60 showed partial robustness to threshold selection, with relatively consistent modularity findings but density-dependent variation in several other global metrics. Detailed results are provided in Supplementary Results S1–S3, Supplementary Figures S5 and S6, and Supplementary Table S26.

## Discussion

Using two independent MRI cohorts and a multimodal subset, this study investigated the topological and connectional profiles of grey matter structural networks in multiple sclerosis, and examined their relevance to diagnosis, clinical stratification, longitudinal change, and cross-modal findings. First, MRI-based iSCNs showed abnormalities at the global, nodal, and connectivity levels that were broadly reproduced in the external validation cohort and in the reproducibility and sensitivity analyses. Second, these abnormalities converged on thalamic and other subcortical hubs, together with insular, cingulate, frontotemporal, and visual-related systems. Third, different clinical dimensions mapped onto different network scales: disability showed the broadest abnormalities, whereas cognition and disease activity were associated with more selective changes. Fourth, complementary analyses extended the main MRI-based findings in two ways. hd-EEG provided partial cross-modal support for the observed network abnormalities, whereas machine-learning analyses suggested that connectivity features were more informative for diagnosis and topological features for within-disease stratification. Together, these findings support the view that MS is not only a lesion-based inflammatory disease, but also a disorder of large-scale network remodelling,^38^ and suggest that iSCNs may provide a sensitive marker of network change.

Overall, our results suggest that MS involves brain network reorganisation rather than a simple overall loss of connectivity. This was supported across the discovery cohort, validation cohort, and sensitivity analyses, where iSCNs showed convergent global, nodal, and connectivity abnormalities.^39^, ^40^, ^41^ Repeated involvement of thalamic and other subcortical regions, together with insular and cingulate systems, suggests preferential alteration of communication and integration hubs.^42^ In both MRI cohorts, altered edges and enriched network-pair blocks were concentrated in visual-network-related couplings.^43^, ^44^, ^45^ The coexistence of increased and decreased connectivity indicates selective redistribution across systems, with the visual network emerging as a prominent site of reorganisation.^46^

Different clinical dimensions in MS were associated with distinct network profiles. Disability showed the broadest abnormalities across global, nodal, and connectivity levels, consistent with more widespread disruption of brain network organisation. Cognitive impairment was more selective, mainly involving thalamic, cingulate, and supplementary motor regions with altered visual-network-related coupling, suggesting involvement of relay and control systems rather than diffuse global change.^47^^,^^48^ Disease-activity stratification was more circumscribed, with no corrected global effects and nodal changes centred mainly on the olfactory cortex, supramarginal gyrus, and amygdala. The longitudinal NEDA analysis should be interpreted cautiously because of the modest sample size, but it indicates that clinical and radiological stability defined by NEDA-3 does not necessarily imply complete network stability.^49^^,^^50^

Correlation analyses supported the clinical relevance of these findings. Better cognition was associated with frontal integrative metrics, whereas greater disability was linked to thalamo-cortical and frontotemporal connectivity. hd-EEG showed partly convergent disability associations.^33^^,^^51^

Machine-learning analyses added patient-level relevance beyond group comparisons. Across two MRI cohorts, connectivity features performed best for MS-vs-HC classification, whereas topological features better separated disability, cognition, and disease activity. This pattern is biologically plausible: connectivity features preserve fine-grained distributed abnormalities that may be more sensitive to case–control differences, whereas topological metrics summarise integration, segregation, and hub organisation, and may better capture within-disease heterogeneity.^52^^,^^53^ The replication of the connectivity-over-topology pattern in the independent UK Biobank cohort argues against a cohort-specific finding. The most informative regions clustered in thalamic relay nuclei, cingulate-frontal control areas, visual pathways, cerebellar nodes, and nigral-limbic structures, suggesting distributed prediction rather than isolated lesion effects.^53^ Because neuroimaging features are correlated, linear SVM weights should be interpreted as descriptive model-contribution indicators, not independent measures of isolated regional importance. EEG-based classification was weaker, consistent with the greater frequency dependence and functional variability of electrophysiological signals.^54^ At this stage, the SVM analyses should be regarded as exploratory biomarker evaluations rather than locked or deployable clinical prediction models intended for use by clinicians or patients; no clinical interface or end-user workflow was developed, and no formal fairness analysis was performed.

hd-EEG provided a complementary functional readout rather than direct validation. Concordant global and nodal abnormalities supported the physiological relevance of selected MRI findings, whereas frequency-specific connectivity changes, especially in disability and disease activity, suggested that functional network expression is more dynamic and state-sensitive than structural covariance.^55^, ^56^, ^57^

These findings may have practical implications. Because iSCNs were derived from routine structural MRI rather than specialised acquisition sequences, they may be feasible for translational workflows.^58^^,^^59^ However, clinical implementation will require standardised preprocessing, prospective validation, greater automation, and explicit feasibility assessment.^60^^,^^61^

Key strengths of this study include the use of two independent MRI cohorts, external validation, multimodal hd-EEG analyses, and complementary reproducibility and sensitivity analyses. Several limitations should be noted. First, follow-up mainly captured early subclinical progression, and longer longitudinal studies are needed to define trajectories across disease stages. Second, the external validation cohort lacked detailed clinical characterisation, limiting validation of subgroup stratification. Third, the Yeo atlas served as a systems-level reference framework rather than a direct correspondence between structural covariance and intrinsic functional networks.^62^ Fourth, hd-EEG has limited sensitivity to deep grey matter, so subcortical, particularly thalamic, findings should be interpreted cautiously.^63^^,^^64^ Fifth, because small-worldness was considered when defining the primary density range, σ-related findings should be interpreted cautiously. Additional density-range sensitivity analyses showed partial, but not uniform, robustness to threshold selection: the modularity finding was relatively consistent across density ranges, whereas several other global metrics, including small-worldness-related measures, varied across density ranges. Sixth, although TIV was included as a covariate in group-level analyses and each ROI was represented by a normalised probability density function, global GM effects were not explicitly residualised before iSCN construction. Residual influences of global GM atrophy or ROI-size-related stability of density estimation on some network measures therefore cannot be fully excluded.

Within these constraints, our findings suggest that the iSCN approach can capture clinically meaningful brain reorganisation in MS and may help link routine MRI with future patient-level stratification.

## Contributors

RPG and JWH designed the study, developed the methodology, performed the formal analyses, and drafted the manuscript. XGS, HYZ, YM, JJL, YTW, and YQ collected and curated the data. RPG performed statistical analyses and machine-learning modelling, and generated the figures. JS, YNZ, and JWH supervised the study, interpreted the analyses, and critically revised the manuscript. RPG and JWH accessed and verified the underlying data and were responsible for the decision to submit the manuscript. All authors interpreted the data, read and revised the manuscript, and approved the final version.

## Data sharing statement

De-identified participant-level data underlying the reported results, including the demographic and clinical variables used in the analyses and derived MRI and hd-EEG measures, will be available after publication from the corresponding author upon reasonable request. Requests will be considered on a case-by-case basis from qualified researchers for bona fide research purposes and will require a methodologically sound proposal, any necessary ethical and institutional approvals, and completion of a data-use agreement. Raw MRI and hd-EEG data from Dataset 1 will not be publicly deposited because of participant privacy, consent, and institutional restrictions; requests for controlled access may be considered where permitted by the relevant approvals. Data from UK Biobank cannot be redistributed by the authors and should be obtained directly through the standard UK Biobank access procedure. Custom analysis code used for iSCN construction, statistical analyses, and SVM modelling will be available from the corresponding author upon reasonable request, subject to institutional approval and any applicable third-party software or licencing restrictions.

## Declaration of interests

The authors declare no competing interests.
