# Supplementary material for: Brain network biomarkers for diagnosis and clinical stratification in multiple sclerosis: a longitudinal study integrating individualised structural covariance MRI with high-density EEG
Source: eBioMedicine. 2026 Aug 19;131:106438. doi: 10.1016/j.ebiom.2026.106438 (PMC13520990; doi:10.1016/j.ebiom.2026.106438)
Supplement: Supplementary file 1 [file mmc1.docx]

**Supplementary Materials**

[I Supplementary Methods	3](#_Toc227070408)

[Supplementary Method S1. Inclusion and exclusion criteria	3](#_Toc227070409)

[Supplementary Method S2. MRI acquisition and analysis	4](#_Toc227070410)

[Supplementary Method S3. Structural network construction	6](#_Toc227070411)

[Supplementary Method S4. Network topological metrics	7](#_Toc227070412)

[Supplementary Method S5.Analysis of connectivity patterns of iSCN	8](#_Toc227070413)

[Supplementary Method S6. Support vector machine	9](#_Toc227070414)

[Supplementary Method S7. EEG acquisition and analysis	10](#_Toc227070415)

[II Supplementary Tables	12](#_Toc227070416)

[Supplementary Table S1	12](#_Toc227070417)

[Supplementary Table S2](#_Toc227070418)	16

Supplementary Table S3	17

[Supplementary Table S4	20](#_Toc227070420)

[Supplementary Table S5	2](#_Toc227070421)0

[Supplementary Table S6	22](#_Toc227070422)

[Supplementary Table S7	22](#_Toc227070423)

[Supplementary Table S8	23](#_Toc227070424)

[Supplementary Table S9	23](#_Toc227070425)

[Supplementary Table S10	23](#_Toc227070426)

[Supplementary Table S11	24](#_Toc227070427)

[Supplementary Table S12	24](#_Toc227070428)

[Supplementary Table S13	24](#_Toc227070429)

[Supplementary Table S14	28](#_Toc227070430)

[Supplementary Table S15	28](#_Toc227070431)

[Supplementary Table S16	29](#_Toc227070432)

[Supplementary Table S17	29](#_Toc227070433)

[Supplementary Table S18	30](#_Toc227070434)

[Supplementary Table S19	31](#_Toc227070435)

[Supplementary Table S20	32](#_Toc227070436)

[Supplementary Table S21	32](#_Toc227070437)

[Supplementary Table S22	3](#_Toc227070438)1

[Supplementary Table S23	3](#_Toc227070439)1

[Supplementary Table S24	33](#_Toc227070439)

[Supplementary Table S25	33](#_Toc227070439)

[Supplementary Table S26	37](#_Toc227070439)

[III Supplementary Figures	38](#_Toc227070440)

[Supplementary Figure S1	38](#_Toc227070441)

[Supplementary Figure S2	39](#_Toc227070442)

[Supplementary Figure S3	40](#_Toc227070443)

[Supplementary Figure S4	41](#_Toc227070444)

[Supplementary Figure S5	45](#_Toc227070444)

[Supplementary Figure S6	48](#_Toc227070445)

[IV Supplementary Results](#_Toc227070446)	49

[Supplementary Results S1	49](#_Toc227070447)

[Supplementary Results S2	5](#_Toc227070448)0

[Supplementary Results S3	50](#_Toc227070449)

[Supplementary Results S4	5](#_Toc227070449)1

[Supplementary Results S5	5](#_Toc227070449)3

[References	5](#_Toc227070450)4

# Supplementary Materials

I Supplementary Methods

Supplementary Method S1. Inclusion and exclusion criteria

**S1.1 Dataset 1 (Xuanwu Hospital, Capital Medical University; the Chinese Neuroimmunological Diseases [NIDBase] cohort study)**

**MRI cohort**

The MS diagnosis was confirmed by board-certified neurologists according to the 2017 revised McDonald criteria^1^.

Inclusion criteria for participants with MS were: (1) age between 18 and 65 years; (2) availability of high-quality 3D T1-weighted and 3D T2-FLAIR images acquired using a standardised MRI protocol on a 3.0 T scanner; (3) completion of clinical assessments including the Expanded Disability Status Scale (EDSS) and Symbol Digit Modalities Test (SDMT) within one week of MRI acquisition; and (4) ability to provide informed consent and comply with the study protocol. Healthy controls (HC) were age-, sex-, and education-matched individuals with no history of neurological or psychiatric disorders and normal MRI findings. Sex was self-reported by participants at enrolment and recorded as female or male; gender identity was not collected. Sex was considered in the study design through matching of healthy controls and was included as a prespecified covariate in the relevant analyses. Exclusion criteria for both groups included: (1) history of other major neurological (e.g., stroke, epilepsy) or systemic disorders that may affect brain structure; (2) poor image quality or severe motion artefacts; (3) current or past substance abuse or alcohol dependence; and (4) use of steroids within one month before MRI scanning.

**EEG cohort**

A subset of participants from the MRI cohort underwent high-density resting-state EEG recordings. Inclusion criteria were: (1) meeting the MRI inclusion criteria and consenting to EEG acquisition; (2) successful completion of the high-density resting-state EEG protocol using a 256-channel HydroCel Geodesic Sensor Net operated with Net Station 5 software (Electrical Geodesics, Inc. [EGI], Eugene, OR, USA; sampling rate = 1000 Hz), with analysable eyes-closed resting-state EEG data available for preprocessing and network analysis; (3) no relapse or corticosteroid use within one month prior to EEG acquisition. Healthy controls were recruited under identical conditions and showed no neurological or psychiatric disorders. Exclusion criteria for both groups included: (1) EEG data quality failure due to artefacts or technical issues; (2) failure to remain awake or still during recording; and (3) abnormal EEG findings suggestive of comorbid neurological conditions.

**S1.2 Dataset 2 (UK Biobank imaging** **cohort)**

Participants with MS were selected from the UK Biobank imaging dataset based on the following criteria: (1) confirmed diagnosis of MS (ICD-10: G35); (2) age between 20 and 70 years at the time of MRI; (3) availability of 3D T1-weighted MRI that passed the UK Biobank quality-control pipeline. In Dataset 2, sex was obtained from the demographic data provided by the UK Biobank and coded as female or male; gender identity was not available in the dataset used for this analysis. Exclusion criteria included: (1) history of other neurological, psychiatric, or systemic disorders that might affect brain structure or function; (2) incomplete imaging or clinical data; (3) failed quality control or visible artefacts on MRI; and (4) structural abnormalities unrelated to MS (e.g., tumour, haemorrhage, or major malformation). Ethical approval and data access permissions for UK Biobank data were granted under application number 917687177. Healthy controls in Dataset 2 were selected from the same database and required to have no diagnosis of MS, available high-quality 3D T1-weighted MRI, and complete demographic data. Individuals with other major brain lesions or incomplete data were excluded.

Supplementary Method S2. MRI acquisition and analysis

# S2.1 MRI scanning parameters in Dataset 1

All MRI scans were acquired in Beijing Puhua International Hospital on a 3.0 T scanner (GE Discovery MR750, GE Healthcare, Milwaukee, WI, USA) equipped with an 8-channel head coil. High-resolution 3D T1-weighted images were acquired using a BRAVO sequence (repetition time [TR] = 7.8 ms, echo time [TE] = 2.9 ms, inversion time [TI] = 743 ms, flip angle = 12°, field of view = 240 × 240 mm², voxel size = 1 × 1 × 1 mm³, 164 sagittal slices) for morphometric and structural network analyses. A 3D T2-weighted fluid-attenuated inversion recovery (3D T2 FLAIR CUBE) sequence was obtained to visualise demyelinating lesions (TR = 6300 ms, TE = 105 ms, TI = 1775 ms, echo train length = 180, flip angle = 111°, field of view = 256 × 243 mm², voxel size = 1.0 × 1.0 × 1.4 mm³, 232 sagittal slices). All scans were inspected for motion artefacts and image quality before further processing.

# S2.2 MRI scanning parameters in Dataset 2

Structural MRI data were obtained from the UK Biobank imaging protocol. All scans were acquired using a 3T Siemens Skyra MRI scanner with a 32-channel head coil. High-resolution T1-weighted structural images were acquired using a 3D MPRAGE sequence with the following parameters: repetition time (TR) = 2000 ms, echo time (TE) = 2.01 ms, inversion time (TI) = 880 ms, flip angle = 8°, field of view (FOV) = 208 × 256 × 256 mm³, voxel size = 1 mm isotropic. Detailed acquisition parameters can be found in the UK Biobank protocol documentation (Alfaro-Almagro et al., 2018).^1^ All imaging data were obtained under UK Biobank Application ID [917687177] and processed in accordance with the UK Biobank imaging pipeline.

# S2.3 MRI processing

Image data were preprocessed using a unified pipeline based on the SPM toolbox (http://rfmri.org/SPM). For Dataset 1, 3D T2-FLAIR images were first co-registered to the corresponding 3D T1-weighted images and bias-corrected. White matter lesions were then segmented using the SPM12-based Lesion Segmentation Toolbox (LST),^2^ rather than LST-AI, and lesion-filled T1-weighted images were generated for subsequent morphometric analysis. LST-derived lesion masks and lesion-filled T1-weighted images were visually inspected before downstream CAT12 processing to assess lesion coverage, local tissue-intensity continuity, and obvious filling-related artefacts. No routine manual editing was applied to the LST-derived lesion masks.

For Dataset 2, because 3D T2-FLAIR images comparable to those used in Dataset 1 were not available in the UK Biobank dataset, the FLAIR-based LST lesion segmentation pipeline applied to Dataset 1 could not be implemented. To minimise the potential influence of visible white matter lesions on tissue segmentation and grey matter quantification, T1-visible white matter lesions in participants with MS were manually delineated in native T1-weighted space using ITK-SNAP, and binary lesion masks were generated. All lesion masks were visually inspected and manually corrected when necessary to ensure appropriate lesion coverage. The lesion masks were then used as input to the FSL lesion_filling tool to obtain lesion-filled T1-weighted images before CAT12/SPM12 preprocessing.^3^

The quality of the lesion-filled T1-weighted images was visually checked, with particular attention to the adequacy of lesion filling, the continuity of local tissue intensity, and the absence of obvious filling-related artefacts. For participants with MS, lesion-filled T1-weighted images were used for subsequent CAT12/SPM12 preprocessing, whereas original T1-weighted images were used for healthy controls and processed using the same pipeline. Given the absence of 3D T2-FLAIR images in Dataset 2, lesion delineation was limited to T1-visible lesions and may have underestimated small lesions, cortical/juxtacortical lesions, or lesions without clear T1 contrast. Therefore, Dataset 2 was primarily used for external validation, and its findings were interpreted with consideration of sequence-related differences from Dataset 1.

For both datasets, lesion-filled T1-weighted images were subsequently segmented into grey matter (GM), white matter (WM), and cerebrospinal fluid (CSF) using CAT12/SPM12. The segmented GM maps in native space were spatially normalised to Montreal Neurological Institute (MNI) space using diffeomorphic anatomical registration through exponentiated Lie algebra (DARTEL), followed by modulation to preserve regional volumetric information.^4^ The modulated GM maps were then smoothed with an 8-mm full width at half maximum (FWHM) Gaussian kernel.^5, 6^ The resulting lesion-filled, normalised, and smoothed GM maps were used for the subsequent analyses of regional GM properties and individualised structural covariance network (iSCN) construction.

Supplementary Method S3. Structural network construction

**S3.1 iSCN construction and graph-theoretical analysis**

Brain complex networks consist of nodes and edges. In our study, the nodes of iSCN were defined as 170 cortical and subcortical regions based on the parcellation of the AAL3 atlas.^7^ The inter-regional structural covariance (i.e. structural connectivity) represented the edges, calculated as the similarities of grey matter volume (GMV) probability distribution between each pair of regions. Specifically, kernel density estimation (KDE) was first employed to estimate the probability density function (PDF) to reflect the GMV distribution in each region. In this process, the Gaussian kernel was used by default, and a conservative number of sampling points was set to 512, as suggested by previous studies. ^8, 9^ The resulting PDFs were normalised before KLD/JSD estimation, so the similarity measure primarily reflected distributional shape rather than the absolute number of voxels in each ROI. Nevertheless, ROI size may influence the stability of density estimation, particularly in smaller regions, and this issue is acknowledged as a limitation in the Discussion. Next, we measured the differences between pairwise PDFs of regional GMV using the symmetric Kullback–Leibler divergence (KLD). The interregional KLD values were subsequently transformed to Kullback–Leibler similarity (KLS) values which ranged from 0 to 1 (KLS = *e*^−KLD^). Finally, a symmetric 170 × 170 weighted similarity matrix was established to represent the iSCN for each participant.

**S3.2 Sensitivity analysis using Jensen–Shannon divergence**

To validate the robustness and reliability of the primary findings derived from KLD-based iSCN construction, we performed a complementary sensitivity analysis using Jensen–Shannon divergence (JSD).^10^ As a symmetric, bounded, and more stable divergence metric compared with KLD, JSD avoids the numerical instability and unboundedness issues of KLD when comparing non-overlapping regional GMV probability distributions, thus providing a rigorous validation for network construction. Following the identical analytical framework as KLD calculation, JSD was computed based on the regional GMV PDFs estimated via KDE with a Gaussian kernel. The JSD between two probability distributions P and Q was calculated using the formula:

The JSD-based 170×170 similarity matrix was constructed using the AAL3 atlas parcellation, and the subsequent processing steps were strictly consistent with those applied to KLD-based iSCNs. This sensitivity analysis was conducted to confirm that the observed network topological and connectivity alterations in patients with multiple sclerosis were not dependent on the choice of inter-regional similarity metric.

**S3.3 Graph-theoretical metrics and thresholding**

Global metrics included clustering coefficient (Cp), characteristic path length (Lp), small-worldness (γ, λ, σ), global efficiency (Eg), local efficiency (Eloc), modularity (Q), and assortativity (r), reflecting network segregation, integration, small-world organisation, modular structure, and resilience. Nodal metrics included nodal degree, nodal betweenness, and nodal efficiency. A concise description of all metrics is provided in Supplementary Method S4. To reduce weak and potentially spurious edges, each individual iSCN was thresholded and binarised across a range of network densities. The density-based thresholding strategy retained the K% strongest edges, ensuring the same edge density across participants and facilitating group comparisons. To avoid single-threshold bias, dynamic density thresholds from K = 10% to 40% (1% interval) were applied. The lower and upper bounds were determined according to the criteria that (a) all nodes remained connected and (b) small-worldness was greater than 1.1 (Supplementary Figure S1). To further assess the influence of threshold selection, graph-theoretical analyses were repeated using two additional density ranges, K = 0.20–0.50 and K = 0.30–0.60, defined independently of the small-worldness criterion. These analyses were used to examine whether the main topological findings depended on the primary K = 0.10–0.40 range. In accordance with the revised discovery–validation design, Dataset 1 and Dataset 2 were analysed separately, and no cross-cohort ComBat harmonisation was applied in the primary analyses. Between-group comparisons of topological metrics were performed using general linear models with prespecified covariates as described in the main Methods. Multiple comparisons were controlled using the Benjamini–Hochberg false discovery rate procedure, and corrected P values are reported as *q* values.

Supplementary Method S4. Network topological metrics

The following graph-theoretical metrics were computed on thresholded binary networks. Global metrics included the normalised clustering coefficient (γ), normalised characteristic path length (λ), small-worldness (σ = γ/λ), clustering coefficient (Cp), characteristic path length (Lp), global efficiency (Eg), local efficiency (Eloc), modularity (Q), and assortativity (r). Nodal metrics included degree centrality (Dc), betweenness centrality (Bc), and nodal efficiency (Ne). Metric definitions followed standard graph-theoretical formulations for binary networks.^11^ All graph-theoretical metrics were defined according to Bullmore and Sporns (2009).^12^

1. Global properties

Cp reflects the extent of local clustering in the network. Lp reflects the average shortest path length between nodes. γ and λ represent the normalised clustering coefficient and normalised characteristic path length relative to matched random networks, and σ was calculated as γ/λ. Eg reflects the capacity for global parallel information transfer, whereas Eloc reflects local information transfer efficiency. Q quantifies the degree of

modular organisation, and assortativity reflects the tendency of nodes to connect with other nodes of similar degree.

2. Nodal properties

Degree centrality reflects the number of edges connected to a node. Betweenness centrality reflects the proportion of shortest paths passing through a node. Nodal efficiency reflects the efficiency of communication between a given node and all other nodes in the network. These nodal metrics characterise the relative importance of individual nodes for information transfer and network integration

Supplementary Method S5. **Analysis of connectivity patterns of iSCN**

Between-group differences in structural covariance connectivity were investigated using network-based statistics (NBS), which identifies connected components showing altered connectivity between groups. First, all edges in the iSCN were tested for between-group differences, and suprathreshold edges were defined using an initial two-tailed p < 0.001. Breadth-first search was then used to identify connected components among suprathreshold edges. Component-level significance was assessed using non-parametric permutation testing with 10,000 randomisations, and the size of the maximal connected component was recorded for each permutation. Observed components were considered significant if their sizes exceeded the 95th percentile of the permutation-derived maximal component-size distribution, corresponding to component-level family-wise error-corrected significance at p_FWE_ < 0.05. Nodes within each significant component were assigned to seven canonical intrinsic functional networks from the Yeo atlas: default mode network (DMN), ventral attention network (VAN), visual network (VN), dorsal attention network (DAN), somatomotor network (SMN), frontoparietal network (FPN), and limbic network (LN). The Yeo atlas provides a standard systems-level framework for summarising large-scale connectivity patterns, although structural covariance and intrinsic functional network boundaries may not be fully aligned; this limitation is discussed in the manuscript.

To summarise the large-scale distribution of significant NBS-derived altered edges, the number of altered edges within each within-network or between-network pair was counted for each comparison and direction of effect separately (group 1 > group 2 and group 1 < group 2). For visualisation, relative connection weight was defined as the ratio of the observed number of altered edges to the maximum possible number of edges for that network pair.

To evaluate whether altered edges were over-represented in a given network pair, a one-sided hypergeometric test was performed for each network pair, using the total number of significant altered edges in that direction as the reference set and the total number of possible edges in that network pair as the background. Because the functional-network matrix is symmetric, statistical testing was restricted to the 28 unique network pairs in the lower triangle, including the 7 within-network pairs and the 21 between-network pairs. *P* values from these network-pair tests were corrected using the Benjamini–Hochberg false discovery rate procedure, separately for increased and decreased edges, and are reported as *q* values.

Supplementary Method S6. Support vector machine

We separately used network topological features and connectivity features to train support vector machine (SVM) classifiers.^13^ The SVM model was implemented using the MATLAB R2021b platform. The primary aim of these analyses was to compare the cross-validated discriminative performance of topology- and connectivity-based feature representations, rather than to derive a single model for clinical implementation. Because feature scaling, feature selection, and hyperparameter tuning were repeated within each training fold, the fitted model parameters and, for connectivity-based models, the selected features could vary across folds. Consequently, no single locked model or final trained model object was generated. Linear SVM was chosen for its effectiveness in high-dimensional neuroimaging data and good interpretability.^14^ For each classification task, stratified 10-fold cross-validation was applied. All preprocessing steps, including feature scaling, feature selection, and hyperparameter tuning, were performed within the training data of each fold only, and the resulting parameters were then applied to the corresponding held-out fold to avoid information leakage. For MRI-based topology models, the feature set comprised 8 global metrics, excluding assortativity, and 3 nodal centrality measures across 170 regions, resulting in 518 features per participant. For MRI-based connectivity models, the feature set comprised 14,365 unique edges [170 × (170 − 1) / 2]. For EEG-based classification, features were modelled separately for each frequency band. For EEG-based topology models, the feature set comprised 8 global metrics, excluding assortativity, and 3 nodal centrality measures across 104 regions, resulting in 320 features per participant. For EEG-based connectivity models, the feature set comprised 5,356 unique edges [104 × (104 − 1) / 2]. Topology-based models were trained using the full topological feature set. For connectivity-based models, univariate feature selection was performed within each training fold, retaining edges showing significant between-group differences at *P* < 0.001 to reduce dimensionality and mitigate overfitting. The hyperparameter C was optimised via grid search (10^-3^ to 10^3^) with nested 10-fold cross-validation. We designed six classification tasks: four Dataset 1 MRI-based tasks (MS vs HC, high vs low disability, CI vs CP, NEDA vs EDA), one Dataset 2 MRI-based task (MS vs HC), and one EEG-based task (MS vs HC). For EEG-based MS-versus-HC classification, models were run separately for each frequency band. The Dataset 2 classification task was developed and internally evaluated separately, and no fitted model parameters were transferred from Dataset 1. Model performance was evaluated using accuracy, sensitivity, specificity, balanced accuracy, and the area under the receiver operating characteristic curve (AUC), averaged across the outer folds. Ninety-five percent confidence intervals are reported in Supplementary Table S19. Class labels were assigned using the default decision threshold of the linear SVM classifier, corresponding to a decision score of 0. AUC was calculated across the full range of decision-score thresholds. To assess whether classification performance exceeded chance level, permutation testing was performed for each classification task by repeating the full nested cross-validation pipeline 1000 times with shuffled class labels. Empirical *P* values were calculated from the resulting null distributions. To summarise model importance, we calculated the mean absolute weight of each feature across folds. The top ten contributing regions were reported because regional contributions in brain models typically follow a long-tailed distribution, with a relatively small number of regions accounting for most of the predictive signal, whereas lower-ranked regions contribute comparatively little and are less robust to interpret. For topology-based models, regional importance was obtained by averaging the absolute weights of the three nodal centrality features for each region; global topological features were not included in regional mapping. For connectivity-based models, regional importance was obtained by averaging the absolute weights of all incident connections for each region.

Supplementary Method S7. EEG acquisition and analysis

**S7.1 EEG acquisition and preprocessing**

Resting-state EEG was recorded using a 256-channel high-density EEG system equipped with a HydroCel Geodesic Sensor Net and operated with Net Station 5 software (Electrical Geodesics, Inc. [EGI], Eugene, OR, USA), with a sampling rate of 1000 Hz for 30 minutes. All recordings were conducted in sound-attenuated, electromagnetically shielded rooms, with data collected using the same equipment operated by the same research team across multiple centres. Each resting-state EEG session followed a structured paradigm that alternated between eye-open and eye-closed conditions to minimise state-related confounding effects. Specifically, each session consisted of four consecutive stages: 5 minutes of eyes-open resting state, 5 minutes of eyes-closed resting state, another 5 minutes of eyes-open resting state, and a final 15-minute eyes-closed resting state. Participants were instructed to remain relaxed, minimise movement, and refrain from engaging in active mental tasks during all stages of the session. This design aimed to capture spontaneous cortical activity under different vigilance states and ensure sufficient data length for reliable analysis of resting-state neural dynamics. Preprocessing was standardised across all participants using data collected during the eyes-closed state. The preprocessing pipeline was applied using the EEGLAB toolbox (http://sccn.ucsd.edu/eeglab/index.php), and included the following steps: 1) band-pass filtering (0.1-104 Hz); 2) artefact removal: problematic channels were interpolated using spherical splines and re-referenced to the average; 3) Independent Component Analysis (ICA) was applied to remove ocular, muscular, and cardiac artefacts; 4) Data were segmented into 2-second epochs, and segments with amplitudes >100 µV or extreme voltage fluctuations were excluded; 5) Final signals were projected to source space using a linearly constrained minimum variance (LCMV) beamformer. Source localisation to cortical and subcortical brain areas was accomplished using the Brainstorm toolbox (http://neuroimage.usc.edu/brainstorm). Time series data were then extracted from 104 brain regions defined by the Automated Anatomical Labeling (AAL3) atlas. A total of 13,562 unconstrained dipoles were defined across the cortical surface. For regional analysis, dipole activity was averaged within the 104 AAL-defined brain regions.

**S7.2 EEG network construction and graph-theoretical analysis**

Global and nodal topological metrics of EEG functional networks were computed using the GRETNA toolbox on the MATLAB platform, following procedures analogous to those applied in the MRI-based iSCN analysis. For each participant, nine canonical frequency bands—delta (1–4 Hz), theta (4–8 Hz), low alpha (8–11.5 Hz), high alpha (11.5–13 Hz), low beta (13–17 Hz), middle beta (17–21 Hz), high beta (21–28 Hz), low gamma (31–47 Hz), and high gamma (53–97 Hz)—were extracted via complex Morlet wavelet transform. Power spectral density (PSD) was estimated in the source space, and Pearson correlation coefficients were computed between all pairs of 104 regions, resulting in 5,356 unique connections (104 × 103 / 2) per frequency band. To remove weak and potentially spurious connections, each functional network was thresholded and binarised

across a range of network densities. The density-based thresholding strategy retained the K% strongest connections, ensuring identical edge numbers across participants to facilitate between-group comparisons. To avoid single-threshold bias, dynamic density thresholds ranging from K = 10% to 40% (interval = 1%) were applied, satisfying the criteria that (a) all nodes remained connected and (b) network small-worldness exceeded 1.1 (Supplementary Figure S1). Global metrics included clustering coefficient (Cp), characteristic path length (Lp), small-worldness (γ, λ, σ), global efficiency (Eg), local efficiency (Eloc), modularity (Q), and assortativity (r). Nodal metrics included degree centrality, betweenness centrality, and nodal efficiency (Supplementary Methods S4).

**S7.3 Analysis of connectivity patterns of EEG functional networks**

Between-group differences in EEG functional connectivity were analysed using NBS, following the same analytical framework as applied to the MRI-based iSCN analysis. For each participant, source-level functional connectivity matrices were constructed by computing pairwise Pearson correlation coefficients of power spectral density (PSD) values across 104 cortical and subcortical regions, yielding 5,356 unique connections per frequency band. NBS analysis was then conducted to identify subnetworks exhibiting significantly altered connectivity between MS and healthy controls, as well as among clinically defined subgroups (e.g., disability, cognitive, and disease activity stratification). Statistical thresholds (two-tailed *p* < 0.001; corrected *p* < 0.05, 10,000 permutations) and component identification procedures were identical to those used in the MRI analyses. Significant EEG subnetworks were further mapped onto seven canonical functional systems (DMN, DAN, SMN, VAN, FPN, VN, LN) based on the Yeo parcellation, enabling characterisation of frequency-specific inter- and intra-network connectivity patterns.

For each comparison and each frequency band showing significant NBS results, significant altered edges were mapped onto the same seven canonical functional networks. The number of altered edges within each within-network or between-network pair was counted separately for the two directions of effect. Relative connection weight was defined as the ratio of the observed number of altered edges to the maximum possible number of edges for that network pair. Statistical significance at the network-pair level was tested using one-sided hypergeometric tests, followed by Benjamini–Hochberg false discovery rate correction across the 28 unique network pairs of the symmetric 7 × 7 matrix. Corrected P values are reported as *q* values.

II Supplementary Tables

Supplementary Table S1. **Demographic and clinical characteristics of MRI across prespecified subgroup analyses.**

| **Supplementary Table S1.1. High disability (EDSS ≥ 4) versus low disability (EDSS ≤ 3.5)** | | | |
| --- | --- | --- | --- |
| **Variables** | **EDSS ≤ 3.5 (n = 130)** | **EDSS ≥ 4 (n = 33)** | ***p* value** |
| Age at MRI, years, median (IQR) | 34.0 (27.0–40.0) | 35.0 (28.0–49.0) | 0.169^(a)^ |
| Sex, n (%) |  |  | 0.068^(b)^ |
| Female | 86 (66.15) | 28 (84.85) |  |
| Male | 44 (33.85) | 5 (15.15) |  |
| BMI, median (IQR) | 22.43 (20.70–24.91) | 23.73 (21.58–26.66) | 0.118^(a)^ |
| Disease duration, years, median (IQR) | 2.50 (0.5–8.0) | 7.75 (1.8–12.0) | 0.002^(a)^ |
| EDSS at baseline, median (IQR) | 1.50 (0.00–2.12) | 5.50 (4.00–6.00) | <0.001^(a)^ |
| SDMT at baseline, median (IQR) | 51.00 (45.00–60.00) | 47.00 (40.25–56.00) | 0.056**^(a)^** |
| Education years, median (IQR) | 16.0 (12.0–16.0) | 15.0 (12.0–16.0) | 0.068**^(a)^** |
| DMT at baseline, n (%) |  |  | 0.212^(c)^ |
| no | 36 (27.69) | 13(39.39) |  |
| moderate^(e)^ | 42 (32.31) | 12(36.36) |  |
| high^(f)^ | 52 (40.00) | 8 (24.24) |  |
| TIV, cm^3^, mean ± SD | 1416.82 ± 174.11 | 1334.65 ± 181.36 | 0.017^(d)^ |
| **Supplementary Table S1.2. Cognitive impairment (CI) versus cognitively preserved (CP)** | | | |
| **Variables** | **CP (n = 119)** | **CI (n = 44)** | ***P* value** |
| Age at MRI, years, median (IQR) | 32.0 (27.0–38.3) | 36.0 (31.2–50.0) | <0.001^(a)^ |
| Sex, n (%) |  |  | 0.987(b) |
| Female | 84 (70.59) | 31 (70.45) |  |
| Male | 35 (29.41) | 13 (29.55) |  |
| BMI, median (IQR) | 23.57 (21.29–26.08) | 23.12 (20.02–25.98) | 0.422^(a)^ |
| Disease duration, years, median (IQR) | 2.6 (0.5- 8.0) | 6.8 (0.62–10.7) | 0.021^(a)^ |
| EDSS at baseline, median (IQR) | 1.00 (0.00–2.00) | 2.50 (2.00–4.75) | <0.001^(a)^ |
| SDMT at baseline, median (IQR) | 54.00 (50.00–64.25) | 34.50 (29.25–40.00) | <0.001^(a)^ |
| Education years, median (IQR) | 16.0 (12.0–16.0) | 15.0 (12.0–16.0) | <0.001^(a)^ |
| DMT at baseline, n (%) |  |  | 0.265^(c)^ |
| no | 35 (29.41) | 14(31.82) |  |
| moderate^(e)^ | 36 (30.25) | 18 (40.91) |  |
| high^(f)^ | 48 (40.34) | 12 (27.27) |  |
| TIV, cm^3^, mean ± SD | 1411.55 ± 169.06 | 1376.42 ± 182.32 | 0.340^(d)^ |
| **Supplementary Table S1.3.evidence of disease activity (EDA) versus no evidence of disease activity (NEDA)** | | | |
| **Variables** | **NEDA (n = 94)** | **EDA (n = 69)** | ***p* value** |
| Age at MRI, years, median (IQR) | 34.0 (27.0–39.0) | 35.00 (27.0–45.7) | 0.466^(a)^ |
| Sex, n (%) |  |  | 0.070^(b)^ |
| Female | 60 (63.82) | 54 (78.26) |  |
| Male | 34(36.18) | 15 (21.74) |  |
| BMI, median (IQR) | 22.55 (21.08–25.34) | 23.01 (20.70–25.43) | 0.748^(a)^ |
| Disease duration, years, median(IQR) | 1.5 (0.2–5.5) | 5.0 (1.5–9.3) | <0.001^(a)^ |
| EDSS at baseline, median (IQR) | 1.50 (0.00–2.00) | 2.00 (1.00–3.85) | 0.031^(a)^ |
| SDMT at baseline, median (IQR) | 51.00 (45.0–60.0) | 50.00 (41.25–55.50) | 0.108^(a)^ |
| Education years, years, median (IQR) | 16.0 (12.0–16.0) | 16.0 (12.0–16.0) | 0.856^(a)^ |
| DMT at baseline, n (%) |  |  | <0.001^(c)^ |
| no | 22 (23.41) | 27 (39.13) |  |
| moderate^(e)^ | 25(26.59) | 29(42.03) |  |
| high^(f)^ | 47 (50.00) | 13(18.84) |  |
| TIV, cm^3^, mean ± SD | 1429.20 ± 173.02 | 1362.99 ± 178.43 | 0.018^(d)^ |
| Follow-up duration for NEDA/EDA classification, months, median (IQR) | 12.0 (11.0–14.0) | 13.0 (11.2–14.0) | 0.850^(a)^ |
| **EDA-defining events, n (EDA group only; non-mutually exclusive)** |  |  |  |
| Relapses, n |  | 16 | *n.a* |
| MRI activity, n |  | 12 | *n.a* |
| EDSS progression over follow-up time, n |  | 46 | *n.a* |
| **Supplementary Table S1.4. Demographic and clinical characteristics of the longitudinal NEDA subgroup** | | | |
| **Variables** | **Longitudinal NEDA subgroup (n = 33)** | | |
| Age at MRI, years, median (IQR), | 34.0 (27.0–40.0) | | |
| Sex, n (%) |  | | |
| Female | 19 (57.6) | | |
| Male | 14 (42.4) | | |
| BMI, median (IQR) | 23.62 (20.31–28.65) | | |
| Disease duration, years, median (IQR) | 3.0 (0.5–11.0) | | |
| EDSS at baseline, median (IQR) | 1.50 (0.00–2.50) | | |
| SDMT at baseline, median (IQR) | 54.00 (45.00–59.00) | | |
| Education years, median (IQR) | 15.0 (12.0–16.0) | | |
| DMT at baseline, n (%) |  | | |
| no | 6 (18.18) | | |
| moderate^(e)^ | 15 (45.46) | | |
| high^(f)^ | 12 (36.36) | | |
| TIV, cm^3^, mean ± SD | 1439.45 ± 173.82 | | |
| Interval between baseline and follow-up MRI scans, months, median (IQR) | 11.4 (11.0–22.0) | | |

**Abbreviations: BMI, body mass index; CI, cognitive impairment; CP, cognitively preserved; DMT, disease-modifying therapy; EDA, evidence of disease activity; EDSS, Expanded Disability Status Scale; IQR, interquartile range; MRI, magnetic resonance imaging; NEDA, no evidence of disease activity; SD, standard deviation; SDMT, Symbol Digit Modalities Test; TIV, total intracranial volume.**

**Notes: For Table 1.3: EDA-defining events were not mutually exclusive; therefore, the sum of individual event counts may exceed the total number of patients in the EDA group. The follow-up duration in Table 1.3 refers to the clinical observation period used for NEDA/EDA classification, during which relapse, MRI activity, and EDSS progression were assessed. The interval between baseline and follow-up MRI scans in Table 1.4 refers to the actual scan-to-scan interval in the longitudinal NEDA subgroup. This variable was used for longitudinal imaging analysis and is conceptually different from the follow-up duration used for NEDA/EDA classification in Table 1.3.**

**Data are presented as median (IQR), mean ± SD, or n (%), as appropriate.
(a) *p* value derived from the Mann–Whitney U test.
(b) *p* value derived from Pearson’s chi-square test.
(c) *p* value derived from Pearson’s chi-square test or Fisher’s exact test, as appropriate.
(d) *p* value derived from the independent-samples t test.**

**(e) Moderate-efficacy DMTs included glatiramer acetate, interferon-beta, teriflunomide, dimethyl fumarate, and azathioprine.
(f) High-efficacy DMTs included fingolimod, natalizumab, rituximab, and mitoxantrone.**

Supplementary Table S2. Top 10% of significant regional topological differences ranked by absolute effect size in patients with MS versus healthy controls in Dataset 1, adjusted for age, sex, BMI, and TIV.

| **Regions** | **Metrics** | **Cohen’s *d*** | ***q value*** |
| --- | --- | --- | --- |
| **MS > HC** |  |  |  |
| lPreCG,Left Precentral gyrus | nodal betweenness  nodal degree | 0.90 | **<0.001** |
| rPreCG, Right Precentral gyrus | nodal efficiency | 0.87 | **<0.001** |
| lIFGorb,Left IFG pars orbitalis | nodal efficiency | 0.27 | **0.002** |
| lSFGmedial, Left Superior frontal gyrus-medial | nodal betweenness | 0.75 | **<0.001** |
| lAMYG, Left Amygdala | nodal betweenness | 0.72 | **<0.001** |
| lPAL,Left Lenticular nucleus-Pallidum | nodal betweenness | 0.75 | **<0.001** |
| rCER4_5, Right Lobule IV-V of cerebellar hemisphere | nodal degree | 0.80 | **<0.001** |
| lCER6, Left Lobule VI of cerebellar hemisphere | nodal degree | 0.20 | **0.03** |
| rCER6, Right Lobule VI of cerebellar hemisphere | nodal degree | 0.21 | **0.02** |
| rCER10, Right Lobule X of cerebellar hemisphere | nodal betweenness | 0.80 | **<0.001** |
| ltMGN, Left Medial Geniculate | nodal betweenness | 0.80 | **<0.001** |
| ltVA, Left Ventral anterior | nodal efficiency | 0.27 | **0.002** |
|  | nodal degree | 0.26 | **0.01** |
| ltPuM, Left Pulvinar medial | nodal efficiency | 0.80 | **<0.001** |
| rtPuL, Right Pulvinar lateral | nodal degree | 0.75 | **<0.001** |
| ltMDm, Left Mediodorsal medial magnocellular | nodal degree | 0.80 | **<0.001** |
| lRedN, Left Red nucleus | nodal betweenness | 0.79 | **<0.001** |
| rRedN, Right Red nucleus | nodal betweenness | 0.80 | **<0.001** |
| **MS < HC** |  |  |  |
| rOFClat, Right Lateral orbital gyrus | nodal degree | -0.80 | **<0.001** |
| rIFGorb, Right IFG pars orbitalis | nodal efficiency | -0.39 | **0.03** |
| rSMA, Right Supplementary motor area | nodal betweenness | -0.40 | **0.02** |
|  | nodal efficiency | -0.23 | **0.04** |
| lOLF, Left Olfactory cortex | nodal efficiency | -0.49 | **0.01** |
| rOLF, Right Olfactory cortex | nodal degree | -0.37 | **0.02** |
| rACC, Right Anterior cingulate & paracingulate gyri | nodal degree | -0.40 | **<0.001** |
| lOFClat,Left Lateral orbital gyrus | nodal efficiency | -0.80 | **<0.001** |
|  | nodal degree | -0.80 | **<0.001** |
| lPCC, Left Posterior cingulate gyrus | nodal efficiency | -0.80 | **<0.001** |
| rPCC, Right Posterior cingulate gyrus | nodal degree | -0.80 | **<0.001** |
| lLING, Left Lingual gyrus | nodal degree | -0.80 | **<0.001** |
|  | nodal efficiency | -0.80 | **<0.001** |
| lOFCpost, Left Posterior orbital gyrus | nodal efficiency | -0.80 | **<0.001** |

Only regional topological differences surviving FDR correction (q < 0.05) were considered. Among these significant findings, the top 10% with the largest absolute Cohen’s d values were presented.

**Abbreviations:** MS: multiple sclerosis; HC: healthy controls.

# **Supplementary Table S3. Regional topological abnormalities in UK Biobank participants with MS versus healthy controls, adjusted for sex, age, BMI, and TIV.**

| **Regions** | **Metrics** | **Cohen’s d** | ***q value*** |
| --- | --- | --- | --- |
| **MS > HC** |  |  |  |
| rANG, Right Angular gyrus | nodal betweenness | 0.602 | 0.002 |
| rSTG, Right Superior temporal gyrus | nodal betweenness | 0.555 | 0.008 |
| ltMDm, Left Mediodorsal medial magnocellular | nodal betweenness | 0.768 | <0.001 |
| rtMDm, Right Mediodorsal medial magnocellular | nodal betweenness | 0.748 | <0.001 |
| ltMDl, Left Mediodorsal lateral parvocellular | nodal betweenness | 0.789 | <0.001 |
| rtMDl, Right Mediodorsal lateral parvocellular | nodal betweenness | 0.689 | <0.001 |
| rtPuA, Right Pulvinar anterior | nodal betweenness | 0.675 | 0.001 |
| ltPuM, Left Pulvinar medial | nodal betweenness | 0.533 | 0.015 |
| rPreCG, Right Precentral gyrus | nodal efficiency | 0.575 | 0.004 |
| lSFG, Left Superior frontal gyrus-dorsolateral | nodal efficiency | 0.588 | <0.001 |
| rACC, Right Anterior cingulate & paracingulate gyri | nodal efficiency | 0.749 | <0.001 |
| rPCC, Right Posterior cingulate gyrus | nodal efficiency | 0.535 | 0.011 |
| lPAL, Left Lenticular nucleus-Pallidum | nodal efficiency | 0.535 | 0.014 |
| rPAL, Right Lenticular nucleus-Pallidum | nodal efficiency | 0.708 | <0.001 |
| lTHA, Left Thalamus | nodal efficiency | 0.575 | 0.004 |
| ltMDm, Left Mediodorsal medial magnocellular | nodal efficiency | 0.723 | <0.001 |
| rtMDm, Right Mediodorsal medial magnocellular | nodal efficiency | 0.776 | <0.001 |
| ltMDl, Left Mediodorsal lateral parvocellular | nodal efficiency | 0.816 | <0.001 |
| rtMDl, Right Mediodorsal lateral parvocellular | nodal efficiency | 0.735 | <0.001 |
| ltPuM, Left Pulvinar medial | nodal efficiency | 0.88 | <0.001 |
| rtPuM, Right Pulvinar medial | nodal efficiency | 0.86 | <0.001 |
| ltPuL, Left Pulvinar lateral | nodal efficiency | 0.84 | <0.001 |
| rtPuL, Right Pulvinar lateral | nodal efficiency | 0.517 | 0.024 |
| rPCC, Right Posterior cingulate gyrus | nodal degree | 0.578 | 0.004 |
| rtAV, Right Thalamus-Anteroventral Nucleus | nodal degree | 0.714 | <0.001 |
| rtLP, Right Lateral posterior | nodal degree | 0.560 | <0.001 |
| ltVA, Left Ventral anterior | nodal degree | 0.528 | <0.001 |
| ltIL, Left Intralaminar | nodal degree | 0.544 | 0.011 |
| rtIL, Right Intralaminar | nodal degree | 0.495 | 0.045 |
| ltRe, Left Reuniens | nodal degree | 0.670 | <0.001 |
| ltMDm, Left Mediodorsal medial magnocellular | nodal degree | 0.573 | 0.04 |
| rtMDm, Right Mediodorsal medial magnocellular | nodal degree | 0.608 | 0.001 |
| ltMDl, Left Mediodorsal lateral parvocellular | nodal degree | 0.641 | <0.001 |
| ltMGN, Left Medial Geniculate | nodal degree | 0.670 | 0.001 |
| ltPuA, Left Pulvinar anterior | nodal degree | 0.666 | <0.001 |
| rtPuA, Right Pulvinar anterior | nodal degree | 0.663 | <0.001 |
| **MS < HC** |  |  |  |
| ltPuI, Left Pulvinar inferior | nodal efficiency | -0.654 | <0.001 |
| rtPuI, Right Pulvinar inferior | nodal efficiency | -0.818 | <0.001 |
| rVTA, Right Ventral tegmental area | nodal efficiency | -0.520 | 0.022 |
| lSNpc, Left Substantia nigra-pars compacta | nodal efficiency | -0.532 | 0.015 |
| lSNpr, Left Substantia nigra-pars reticulata | nodal efficiency | -0.588 | 0.003 |
| lLC, Left Locus coeruleus | nodal efficiency | -0.736 | <0.001 |
| rtAV, Right Thalamus-Anteroventral Nucleus | nodal betweenness | -0.543 | 0.011 |
| ltVPL, Left Ventral posterolateral | nodal betweenness | -0.516 | 0.024 |
| rtVPL, Right Ventral posterolateral | nodal betweenness | -0.757 | <0.001 |
| rtIL, Right Intralaminar | nodal betweenness | -0.575 | 0.004 |
| rSOG, Right Superior occipital gyrus | nodal degree | -0.585 | 0.003 |
| ltPuI, Left Pulvinar inferior | nodal degree | -0.713 | <0.001 |
| rtPuI, Right Pulvinar inferior | nodal degree | -0.697 | <0.001 |
| lSNpc, Left Substantia nigra-pars compacta | nodal degree | -0.502 | 0.037 |
| lLC, Left Locus coeruleus | nodal degree | -0.709 | <0.001 |

**Abbreviations:** MS: multiple sclerosis; HC: healthy controls.

Supplementary Table S4. **Statistical results for global network metrics of MRI-derived iSCNs in Dataset 1, adjusted using comparison-specific covariates as described in the Methods.**

| **Global metrics** | **iSCN (individual-level)** | ***q value*** |
| --- | --- | --- |
| **MS vs HC** |  |  |
| AUC of γ | MS > HC | *q* < 0.001 |
| AUC of λ | MS > HC | *q* < 0.001 |
| AUC of σ | MS > HC | *q* < 0.001 |
| AUC of Cp | MS < HC | *q* < 0.001 |
| AUC of Lp | MS < HC | *q*< 0.001 |
| AUC of Eg | MS > HC | *q* < 0.001 |
| AUC of Eloc | MS < HC | *q* < 0.001 |
| AUC of Q | MS > HC | *q* < 0.001 |
| **CI** vs **CP** |  |  |
| AUC of γ | ns | ns |
| AUC of λ | CI > CP | *q* < 0.001 |
| AUC of σ | ns | ns |
| AUC of Cp | ns | ns |
| AUC of Lp | ns | ns |
| AUC of Eg | ns | ns |
| AUC of Eloc | ns | ns |
| AUC of Q | ns | ns |
| **high vs low disability** |  |  |
| AUC of γ | ns | 0.09 |
| AUC of λ | high > low disability | 0.04 |
| AUC of σ | ns | 0.06 |
| AUC of Cp | high < low disability | 0.04 |
| AUC of Lp | high > low disability | 0.001 |
| AUC of Eg | high >low disability | 0.009 |
| AUC of Eloc | high <low disability | 0.008 |
| AUC of Q | ns | 0.17 |
| **NEDA vs EDA** |  |  |
| AUC of γ | ns | ns |
| AUC of λ | ns | ns |
| AUC of σ | ns | ns |
| AUC of Cp | ns | ns |
| AUC of Lp | ns | ns |
| AUC of Eg | ns | ns |
| AUC of Eloc | ns | ns |
| AUC of Q | ns | ns |

**Notes:** iSCN denotes the individual-level structural covariance network. Results indicate the direction of between-group differences and the corresponding multiple-comparison-corrected *P* values for global topological metrics. Non-significant results are denoted as ns. As described in the Methods, global metrics were corrected using the false discovery rate (FDR) correction. Covariates were prespecified according to each comparison, as detailed in the main Methods. Abbreviations**:** CI: cognitively impaired; CP: cognitively preserved; NEDA: no evidence of disease activity; EDA: evidence of disease activity. MS: multiple sclerosis; HC: healthy control; iSCN: individual-level structural covariance network; AUC, area under the metric–density curve across the predefined network density thresholds; γ: normalised clustering coefficient; λ: normalised characteristic path length; σ: small-worldness; Cp: clustering coefficient; Lp: characteristic path length; Eg: global efficiency; Eloc: local efficiency; Q: modularity; r：assortativity. BMI: body mass index; TIV: total intracranial volume.

Supplementary Table S5. **Comparison of global graph-theoretical metrics of the iSCN between MS and HC groups in Dataset 1, adjusted for sex, age, BMI, and TIV.**

| iSCN global metrics | MS | HC | ***q value*** |
| --- | --- | --- | --- |
| AUC of γ | 0.694 ± 0.256 | 0.487± 0.38 | <0.001^*^ |
| AUC of λ | 0.363 ± 0.005 | 0.344 ± 0.006 | <0.001^*^ |
| AUC of σ | 0.566 ± 0.194 | 0.420 ± 0.342 | <0.001^*^ |
| AUC of Cp | 0.183 ± 0.005 | 0.200 ± 0.040 | <0.001^*^ |
| AUC of Lp | 0.593 ± 0.009 | 0.610 ± 0.011 | <0.001^*^ |
| AUC of Eg | 0.158 ± 0.018 | 0.153 ± 0.002 | <0.001^*^ |
| AUC of Eloc | 0.232 ± 0.003 | 0.239 ± 0.003 | <0.001^*^ |
| AUC of Q | 0.114 ± 0.006 | 0.086 ± 0.008 | <0.001^*^ |
| AUC of r | 0.080 ± 1.712 | 0.124 ± 0.065 | 0.070 |

**Data were presented as mean±standard deviation**

***Statistical significance*:q:* FDR correction (adjusted P < 0.05);**

**Abbreviations:** MS: multiple sclerosis; HC: healthy control; iSCN: individual-level structural covariance network; AUC, area under the metric–density curve across the predefined network density thresholds; γ: normalised clustering coefficient; λ: normalised characteristic path length; σ: small-worldness; Cp: clustering coefficient; Lp: characteristic path length; Eg: global efficiency; Eloc: local efficiency; Q: modularity; r：assortativity; BMI: body mass index; TIV: total intracranial volume.

Supplementary Table S6. **Comparison of global graph-theoretical metrics of the iSCN between MS and HC groups in Dataset 2, adjusted for sex, age, BMI, and TIV.**

| iSCN global metrics | MS | HC | ***q value*** |
| --- | --- | --- | --- |
| AUC of γ | 0.703 ± 0.021 | 0.711 ± 0.197 | 0.006^*^ |
| AUC of λ | 0.364 ± 0.006 | 0.366 ± 0.005 | <0.001^*^ |
| AUC of σ | 0.572 ± 0.018 | 0.576 ± 0.15 | 0.166 |
| AUC of Cp | 0.186 ± 0.006 | 0.191 ± 0.005 | <0.001^*^ |
| AUC of Lp | 0.593 ± 0.124 | 0.599 ± 0.011 | <0.001^*^ |
| AUC of Eg | 0.158 ± 0.002 | 0.157 ± 0.002 | 0.0017^*^ |
| AUC of Eloc | 0.235 ± 0.04 | 0.237 ± 0.03 | <0.001^*^ |
| AUC of Q | 0.118 ± 0.005 | 0.120 ± 0.005 | 0.016^*^ |
| AUC of r | 0.203 ± 0.500 | 0.122 ± 0.480 | 0.216 |

**Data were presented as mean±standard deviation**

***Statistical significance:*q:* FDR correction (adjusted P < 0.05);**

**Abbreviations:** MS: multiple sclerosis; HC: healthy controls; iSCN: individual-level structural covariance network; AUC, area under the metric–density curve across the predefined network density thresholds; γ: normalised clustering coefficient; λ: normalised characteristic path length; σ: small-worldness; Cp: clustering coefficient; Lp: characteristic path length; Eg: global efficiency; Eloc: local efficiency; Q: modularity; r: assortativity; BMI: body mass index; TIV: total intracranial volume.

Supplementary Table S7. Statistical summary of nodal topological findings of MRI-derived iSCNs in Dataset 1, adjusted using comparison-specific covariates as described in the Methods.

| **Nodal Metrics** | **iSC (individual-level) Number of nodal findings** | |
| --- | --- | --- |
| **MS** vs **HC** | **MS > HC** | **MS < HC** |
| nodal betweenness | 41 | 12 |
| nodal degree | 37 | 39 |
| nodal efficiency | 37 | 41 |
| **CI** vs **CP** | **CI > CP** | **CI < CP** |
| nodal betweenness | 0 | 0 |
| nodal degree | 1 | 0 |
| nodal efficiency | 2 | 1 |
| **high** vs **low disability** | **high > low disability** | **high < low disability** |
| nodal betweenness | 0 | 1 |
| nodal degree | 2 | 2 |
| nodal efficiency | 1 | 1 |
| **NEDA** vs **EDA** | **NEDA>EDA** | **NEDA<EDA** |
| nodal betweenness | 2 | 0 |
| nodal degree | 1 | 0 |
| nodal efficiency | 1 | 1 |

**Note:** Covariates were prespecified according to each comparison, as detailed in the main Methods. Abbreviations**:** MS: multiple sclerosis; HC: healthy controls; CI: Cognitively Impaired; CP: Cognitively Preserved; NEDA: no evidence of disease activity; EDA: evidence of disease activity; BMI: body mass index; TIV: total intracranial volume.

Supplementary Table S8. Regional topological abnormalities in the high- versus low-disability groups (EDSS ≥ 4 vs EDSS ≤ 3.5).

| **Regions** | **Metrics** | **Cohen’s *d*** | ***q value*** |
| --- | --- | --- | --- |
| **High > low disability** |  |  |  |
| rACC,Right Anterior cingulate & paracingulate gyri | nodal degree | 0.60 | 0.011 |
| lIOG,Left Inferior occipital gyrus | nodal degree | 0.56 | 0.013 |
| rPAL,Right Lenticular nucleus-Pallidum | nodal efficiency | 0.63 | 0.003 |
| **High < low disability** |  |  |  |
| lPHG,Left Parahippocampal gyrus | nodal betweenness | -0.60 | 0.011 |
| ltVL,Left Ventral lateral | nodal degree | -0.68 | <0.001 |
| rtVL,Right Ventral lateral | nodal degree | -0.52 | 0.010 |
| ltVPL,Left Ventral posterolateral | nodal efficiency | -0.49 | 0.016 |

Supplementary Table S9. Regional topological abnormalities in patients with cognitive impairment versus cognitively preserved patients (SDMT z ≤ −1.5 vs > −1.5).

| **Regions** | **Metrics** | **Cohen’s d** | ***q value*** |
| --- | --- | --- | --- |
| **CI > CP** |  |  |  |
| rACC, Right Anterior cingulate & paracingulate gyri | nodal degree | 0.60 | 0.037 |
| ltPuM, Left Pulvinar medial | nodal efficiency | 0.65 | 0.021 |
| rtAV, Right Thalamus-Anteroventral Nucleus | nodal efficiency | 0.64 | 0.020 |
| **CI < CP** |  |  |  |
| rSMA, Right Supplementary motor area | nodal efficiency | -0.70 | 0.019 |

**Abbreviations:** CI: Cognitively Impaired; CP: Cognitively Preserved.

Supplementary Table S10. Regional topological abnormalities in patients with evidence of disease activity versus patients meeting NEDA criteria.

| **Regions** | **Metrics** | **Cohen’s d** | ***q value*** |
| --- | --- | --- | --- |
| **EDA > NEDA** |  |  |  |
| lOLF, Left Olfactory cortex | nodal betweenness | 0.59 | 0.022 |
|  | nodal degree | 0.44 | 0.033 |
| rOLF, Right Olfactory cortex | nodal efficiency | 0.59 | 0.022 |
| rSMG, Right SupraMarginal gyrus | nodal betweenness | 0.62 | 0.023 |
| **EDA < NEDA** |  |  |  |
| lAMYG,Left Amygdala | nodal degree | -0.48 | 0.031 |

**Abbreviations:** NEDA: no evidence of disease activity; EDA: evidence of disease activity.

Supplementary Table S11: Longitudinal regional topological abnormalities between follow-up and baseline MRI in patients meeting NEDA criteria.

| **Regions** | **Metrics** | **Cohen’s d** | ***q value*** |
| --- | --- | --- | --- |
| **Follow-up MRI** < **Baseline** **MRI** |  |  |  |
| rANG,Right Angular gyrus | nodal degree | -0.65 | 0.045 |
| lPAL,Left Lenticular nucleus-Pallidum | nodal betweenness | -0.76 | 0.021 |
|  | nodal efficiency | -0.62 | 0.049 |
| rtPuL,Right Pulvinar lateral | nodal betweenness | -0.69 | 0.035 |

**Abbreviations:** NEDA: no evidence of disease activity.

Supplementary Table S12: **Demographic and Clinical Characteristics in EEG cohort of the Dataset 1**

| **Variables** | **HC(n = 80)** | **MS(n = 135)** | ***p* value** |
| --- | --- | --- | --- |
| Age at EEG, years, median (IQR) | 29.0 (25.0–44.2) | 34.0 (27.0–41.5) | 0.765^(a)^ |
| Sex, n (%) |  |  | 0.684^(b)^ |
| Female | 56 (70.00) | 98 (72.59) |  |
| Male | 24 (30.00) | 37 (27.41) |  |
| Left-/right-handed, n (%) |  |  | 0.474^(b)^ |
| right | 76 (95.00) | 131 (97.04) |  |
| left | 4 (5.00) | 4 (2.96) |  |
| BMI, kg/m², median (IQR) | 22.78 (20.58–24.70) | 22.45 (20.70–25.59) | 0.954^(a)^ |
| Disease duration, years, median (IQR) | — | 3.00(0.70–8.75) | — |
| EDSS at baseline, median (IQR) | — | 2.00 (0.00–2.50) | — |
| SDMT at baseline, median (IQR) | — | 50.00 (44.00–60.00) | — |
| Education years, median (IQR) | 16.0 (15.0–20.0) | 16.0 (12.0–16.0) | <0.001^(a)^ |

**Abbreviations:** BMI, body mass index; EDSS, Expanded Disability Status Scale; EEG, electroencephalography; HC, healthy controls; IQR, interquartile range; MS, multiple sclerosis; SDMT, Symbol Digit Modalities Test.
**Notes:** Data are presented as median (IQR) or n (%), as appropriate. Disease-specific variables are presented for the MS group only. *p* values were calculated for between-group comparisons of available variables. An em dash (—) indicates not applicable.

(a) *p* value derived from the Mann–Whitney U test.
(b) *p* value derived from Pearson’s chi-square test or Fisher’s exact test, as appropriate.

Supplementary Table S13: **Demographic and clinical characteristics of the hd-EEG cohort across prespecified subgroup analyses.**

| **Supplementary Table S13.1. High disability (EDSS ≥ 4.0) versus low disability (EDSS ≤ 3.5)** | | | |
| --- | --- | --- | --- |
| **Variables** | **EDSS ≤ 3.5 (n = 108)** | **EDSS ≥ 4.0 (n = 27)** | ***p* value** |
| Age at EEG, years, median (IQR) | 34.0 (27.0–40.2) | 36.0 (30.5–48.0) | 0.101^(a)^ |
| Sex, n (%) |  |  | 0.664^(b)^ |
| Female | 77 (71.30) | 21 (77.78) |  |
| Male | 31 (28.70) | 6 (22.22) |  |
| Left-/right-handed, n (%) |  |  | 0.583^(b)^ |
| right | 104 (96.30) | 27 (100.00) |  |
| left | 4 (3.70) | 0 (0.00) |  |
| BMI, kg/m², median (IQR) | 22.22 (20.41–25.20) | 23.16 (21.59–26.62) | 0.117^(a)^ |
| Disease duration, years, median (IQR) | 2.50 (0.50–7.00) | 9.00 (3.35–12.00) | <0.001^(a)^ |
| EDSS at baseline, median (IQR) | 1.50 (0.00–2.00) | 5.00 (4.00–6.25) | <0.001^(a)^ |
| SDMT at baseline, median (IQR) | 52.00 (45.00–61.00) | 44.00 (34.00–51.50) | <0.001^(a)^ |
| Education years, median (IQR) | 16.0 (12.0–16.0) | 15.0 (12.0–16.0) | 0.044^(a)^ |
| **Supplementary Table S13.2. Cognitive impairment (CI) versus cognitively preserved (CP)** | | | |
| **Variables** | **CP (n = 101)** | **CI (n = 34)** | ***p* value** |
| Age at EEG, years, median (IQR) | 32.0 (27.0–37.0) | 37.5 (32.2–50.0) | 0.003^(a)^ |
| Sex, n (%) |  |  | 0.599^(b)^ |
| Female | 75 (74.26) | 23 (67.65) |  |
| Male | 26 (25.74) | 11 (32.35) |  |
| Left-/right-handed, n (%) |  |  | 0.439^(b)^ |
| right | 99 (98.02) | 32 (94.12) |  |
| left | 2 (1.98) | 2 (5.88) |  |
| BMI, kg/m², median (IQR) | 22.58 (20.76–25.63) | 22.27 (20.16–24.39) | 0.617^(a)^ |
| Disease duration, years, median (IQR) | 2.40 (0.50–7.12) | 7.00(2.50–10.88) | 0.003^(a)^ |
| EDSS at baseline, median (IQR) | 1.50 (0.00–2.00) | 2.25 (2.00–5.25) | 0.023^(a)^ |
| SDMT at baseline, median (IQR) | 56.00 (50.00–64.75) | 34.50 (30.00–40.75) | < 0.001^(a)^ |
| Education years, median (IQR) | 16.0 (14.0–16.0) | 12.0 (12.0–15.0) | < 0.001^(a)^ |
| **Supplementary Table S13.3. Evidence of disease activity (EDA) versus no evidence of disease activity (NEDA)** | | | |
| **Variables** | **NEDA (n =78)** | **EDA (n = 57)** | ***p* value** |
| Age at EEG, years, median (IQR) | 34.0 (27.2–40.8) | 35.0 (26.0–43.0) | 0.672^(a)^ |
| Sex, n (%) |  |  | 0.157^(b)^ |
| Female | 53 (67.95) | 45 (78.95) |  |
| Male | 25 (32.05) | 12 (21.05) |  |
| Left-/right-handed, n (%) |  |  | 1.000^(b)^ |
| right | 76 (97.44) | 55 (96.49) |  |
| left | 2 (2.56) | 2 (3.51) |  |
| BMI, kg/m²,median (IQR) | 22.20 (20.45–25.65) | 23.44 (21.08–25.51) | 0.398^(a)^ |
| Disease duration, years, median (IQR) | 2.30 (0.20–7.00) | 6.00 (1.50–9.60) | 0.002^(a)^ |
| EDSS at baseline, median (IQR) | 1.50 (0.00–2.00) | 2.00 (1.00–3.50) | 0.010^(a)^ |
| SDMT at baseline, median (IQR) | 51.00 (45.00–61.50) | 50.00 (41.00–56.00) | 0.172^(a)^ |
| Education years, median (IQR) | 16.0 (12.0–16.0) | 16.0 (12.0–16.0) | 0.522^(a)^ |

**Abbreviations:** BMI, body mass index; CI, cognitively impaired; CP, cognitively preserved; EDA, evidence of disease activity; EDSS, Expanded Disability Status Scale; EEG, electroencephalography; IQR, interquartile range; NEDA, no evidence of disease activity; SDMT, Symbol Digit Modalities Test.
**Notes:** Data are presented as median (IQR) or n (%), as appropriate. *p* values refer to between-group comparisons within each prespecified subgroup analysis.

(a) *p* value derived from the Mann–Whitney U test.
(b) *p* value derived from Pearson’s chi-square test, continuity-corrected chi-square test, or Fisher’s exact test, as appropriate.

Supplementary Table S14. **List of the 104 AAL3 regions included in EEG source-space analyses.**

| **No.** | **AAL3 ID** | **Full region name** | **Hemisphere** | **Category** |
| --- | --- | --- | --- | --- |
| 1 | 45 | Left Amygdala | Left | Subcortical |
| 2 | 69 | Left Angular gyrus | Left | Cortical |
| 3 | 153 | Left Anterior cingulate cortex-pregenual | Left | Cortical |
| 4 | 151 | Left Anterior cingulate cortex-subgenual | Left | Cortical |
| 5 | 155 | Left Anterior cingulate cortex-supracallosal | Left | Cortical |
| 6 | 27 | Left Anterior orbital gyrus | Left | Cortical |
| 7 | 47 | Left Calcarine fissure and surrounding cortex | Left | Cortical |
| 8 | 75 | Left Caudate nucleus | Left | Subcortical |
| 9 | 95 | Left Crus I of cerebellar hemisphere | Left | Cerebellar |
| 10 | 49 | Left Cuneus | Left | Cortical |
| 11 | 59 | Left Fusiform gyrus | Left | Cortical |
| 12 | 23 | Left Gyrus rectus | Left | Cortical |
| 13 | 83 | Left Heschls gyrus | Left | Cortical |
| 14 | 41 | Left Hippocampus | Left | Subcortical |
| 15 | 11 | Left IFG pars orbitalis | Left | Cortical |
| 16 | 7 | Left Inferior frontal gyrus-opercular part | Left | Cortical |
| 17 | 9 | Left Inferior frontal gyrus-triangular part | Left | Cortical |
| 18 | 57 | Left Inferior occipital gyrus | Left | Cortical |
| 19 | 65 | Left Inferior parietal gyrus-excluding supramarginal and angular gyri | Left | Cortical |
| 20 | 93 | Left Inferior temporal gyrus | Left | Cortical |
| 21 | 33 | Left Insula | Left | Cortical |
| 22 | 31 | Left Lateral orbital gyrus | Left | Cortical |
| 23 | 51 | Left Lingual gyrus | Left | Cortical |
| 24 | 101 | Left Lobule IV-V of cerebellar hemisphere | Left | Cerebellar |
| 25 | 103 | Left Lobule VI of cerebellar hemisphere | Left | Cerebellar |
| 26 | 25 | Left Medial orbital gyrus | Left | Cortical |
| 27 | 37 | Left Middle cingulate & paracingulate gyri | Left | Cortical |
| 28 | 5 | Left Middle frontal gyrus | Left | Cortical |
| 29 | 55 | Left Middle occipital gyrus | Left | Cortical |
| 30 | 89 | Left Middle temporal gyrus | Left | Cortical |
| 31 | 17 | Left Olfactory cortex | Left | Cortical |
| 32 | 73 | Left Paracentral lobule | Left | Cortical |
| 33 | 43 | Left Parahippocampal gyrus | Left | Cortical |
| 34 | 61 | Left Postcentral gyrus | Left | Cortical |
| 35 | 39 | Left Posterior cingulate gyrus | Left | Cortical |
| 36 | 29 | Left Posterior orbital gyrus | Left | Cortical |
| 37 | 1 | Left Precentral gyrus | Left | Cortical |
| 38 | 71 | Left Precuneus | Left | Cortical |
| 39 | 145 | Left Pulvinar medial | Left | Subcortical |
| 40 | 165 | Left Red nucleus | Left | Subcortical |
| 41 | 13 | Left Rolandic operculum | Left | Cortical |
| 42 | 161 | Left Substantia nigra-pars compacta | Left | Subcortical |
| 43 | 3 | Left Superior frontal gyrus-dorsolateral | Left | Cortical |
| 44 | 19 | Left Superior frontal gyrus-medial | Left | Cortical |
| 45 | 21 | Left Superior frontal gyrus-medial orbital | Left | Cortical |
| 46 | 53 | Left Superior occipital gyrus | Left | Cortical |
| 47 | 63 | Left Superior parietal gyrus | Left | Cortical |
| 48 | 85 | Left Superior temporal gyrus | Left | Cortical |
| 49 | 15 | Left Supplementary motor area | Left | Cortical |
| 50 | 67 | Left SupraMarginal gyrus | Left | Cortical |
| 51 | 91 | Left Temporal pole: middle temporal gyrus | Left | Cortical |
| 52 | 87 | Left Temporal pole: superior temporal gyrus | Left | Cortical |
| 53 | 115 | Lobule IV-V of vermis | Midline/Other | Cerebellar |
| 54 | 46 | Right Amygdala | Right | Subcortical |
| 55 | 70 | Right Angular gyrus | Right | Cortical |
| 56 | 154 | Right Anterior cingulate cortex-pregenual | Right | Cortical |
| 57 | 152 | Right Anterior cingulate cortex-subgenual | Right | Cortical |
| 58 | 156 | Right Anterior cingulate cortex-supracallosal | Right | Cortical |
| 59 | 28 | Right Anterior orbital gyrus | Right | Cortical |
| 60 | 48 | Right Calcarine fissure and surrounding cortex | Right | Cortical |
| 61 | 96 | Right Crus I of cerebellar hemisphere | Right | Cerebellar |
| 62 | 50 | Right Cuneus | Right | Cortical |
| 63 | 60 | Right Fusiform gyrus | Right | Cortical |
| 64 | 24 | Right Gyrus rectus | Right | Cortical |
| 65 | 84 | Right Heschls gyrus | Right | Cortical |
| 66 | 42 | Right Hippocampus | Right | Subcortical |
| 67 | 12 | Right IFG pars orbitalis | Right | Cortical |
| 68 | 8 | Right Inferior frontal gyrus-opercular part | Right | Cortical |
| 69 | 10 | Right Inferior frontal gyrus-triangular part | Right | Cortical |
| 70 | 58 | Right Inferior occipital gyrus | Right | Cortical |
| 71 | 66 | Right Inferior parietal gyrus-excluding supramarginal and angular gyri | Right | Cortical |
| 72 | 94 | Right Inferior temporal gyrus | Right | Cortical |
| 73 | 34 | Right Insula | Right | Cortical |
| 74 | 32 | Right Lateral orbital gyrus | Right | Cortical |
| 75 | 52 | Right Lingual gyrus | Right | Cortical |
| 76 | 102 | Right Lobule IV-V of cerebellar hemisphere | Right | Cerebellar |
| 77 | 104 | Right Lobule VI of cerebellar hemisphere | Right | Cerebellar |
| 78 | 26 | Right Medial orbital gyrus | Right | Cortical |
| 79 | 136 | Right Mediodorsal medial magnocellular | Right | Subcortical |
| 80 | 38 | Right Middle cingulate & paracingulate gyri | Right | Cortical |
| 81 | 6 | Right Middle frontal gyrus | Right | Cortical |
| 82 | 56 | Right Middle occipital gyrus | Right | Cortical |
| 83 | 90 | Right Middle temporal gyrus | Right | Cortical |
| 84 | 18 | Right Olfactory cortex | Right | Cortical |
| 85 | 74 | Right Paracentral lobule | Right | Cortical |
| 86 | 44 | Right Parahippocampal gyrus | Right | Cortical |
| 87 | 62 | Right Postcentral gyrus | Right | Cortical |
| 88 | 40 | Right Posterior cingulate gyrus | Right | Cortical |
| 89 | 30 | Right Posterior orbital gyrus | Right | Cortical |
| 90 | 2 | Right Precentral gyrus | Right | Cortical |
| 91 | 72 | Right Precuneus | Right | Cortical |
| 92 | 14 | Right Rolandic operculum | Right | Cortical |
| 93 | 162 | Right Substantia nigra-pars compacta | Right | Subcortical |
| 94 | 164 | Right Substantia nigra-pars reticulata | Right | Subcortical |
| 95 | 4 | Right Superior frontal gyrus-dorsolateral | Right | Cortical |
| 96 | 20 | Right Superior frontal gyrus-medial | Right | Cortical |
| 97 | 22 | Right Superior frontal gyrus-medial orbital | Right | Cortical |
| 98 | 54 | Right Superior occipital gyrus | Right | Cortical |
| 99 | 64 | Right Superior parietal gyrus | Right | Cortical |
| 100 | 86 | Right Superior temporal gyrus | Right | Cortical |
| 101 | 16 | Right Supplementary motor area | Right | Cortical |
| 102 | 68 | Right SupraMarginal gyrus | Right | Cortical |
| 103 | 92 | Right Temporal pole: middle temporal gyrus | Right | Cortical |
| 104 | 88 | Right Temporal pole: superior temporal gyrus | Right | Cortical |

Supplementary Table S15. Regional topological abnormalities across hd-EEG frequency bands in patients with MS versus healthy controls.

| **Band** | **Direction** | **Regions** | **Metrics** | **Cohen’s d** | ***q* value** |
| --- | --- | --- | --- | --- | --- |
| low alpha | MS > HC | lOFClat, Left Lateral orbital gyrus | nodal degree | 0.42 | 0.003 |
| high alpha | MS > HC | rOFCpost, Right Posterior orbital gyrus | nodal degree | 0.47 | 0.001 |
| low beta | MS > HC | rSFG, Right Superior frontal gyrus-dorsolateral | nodal degree | 0.40 | 0.005 |
| low beta | MS < HC | rLING, Right Lingual gyrus | nodal betweenness | -0.43 | 0.002 |
| low beta | MS < HC | lMTG, Left Middle temporal gyrus | nodal betweenness | -0.43 | 0.002 |
| middle beta | MS < HC | rSFGmedial, Right Superior frontal gyrus-medial | nodal betweenness | -0.46 | 0.001 |
| delta | MS < HC | lPUT, Left Lenticular nucleus-Putamen | nodal efficiency | -0.41 | 0.004 |
| delta | MS < HC | rTHA, Right Thalamus | nodal efficiency | -0.43 | 0.002 |
| delta | MS < HC | rTPOsup, Right Temporal pole: superior temporal gyrus | nodal efficiency | -0.46 | 0.001 |

**Abbreviations:** MS: multiple sclerosis; HC: healthy control.

Supplementary Table S16. **Regional topological abnormalities of the hd-EEG band of high disability(EDSS≥4) versus low disability(EDSS≤3.5)**

| **Band** | **Direction** | **Regions** | **Metrics** | **Cohen’s d** | ***q* value** |
| --- | --- | --- | --- | --- | --- |
| middle beta | high disability < low disability | lIFGoperc, Left Inferior frontal gyrus-opercular part | nodal degree | -0.61 | 0.041 |
| middle beta | high disability < low disability | rMTG, Right Middle temporal gyrus | nodal efficiency | -0.64 | 0.025 |
| theta | high disability >low disability | rOFCpost, Right Posterior orbital gyrus | nodal betweenness | 0.62 | 0.036 |

**Abbreviations:** EDSS, Expanded Disability Status Scale; EEG, electroencephalography.

Supplementary Table S17. **Regional topological abnormalities of the hd-EEG band of EDA versus NEDA**

| **Band** | **Direction** | **Regions** | **Metrics** | **Cohen’s d** | ***q* value** |
| --- | --- | --- | --- | --- | --- |
| high alpha | EDA > NEDA | lINS, Left Insula | nodal efficiency | 0.55 | 0.025 |
| theta | EDA < NEDA | lAMYG, Left Amygdala | nodal efficiency | -0.60 | 0.015 |
| low beta | EDA < NEDA | rHIP, Right Hippocampus | nodal efficiency | -0.53 | 0.038 |
| delta | EDA < NEDA | rAMYG, Right Amygdala | nodal degree | -0.55 | 0.025 |
| delta | EDA < NEDA | lINS, Left Insula | nodal efficiency | -0.57 | 0.026 |

**Abbreviations:** EDA, evidence of disease activity; NEDA, no evidence of disease activity; hd-EEG, high-density electroencephalography.

Supplementary Table S18. **Regional topological abnormalities of the hd-EEG band of CI (SDMT Z ≤ -1.5 ) versus CP (SDMT Z > -1.5 )**

| **Band** | **Direction** | **Regions** | **Metrics** | **Cohen’s d** | ***q* value** |
| --- | --- | --- | --- | --- | --- |
| high beta | CI > CP | lSFGmedial, Left Superior frontal gyrus-medial | nodal betweenness | 0.74 | 0.022 |
| theta | CI > CP | lSFGmedial, Left Superior frontal gyrus-medial | nodal degree | 0.72 | 0.014 |
| theta | CI > CP | lMFG, Left Middle Frontal Gyrus | nodal efficiency | 0.70 | 0.016 |
| theta | CI < CP | lPHG, left Parahippocampal gyrus | nodal efficiency | -0.67 | 0.041 |
| delta | CI < CP | lMFG, Left Middle frontal gyrus | nodal degree | -0.73 | 0.012 |
| delta | CI < CP | rPHG, Right Parahippocampal gyrus | nodal efficiency | -0.70 | 0.026 |

**Abbreviations:** CI, cognitively impaired; CP, cognitively preserved; SDMT, Symbol Digit Modalities Test; hd-EEG, high-density electroencephalography.

Supplementary Table S19. **Performance of topology- and connectivity-based models across diagnostic and clinical stratification tasks**

| **Modality** | **Classification task**  **(n)** | **Feature set** | **Accuracy, %**  **(95% CI)** | **Sensitivity, %**  **(95% CI)** | **Specificity, %**  **(95% CI)** | **Balanced accuracy, %**  **(95% CI)** | **AUC (95% CI)** | **Permutation P for AUC** |
| --- | --- | --- | --- | --- | --- | --- | --- | --- |
| MRI (Dataset 1) | MS *vs* HC  (163 *vs* 248) | Topology | 84.8 (80.6–88.6) | 80.0 (72.9–85.2) | 88.0 (83.3–91.4) | 84.0 (80.3–87.7) | 0.845 (0.806–0.878) | 0.001 |
| MRI  (Dataset 1) | MS *vs* HC  (163 *vs* 248) | Connectivity | 89.5(86.2–92.1) | 86.0 (79.7–90.4) | 91.0 (86.9–94.1) | 88.5 (85.3–91.7) | 0.860 (0.786–0.884) | 0.001 |
| MRI (Dataset 1) | High vs low disability  (33 *vs* 130) | Topology | 82.1 (75.5–87.2) | 63.6 (46.6–77.8) | 86.8 (79.9–91.6) | 75.2 (66.6–84.0) | 0.731(0.699–0.891) | 0.010 |
| MRI  (Dataset 1) | High vs low disability  (33 *vs* 130) | Connectivity | 75.9 (68.8–81.9) | 66.7 (49.6–80.2) | 78.3 (70.7–84.5) | 72.5 (63.8–81.2) | 0.714 (0.519–0.741) | 0.016 |
| MRI  (Dataset 1) | CI *vs* CP  (44 *vs* 119) | Topology | 75.2 (66.8–80.6) | 74.4(67.5–80.2) | 75.0 (66.9–80.0) | 74.7 (67.2–80.1) | 0.750 (0.673–0.827) | 0.037 |
| MRI  (Dataset 1) | CI *vs* CP  (44 *vs* 119) | Connectivity | 71.8 (64.4–78.1) | 72.3 (63.6–79.5) | 70.5 (55.8–81.8) | 71.4 (59.7–80.7) | 0.713 (0.630–0.790) | 0.017 |
| MRI (Dataset 1) | EDA vs NEDA  (69 *vs* 94) | Topology | 70.8 (63.4–77.3) | 58.6 (46.9–69.4) | 80.2 (70.9–87.1) | 69.4 (58.9–78.3) | 0.756(0.729–0.871) | 0.009 |
| MRI (Dataset 1) | EDA *vs* NEDA  (69 *vs* 94) | Connectivity | 56.4 (47.5–62.6) | 55.0 (43.4–66.2) | 56.0 (46.3–66.0) | 55.5 (47.8–63.2) | 0.542 (0.460–0.640) | 0.497 |
| EEG (Dataset 1, theta) | MS *vs* HC  (135 *vs* 80) | Topology | 43.2 (36.8–49.9) | 45.0 (37.0–53.6) | 40.0 (30.0–51.0) | 42.5 (35.7–49.3) | 0.430 (0.360–0.520) | 0.902 |
| EEG (Dataset 1, theta) | MS *vs* HC  (135 *vs* 80) | Connectivity | 55.5 (43.0–61.3) | 55.0 (46.0–62.6) | 56.0 (40.0–60.0) | 55.4 (46.8–59.9) | 0.560 (0.410–0.620) | 0.468 |
| MRI (Dataset 2) | MS *vs* HC  (117 *vs* 107) | Topology | 65.2 (58.7–71.1) | 65.8 (56.8–73.8) | 64.5 (55.1–72.6) | 65.0 (60.3–74.7) | 0.684 (0.615–0.753) | 0.016 |
| MRI (Dataset 2) | MS *vs* HC  (117 *vs* 107) | Connectivity | 75.0 (68.9–80.2) | 75.2 (66.7–82.2) | 74.8 (65.8–80.2) | 74.5 (65.3–81.2) | 0.760 (0.698–0.822) | 0.001 |

**Note:** Data are presented as point estimates (95% confidence intervals). MRI-based classification tasks in Dataset 1 included MS vs HC (163 vs 248), high vs low disability (33 vs 130), CI vs CP (44 vs 119), and EDA vs NEDA (69 vs 94), and the MRI-based MS-vs-HC task was further examined in Dataset 2 (117 vs 107). EEG-based classification was performed for MS vs HC (135 vs 80), with theta-band results shown here. Separate linear support vector machine models were trained using topology-based and connectivity-based features for each task. Model performance was evaluated using accuracy, sensitivity, specificity, balanced accuracy, and area under the receiver operating characteristic curve (AUC), averaged across stratified 10-fold cross-validation. **Abbreviations:** AUC, area under the receiver operating characteristic curve; 95% CI, 95% confidence interval; CI, cognitively impaired; CP, cognitively preserved; EDA, evidence of disease activity; HC, healthy controls; MS, multiple sclerosis; NEDA, no evidence of disease activity.

Supplementary Table S20. Top 10 regions contributing to topology- and connectivity-based classification models for MS versus HC in Dataset 1.

| **Rank** | **Regions** | |
| --- | --- | --- |
|  | **Topology-based model** | **Connectivity-based model** |
| 1 | lPreCG, Left Precentral gyrus | rtMDm, Right Mediodorsal medial magnocellular |
| 2 | lSFG, Left Superior frontal gyrus-dorsolateral | ltLGN, Left Lateral geniculate |
| 3 | lAMYG, Left Amygdala | rtLGN, Right Lateral geniculate |
| 4 | lPAL, Left Lenticular nucleus-Pallidum | ltMGN, Left Medial Geniculate |
| 5 | rPAL, Right Lenticular nucleus-Pallidum | rtMGN, Right Medial Geniculate |
| 6 | rCER10, Right Lobule X of cerebellar hemisphere | ltPuA, Left Pulvinar anterior |
| 7 | ltMGN, Left Medial Geniculate | rtPuA, Right Pulvinar anterior |
| 8 | lRedN, Left Red nucleus | ltPuM, Left Pulvinar medial |
| 9 | rRedN, Right Red nucleus | rtPuM, Right Pulvinar medial |
| 10 | lIFGorb, Left IFG pars orbitalis | ltPuI, Left Pulvinar inferior |

**Abbreviations:** MS: multiple sclerosis; HC: healthy controls.

Supplementary Table S21. Top 10 regions contributing to topology- and connectivity-based classification models for MS versus HC in Dataset 2.

| **Rank** | **Regions** | |
| --- | --- | --- |
|  | **Topology-based model** | **Connectivity-based model** |
| 1 | ltPuM, Left Pulvinar medial | lSNpr, Left Substantia nigra-pars reticulata |
| 2 | ltAV, Left Ventral anterior | rtPuL, Right Pulvinar lateral |
| 3 | lPreCG,Left Precentral gyrus | rtIL, Right Intralaminar |
| 4 | rIOG, Right Inferior occipital gyrus | lCER10,Left Lobule X of cerebellar hemisphere |
| 5 | rtMDm, Right Mediodorsal medial magnocellular | rSOG, Right Superior occipital gyrus |
| 6 | lCAL, Left Calcarine fissure and surrounding cortex | ltPuA,Left Pulvinar anterior |
| 7 | rHES, Right Heschls gyrus | ltAV, Left Thalamus-Anteroventral Nucleus |
| 8 | lRedN,Left Red nucleus | rtPuA, Right Pulvinar anterior |
| 9 | VER9, Lobule IX of vermis | rtPuM,Right Pulvinar medial |
| 10 | rANG, Right Angular gyrus | ltPuI,Left Pulvinar inferior |

**Abbreviations:** MS: multiple sclerosis; HC: healthy controls.

Supplementary Table S22. Top 10 regions contributing to topology- and connectivity-based classification models for high versus low disability in Dataset 1.

| **Rank** | **Regions** | |
| --- | --- | --- |
|  | **Topology-based model** | **Connectivity-based model** |
| 1 | ltVL, Left Ventral lateral | lCAL, Left Calcarine fissure and surrounding cortex |
| 2 | rPCC, Right Posterior cingulate gyrus | rMOG, Right Middle occipital gyrus |
| 3 | rtVL, Right Ventral lateral | ltAV, Left Thalamus-Anteroventral Nucleus |
| 4 | lIOG, Left Inferior occipital gyrus | ltLP, Left Lateral posterior |
| 5 | rPAL, Right Lenticular nucleus-Pallidum | ltIL, Left Intralaminar |
| 6 | rtMDm, Right Mediodorsal medial magnocellular | rtIL, Right Intralaminar |
| 7 | ltLGN, Left Lateral geniculate | ltLGN, Left Lateral geniculate |
| 8 | lACCsup, Left Anterior cingulate cortex-supracallosal | ltPuA, Left Pulvinar anterior |
| 9 | rSNpc, Right Substantia nigra-pars compacta | rNacc, Right Nucleus accumbens |
| 10 | ltVA,Left Ventral anterior | lVTA, Left Ventral tegmental area |

Supplementary Table S23 .Top 10 regions contributing to topology- and connectivity-based classification models for CI versus CP in Dataset 1.

| **Rank** | **Regions** | |
| --- | --- | --- |
|  | **Topology-based model** | **Connectivity-based model** |
| 1 | rSMA, right Supplementary motor area | lPreCG, Left Precentral gyrus |
| 2 | lPCC, Left Posterior cingulate gyrus | rPreCG, Right Precentral gyrus |
| 3 | lCAL, Left Calcarine fissure and surrounding cortex | VER3, Lobule III of vermis |
| 4 | rSOG, Right Superior occipital gyrus | VER4_5, Lobule IV-V of vermis |
| 5 | lSTG, Left Superior temporal gyrus | ltIL, Left Intralaminar |
| 6 | rCERCRU2, Right Crus II of cerebellar hemisphere | rtIL, Right Intralaminar |
| 7 | rtLP, Right Lateral posterior | ltMDl, Left Mediodorsal lateral parvocellular |
| 8 | ltVPL, Left Ventral posterolateral | rtMDl, Right Mediodorsal lateral parvocellular |
| 9 | rtMDm, Right Mediodorsal medial magnocellular | rtLP, Right Lateral posterior |
| 10 | ltPuM, Left Pulvinar medial | lACCsup, Left Anterior cingulate cortex-supracallosal |

**Abbreviations:** CI, cognitively impaired; CP, cognitively preserved.

Supplementary Table S24. Top contributing regions for the topology-based classification model for EDA versus NEDA in Dataset 1.

| **Rank** | **Regions** | |
| --- | --- | --- |
|  | **Topology-based model** |  |
| 1 | rMOG, Right Middle occipital gyrus |  |
| 2 | lAMYG, Left Amygdala |  |
| 3 | lANG, Left Angular gyrus |  |
| 4 | rSTG, Right Superior temporal gyrus |  |
| 5 | rCERCRU1, Right Crus I of cerebellar hemisphere |  |
| 6 | VER1_2, Lobule I-II of vermis |  |
| 7 | VER3, Lobule III of vermis |  |
| 8 | lSNpr, Left substantia nigra pars reticulata |  |

**Abbreviations:** EDA, evidence of disease activity; NEDA, no evidence of disease activity.
**Note:** Given that the connectivity-based model showed near-chance discriminative performance, the regional contributions derived from this model were not further interpreted.

Supplementary Table S25. **Reproducibility tests for regional topological abnormalities using BN246 atlas.**

| **Regions** | **Metrics** | **Cohen's *d*** | ***q* value** |
| --- | --- | --- | --- |
| **MS > HC** | | | |
| L STG, medial area 38 | nodal betweenness | 0.541 | <0.0001 |
| R INS, dorsal dysgranular | nodal betweenness | 0.552 | <0.0001 |
| MVOcG, rostral lingual gyrus | nodal betweenness | 0.541 | <0.0001 |
| MVOcG, ventromedial parietooccipital sulcus | nodal betweenness | 0.531 | <0.0001 |
| MVOcC, ventromedial parietooccipital sulcus | nodal betweenness | 0.760 | <0.0001 |
| R IFG, ventral area 44 | nodal degree | 0.323 | 0.00186 |
| L OrG, area 13 | nodal degree | 0.323 | 0.00220 |
| R INS, dorsal agranular insular | nodal degree | 0.583 | <0.00001 |
| L INS, dorsal dysgranular insular | nodal degree | 0.626 | <0.00001 |
| R INS, dorsal dysgranular insular | nodal degree | 0.427 | <0.00001 |
| L MVOcG, rostral cuneus gyrus | nodal degree | 0.437 | <0.00001 |
| L LOcG, occipital polar cortex | nodal degree | 0.427 | <0.00001 |
| R LOcG, occipital polar cortex | nodal degree | 0.510 | <0.00001 |
| R LOcG, dorsal dysgranular insular | nodal degree | 0.566 | <0.00001 |
| L BG, ventromedial putamen | nodal degree | 0.885 | <0.00001 |
| R BG, ventromedial putamen | nodal degree | 0.457 | <0.00001 |
| R BG, dorsolateral putamen | nodal degree | 0.687 | <0.00001 |
| L Tha, medial pre-frontal thalamus | nodal degree | 0.833 | <0.00001 |
| R Tha, medial pre-frontal thalamus | nodal degree | 0.729 | <0.00001 |
| R Tha, sensory thalamus | nodal degree | 0.843 | <0.00001 |
| R Tha, rostral temporal thalamus | nodal degree | 0.510 | <0.00001 |
| R Tha, posterior parietal thalamus | nodal degree | 0.864 | <0.00001 |
| R Tha, occipital thalamus | nodal degree | 0.635 | <0.00001 |
| L Tha, occipital thalamus | nodal degree | 0.541 | <0.00001 |
| L ITG, intermediate lateral area 20 | nodal efficiency | 0.500 | <0.00001 |
| R ITG, intermediate lateral area 20 | nodal efficiency | 0.411 | <0.00001 |
| L INS, dorsal dysgranular insular | nodal efficiency | 0.455 | <0.00001 |
| L MVOcG, rostral cuneus gyrus | nodal efficiency | 0.489 | <0.00001 |
| L LOcC, occipital polar cortex | nodal efficiency | 0.482 | <0.00001 |
| R LOcC, occipital polar cortex | nodal efficiency | 0.482 | <0.00001 |
| L LOcC, medial superior occipital gyrus | nodal efficiency | 0.645 | <0.00001 |
| R BG, ventromedial putamen | nodal efficiency | 0.499 | <0.00001 |
| L BG, dorsolateral putamen | nodal efficiency | 0.869 | <0.00001 |
| R BG, dorsolateral putamen | nodal efficiency | 0.435 | <0.00001 |
| L Tha, medial pre-frontal thalamus | nodal efficiency | 0.794 | <0.00001 |
| R Tha, medial pre-frontal thalamus | nodal efficiency | 0.703 | <0.00001 |
| R Tha, pre-motor thalamus | nodal efficiency | 0.466 | <0.00001 |
| R Tha, sensory thalamus | nodal efficiency | 0.478 | <0.00001 |
| R Tha, rostral temporal thalamus | nodal efficiency | 0.509 | <0.00001 |
| R Tha, posterior parietal thalamus | nodal efficiency | 0.790 | <0.00001 |
| R Tha, occipital thalamus | nodal efficiency | 0.637 | <0.00001 |
| L Tha, caudal temporal thalamus | nodal efficiency | 0.818 | <0.00001 |
| **MS < HC** |  |  |  |
| L OrG, medial area 11 | nodal betweenness | -0.510 | <0.0001 |
| BG, nucleus accumbens | nodal betweenness | -0.708 | <0.0001 |
| dCa, dorsal caudate | nodal betweenness | -0.854 | <0.0001 |
| R Tha, rostral temporal thalamus | nodal betweenness | -0.635 | <0.0001 |
| L MFG, lateral area 10 | nodal degree | -0.323 | 0.00184 |
| L OrG, orbital area 12/47 | nodal degree | -0.367 | 0.00046 |
| L PrG, area 4 (upper limb region) | nodal degree | -0.854 | <0.00001 |
| R PrG, area 4 (upper limb region) | nodal degree | -0.729 | <0.00001 |
| R PrG, area 4 (trunk region) | nodal degree | -0.770 | <0.00001 |
| R FuG, ventrolateral area 37 | nodal degree | -0.549 | <0.00001 |
| L PhG, caudal area 35/36 | nodal degree | -0.770 | <0.00001 |
| R PhG, caudal area 35/36 | nodal degree | -0.500 | <0.00001 |
| L PhG, area 28/34 (EC, entorhinal cortex) | nodal degree | -0.744 | <0.00001 |
| R PhG, aarea 28/34 (EC, entorhinal cortex) | nodal degree | -0.718 | <0.00001 |
| L PhG, area TI(temporal agranular insular cortex) | nodal degree | -0.484 | <0.00001 |
| R PhG, area TI(temporal agranular insular cortex) | nodal degree | -0.573 | <0.00001 |
| L PoG, area 1/2/3 (upper limb, head and face region) | nodal degree | -0.687 | <0.00001 |
| R INS, ventral agranular insular | nodal degree | -0.426 | <0.00001 |
| L CG, dorsal area 23 | nodal degree | -0.562 | <0.00001 |
| R MVOcG, caudal lingual gyrus | nodal degree | -0.854 | <0.00001 |
| L LOcG, occipital polar cortex | nodal degree | -0.457 | <0.00001 |
| RAmyg, medial amyg | nodal degree | -0.433 | <0.00001 |
| RAmyg, lateral amyg | nodal degree | -0.435 | <0.00001 |
| L BG, dorsal caudate | nodal degree | -0.645 | <0.00001 |
| L PrG, area 4 (upper limb region) | nodal efficiency | -0.873 | <0.00001 |
| R PrG, area 4 (upper limb region) | nodal efficiency | -0.718 | <0.00001 |
| L PrG, area 4 (trunk region) | nodal efficiency | -0.439 | <0.00001 |
| R PrG, area 4 (trunk region) | nodal efficiency | -0.770 | <0.00001 |
| R ITG, extreme lateroventral area 37 | nodal efficiency | -0.519 | <0.00001 |
| R FuG, ventrolateral area 37 | nodal efficiency | -0.582 | <0.00001 |
| L PhG, caudal area 35/36 | nodal efficiency | -0.867 | <0.00001 |
| R PhG, caudal area 35/36 | nodal efficiency | -0.680 | <0.00001 |
| R PhG, aarea 28/34 (EC, entorhinal cortex) | nodal efficiency | -0.847 | <0.00001 |
| L PhG, area TI(temporal agranular insular cortex) | nodal efficiency | -0.509 | <0.00001 |
| R PhG, area TI(temporal agranular insular cortex) | nodal efficiency | -0.592 | <0.00001 |
| L PoG, area 1/2/3 (upper limb, head and face region) | nodal efficiency | -0.673 | <0.00001 |
| R INS, ventral agranular insular | nodal efficiency | -0.521 | <0.00001 |
| L CG, dorsal area 23 | nodal efficiency | -0.566 | <0.00001 |
| L MVOcG, caudal lingual gyrus | nodal efficiency | -0.459 | <0.00001 |
| R MVOcG, caudal lingual gyrus | nodal efficiency | -0.459 | <0.00001 |
| RAmyg, medial amyg | nodal efficiency | -0.664 | <0.00001 |
| RAmyg, lateral amyg | nodal efficiency | -0.487 | <0.00001 |
| L BG, globus pallidus | nodal efficiency | -0.589 | <0.00001 |
| R BG, globus pallidus | nodal efficiency | -0.541 | <0.00001 |
| L BG, dorsal caudate | nodal efficiency | -0.640 | <0.00001 |
| R BG, dorsal caudate | nodal efficiency | -0.731 | <0.00001 |
| **high > low disability** |  |  |  |
| L MFG, area 46 | nodal betweenness | 0.41 | 0.03 |
| L MFG, ventral area 9/46 | nodal betweenness | 0.40 | 0.04 |
| R MTG, rostral area 21 | nodal betweenness | 0.53 | 0.007 |
| R BG, Basal Ganglia | nodal betweenness | 0.39 | 0.04 |
| R RoG,medial area 14 | nodal degree | 0.55 | 0.006 |
|  | nodal efficiency | 0.54 | 0.0065 |
| R RoG, lateral area 11 | nodal degree | 0.43 | 0.03 |
|  | nodal efficiency | 0.41 | 0.03 |
| R ITG, rostral area 20 | nodal degree | 0.47 | 0.018 |
|  | nodal efficiency | 0.45 | 0.02 |
| L SPL, lateral area 5 | nodal degree | 0.39 | 0.047 |
|  | nodal efficiency | 0.41 | 0.036 |
| R INS, dorsal agranular insular | nodal efficiency | 0.40 | 0.04 |
| R INS, dorsal dysgranular insular | nodal efficiency | 0.40 | 0.04 |
| **high < low disability** |  |  |  |
| L SFG, dorsolateral area 6 | nodal betweenness | -0.40 | 0.04 |
| L PrG, caudal dorsolateral area 6 | nodal betweenness | -0.42 | 0.03 |
| L FuG, medioventral area37 | nodal betweenness | -0.48 | 0.0015 |
| R PhG,rostral area 35/36 | nodal betweenness | -0.53 | 0.007 |
| R Hipp, rostral hipp | nodal betweenness | -0.48 | 0.015 |
| R Tha, medial Pre-frontal thalamus | nodal betweenness | -0.52 | 0.009 |
| R IFG, dorsal area 44 | nodal degree | -0.70 | 0.0005 |
|  | nodal efficiency | -0.70 | 0.0004 |
| L FuG; medioventral area37 | nodal degree | -0.41 | 0.037 |
|  | nodal efficiency | -0.41 | 0.038 |
| L Psts: caudoposterior superior temporal sulcus | nodal degree | -0.49 | 0.001 |
|  | nodal efficiency | -0.39 | 0.04 |
| R IPL, caudal area 40(PFm) | nodal degree | -0.39 | 0.037 |
| R MTG, rostral area 21 | nodal efficiency | -0.51 | 0.0094 |
| L PrG, caudal ventrolateral area 6 | nodal efficiency | -0.51 | 0.0094 |
| R MTG, rostral area 21 | nodal efficiency | -0.43 | 0.028 |
| **CI > CP** |  |  |  |
| L BG, ventral caudate | nodal betweenness | 0.47 | 0.02 |
| L SFG,medial area 8 | nodal degree | 0.52 | 0.0087 |
| L INS, dorsal dysgranular insular | nodal efficiency | 0.61 | 0.0026 |
| **CI < CP** |  |  |  |
| R PCL,area1/2/3 (lower limb region) | nodal betweenness | -0.47 | 0.02 |
| R CG, dorsal area 23 | nodal betweenness | -0.47 | 0.02 |
| L INS, ventral agranular insular | nodal degree | -0.44 | 0.027 |
| R SFG,dorsolateral area 6 | nodal efficiency | -0.62 | 0.0023 |
| L MFG, dorsal area 9/46 | nodal efficiency | -0.53 | 0.0087 |
| R CG, caudal area 23 | nodal efficiency | -0.58 | 0.0043 |
| L Tha, sensory thalamus | nodal efficiency | -0.78 | 0.0001 |
| **EDA > NEDA** |  |  |  |
| R OrG, orbital area 12/47 | nodal betweenness | 0.55 | 0.00017 |
| R MTG,rostral area 21 | nodal efficiency | 0.62 | 0.0006 |
| R CG, dorsal area 23 | nodal degree | 0.6 | 0.0006 |
| **EDA < NEDA** |  |  |  |
| R MFG,dorsal area 9/46 | nodal betweenness | -0.49 | 0.00054 |
| R PhG,caudal area 35/36 | nodal betweenness | -0.46 | 0.0097 |

**Abbreviations**: BN246: Brainnetome Atlas with 246 subregions; MS; multiple sclerosis: MvOcC, Medioventral occipital cortex; CG, cingulate gyrus; STG, superior temporal gyrus; Tha:Thalamus; INS: Insular Gyrus; Amyg, Amygdala PhG, Parahippocampal gyrus; Hipp: Hippocampus; ITG: Inferior Temporal Gyrus; STG: superior temporal gyrus; FuG: Fusiform Gyrus; BG: Basal Ganglia; SFG: superior frontal gyrus; MFG: middle frontal gyrus; IPL, inferior parietal lobule; LOcC: Lateral Occipital Cortex; OrG: Orbital Gyrus; BG: Basal Ganglia; RoG: Orbital Gyrus; ITG:inferior temporal gyrus; Psts:posterior superior temporal sulcus; SPL: Superior parietal lobule; PrG: precentral Gyrus; MS: multiple sclerosis; HC: healthy control; CI: Cognitively Impaired; CP: Cognitively Preserved; NEDA: no evidence of disease activity; EDA: evidence of disease activity.

Supplementary Table S26. Density-range sensitivity analyses of MRI-derived iSCN global topological metrics in Dataset 1, adjusted for sex, age, BMI, and TIV

| Primary K = 0.10-0.40 global metrics | MS | HC | ***q value*** | direction |
| --- | --- | --- | --- | --- |
| AUC of γ | 0.694 ± 0.256 | 0.487± 0.38 | <0.001^*^ | MS > HC |
| AUC of λ | 0.363 ± 0.005 | 0.344 ± 0.006 | <0.001^*^ | MS > HC |
| AUC of σ | 0.566 ± 0.194 | 0.420 ± 0.342 | <0.001^*^ | MS > HC |
| AUC of Cp | 0.183 ± 0.005 | 0.200 ± 0.040 | <0.001^*^ | MS < HC |
| AUC of Lp | 0.593 ± 0.009 | 0.610 ± 0.011 | <0.001^*^ | MS < HC |
| AUC of Eg | 0.158 ± 0.018 | 0.153 ± 0.002 | <0.001^*^ | MS > HC |
| AUC of Eloc | 0.232 ± 0.003 | 0.239 ± 0.003 | <0.001^*^ | MS < HC |
| AUC of Q | 0.114 ± 0.006 | 0.086 ± 0.008 | <0.001^*^ | MS > HC |
| AUC of r | 0.080 ± 1.712 | 0.124 ± 0.065 | 0.070 | MS < HC |
| Sensitivity K = 0.20-0.50 global metrics | MS | HC | ***q value*** |  |
| AUC of γ | 0.537 ± 0.150 | 0.535 ±0.016 | 0.154 | MS > HC |
| AUC of λ | 0.327 ± 0.002 | 0.326 ± 0.002 | 0.5276 | MS > HC |
| AUC of σ | 0.489 ± 0.013 | 0.487 ± 0.013 | 0.202 | MS > HC |
| AUC of Cp | 0.194 ± 0.005 | 0.193 ± 0.006 | 0.2659 | MS > HC |
| AUC of Lp | 0.487 ± 0.004 | 0.487 ± 0.003 | 0.545 | MS ≈ HC |
| AUC of Eg | 0.187 ± 0.001 | 0.187 ± 0.001 | 0.335 | MS ≈ HC |
| AUC of Eloc | 0.241 ± 0.003 | 0.240 ± 0.004 | 0.062 | MS > HC |
| AUC of Q | 0.094 ± 0.007 | 0.091 ± 0.007 | 0.001* | MS > HC |
| AUC of r | 0.472 ± 0.815 | 0.401 ± 0.555 | 0.294 | MS > HC |
| Sensitivity K = 0.30-0.60 global metrics | MS | HC | ***q value*** |  |
| AUC of γ | 0.436 ± 0.012 | 0.435 ± 0.012 | 0.140 | MS > HC |
| AUC of λ | 0.313 ±0.002 | 0.312 ± 0.001 | 0.004* | MS > HC |
| AUC of σ | 0.48 ± 0.012 | 0.416 ±0.119 | 0.373 | MS > HC |
| AUC of Cp | 0.206 ± 0.005 | 0.205 ± 0.006 | 0.020* | MS > HC |
| AUC of Lp | 0.435 ± 0.126 | 0.434 ± 0.001 | 0.029* | MS > HC |
| AUC of Eg | 0.208 ± 0.001 | 0.208 ±0.000 | 0.0228* | MS > HC |
| AUC of Eloc | 0.249 ± 0.003 | 0.248 ± 0.003 | 0.003* | MS > HC |
| AUC of Q | 0.075 ± 0.006 | 0.072 ± 0.007 | < 0.001* | MS > HC |
| AUC of r | 0.328 ±0.104 | 0.351 ± 0.103 | 0.033* | MS < HC |

**Data were presented as mean±standard deviation**

***Statistical significance*:q:* FDR correction (adjusted P < 0.05);**

**Abbreviations:** MS: multiple sclerosis; HC: healthy control; iSCN: individual-level structural covariance network; AUC, area under the metric–density curve across the predefined network density thresholds; γ: normalised clustering coefficient; λ: normalised characteristic path length; σ: small-worldness; Cp: clustering coefficient; Lp: characteristic path length; Eg: global efficiency; Eloc: local efficiency; Q: modularity; r: assortativity; BMI: body mass index; TIV: total intracranial volume.

III Supplementary Figures

Supplementary Figure S1: Network small-worldness across the dynamic density thresholds. Each circle and error bar denote the mean and standard deviation of small-worldness at each density threshold, respectively. Abbreviations: MS, multiple sclerosis; HC, healthy controls.

Connectivity

Topology

theta ( MS vs HC)

AUC=0.430

AUC=0.560

Supplementary Figure S2. Classification performance of EEG-derived topology and connectivity features in the theta band. ROC curves for MS versus HC classification based on EEG-derived topological features (pink) and connectivity features (purple) in the theta band. The connectivity-based model showed better discriminative performance than the topology-based model (AUC = 0.560 vs 0.430). The x-axis represents the false positive rate and the y-axis represents the true positive rate. Theta was selected for display because it yielded the highest classification performance among the nine EEG frequency bands. Abbreviations: MS, multiple sclerosis; HC, healthy controls.

Supplementary Figure S3. Flowchart for selection of the longitudinal NEDA subgroup

Supplementary Figure S4A. Cortex, part 1

Supplementary Figure S4B. Cortex, part 2

Supplementary Figure S4C. Subcortical regions

**Supplementary Figure S4D. Cerebellum**

**Supplementary Figure S4. Significant AAL3-based nodal topological alterations in KL-based individualised structural covariance networks.**
(A, B) Cortex, split into two panels for readability. (C) Subcortical regions. (D) Cerebellum. Rows indicate brain regions, and columns indicate three nodal metrics: Bc, Dc, and Ne. Red bubbles indicate higher nodal values in patients with MS than in HC, whereas blue bubbles indicate lower nodal values in MS than in HC. Bubble size represents the absolute t value, capped at 10 for visualisation only; raw t values were unchanged. Only brain regions showing significant between-group differences are displayed. **Abbreviations**: MS, multiple sclerosis; HC, healthy controls; Bc, betweenness centrality; Dc, degree centrality; Ne, nodal efficiency.

 **Supplementary Figure S5. Reproducibility analyses of topological and connectivity abnormalities using the BN246 atlas.
(A)** Violin plots showing group differences in covariate-adjusted AUC values of global topological metrics derived from iSCNs reconstructed using BN246, including Cp, Lp, Eg, Eloc, σ, γ, λ, r and Q. ****: *q <* 0.0001. **(B)** Surface maps of significant nodal alterations in Bc, Dc, and Ne in the BN246-based analysis. Orange indicates MS > HC, and blue indicates MS < HC. **(C-I)** Edge-wise structural covariance connectivity matrix showing altered connections in the BN246-based analysis. **(C-II–C-III)** Functional-network-level distribution of increased (MS > HC) and decreased (MS < HC) connectivity, respectively. Darker colours and larger squares indicate greater relative connection weight, calculated as the ratio of the observed number of altered connections to the maximum possible number of connections. Asterisks inside the squares indicate the significance of functional network connections: *: *q* < 0.05, **: *q* < 0.01, ***: *q* < 0.001, ****: *q* < 0.0001. **Abbreviations**: AUC, area under the curve; BN246, Brainnetome atlas with 246 regions; CI, cognitively impaired; CP, cognitively preserved; DAN, dorsal attention network; DMN, default mode network; EDA, evidence of disease activity; FPN, frontoparietal network; HC, healthy controls; iSCN, individualised structural covariance network; LN, limbic network; MS, multiple sclerosis; NEDA, no evidence of disease activity; SMN, somatomotor network; VAN, ventral attention network; VN, visual network; Eg, global efficiency; Eloc, local efficiency;σ, small-worldness; γ, normalised clustering coefficient; λ, normalised characteristic path length; Q, modularity; r, assortativity; Cp, clustering coefficient; Dc, degree centrality; Ne, nodal efficiency; Bc, betweenness centrality; L, left; R, right.

**Supplementary Figure S6. Sensitivity analyses comparing KL- and JS-based individualised structural covariance networks (iSCNs) in multiple sclerosis. (A-I)** Violin plots showing group differences in covariate-adjusted AUC values of global topological metrics derived from KL-based iSCNs, including r, Eg, Eloc, σ, Lp, γ, λ, Cp, and Q. ***: *q* < 0.001, ns, not significant. **(A-II)** Corresponding group differences in global topological metrics derived from JS-based iSCNs. **(B-I–B-III)** Scatterplots showing concordance between KL-based and JS-based nodal t values for Bc, Dc, and Ne. Each dot represents one brain region. Red indicates regions significant in both analyses, green indicates regions significant only in the JS-based analysis, blue indicates regions significant only in the KL-based analysis, and grey indicates regions not significant in either analysis. Pearson correlation coefficients are shown in each panel. **(C-I–C-III)** Surface maps of significant nodal alterations identified using KL-based iSCNs for Bc, Dc, and Ne. **(D-I–D-III)** Surface maps of significant nodal alterations identified using JS-based iSCNs for Bc, Dc, and Ne. In panels C and D, orange indicates MS > HC, and blue indicates MS < HC. **(E)** KL-based connectivity alterations. **(E-I)** Chord diagram illustrating the overall pattern of altered functional-network connectivity. **(E-II–E-III)** Functional-network-level distribution of increased (MS > HC) and decreased (MS < HC) connectivity, respectively. **(F)** JS-based connectivity alterations. **(F-I)** Chord diagram illustrating the overall pattern of altered functional-network connectivity. **(F-II–F-III)** Functional-network-level distribution of increased (MS > HC) and decreased (MS < HC) connectivity, respectively. In panels E and F, darker colours and larger squares indicate greater relative connection weight, calculated as the observed number of altered edges divided by the maximum possible number of edges for each network pair. Asterisks denote FDR-corrected q values from the network-pair analysis: *: *q* < 0.05, **: *q* < 0.01, ***: *q* < 0.001, ****: *q* < 0.0001. **Abbreviations:** AUC, area under the curve; DMN, default mode network; Eg, global efficiency; Eloc, local efficiency;σ, small-worldness;γ,normalised clustering coefficient ; λ,normalised characteristic path length; Q, modularity, r, assortativity; Cp, clustering coefficient; Dc, degree centrality; Ne, nodal efficiency; Bc, betweenness centrality; Lp, characteristic path length; FPN, frontoparietal network; HC, healthy controls; iSCN, individualised structural covariance network; JS, Jensen–Shannon; KL, Kullback–Leibler; LN, limbic network; MS, multiple sclerosis; SMN, somatomotor network; VAN, ventral attention network; DAN, dorsal attention network; VN, visual network.

IV Supplementary Results

Supplementary Results S1: Reproducibility analyses using the BN246 atlas. Reproducibility analyses using the BN246 atlas broadly reproduced the main pattern observed in the primary AAL3-based analysis. At the global level, patients with MS showed lower Cp, Lp, Eloc, and Q, together with higher Eg, σ, γ, and λ, than healthy controls, and all eight metrics remained significant in the BN246-based analysis (Supplementary Fig. S5A). At the nodal level, significant abnormalities were again identified across betweenness centrality, degree centrality, and nodal efficiency. In the MS > HC direction, prominent alterations involved insular, occipito-temporal, basal ganglia, and thalamic regions, including the right dorsal dysgranular insula, medial and lateral occipital regions, ventromedial and dorsolateral putamen, and multiple thalamic subdivisions, such as the medial prefrontal, sensory, posterior parietal, and occipital thalamus. Additional increases were observed in the medial superior temporal region, ventral area 44 of the inferior frontal gyrus, and orbitofrontal cortex. In the MS < HC direction, significant abnormalities were retained in parietal and temporal association cortices, including the right inferior parietal lobule, right middle temporal gyrus, and left ventrolateral premotor/precentral region (Supplementary Fig. S5B; Supplementary Table S25). Overall, the BN246-based nodal findings continued to highlight cingulate–insular, thalamic, basal ganglia, and temporal–frontal involvement. At the connectivity level, the BN246-based edge-wise matrix again demonstrated both increased and decreased structural covariance components spanning frontal, temporal, parietal, occipital, and subcortical divisions (Supplementary Fig. S5C-I). Functional-division analysis further showed that abnormal connections remained concentrated in visual-network-related blocks, with both increased and decreased network-level alterations preserved in the BN246-based analysis (Supplementary Fig. S5C-II–C-III). Subgroup-related nodal abnormalities were also retained in the BN246-based analyses (Supplementary Fig. S5D; Supplementary Table S23). In the disability stratification, significant changes involved parietal, temporal, and prefrontal regions. In the cognitive stratification, CI was associated with abnormalities in the ventral caudate, medial superior frontal cortex, dorsal dysgranular insula, cingulate cortex, dorsolateral prefrontal cortex, and sensory thalamus. In the disease-activity stratification, EDA showed significant abnormalities in orbitofrontal, temporal, and cingulate regions, whereas decreases were observed in dorsolateral prefrontal and parahippocampal regions. Together, these findings indicate that the main topological and connectivity abnormalities were robust across atlas definitions.

Supplementary Results S2: **Sensitivity analyses using JS-based individualised structural covariance networks.** Sensitivity analyses using JS-based individualised structural covariance networks (iSCNs) showed an overall pattern that was broadly similar to that obtained with the primary KL-based approach, although the extent of abnormalities was reduced. At the global level, the KL-based analysis identified significant group differences in Eg, Eloc, Sigma, Lp, Gamma, Lambda, Cp, and Q, whereas ar was not significant. In the JS-based analysis, significant group differences were retained for Eloc, Sigma, Gamma, and Q, whereas ar, Eg, Lp, Lambda, and Cp were not significant (Supplementary Fig. S6A). At the nodal level, KL- and JS-based results showed significant positive concordance across all three nodal metrics. The nodal t values derived from the two approaches were correlated for betweenness centrality (Pearson r = 0.349, *p* < 0.001), degree centrality (r = 0.468, *p* < 0.001), and nodal efficiency (r = 0.321, *p* < 0.001) (Supplementary Fig. S6B). Surface maps further showed overlapping spatial distributions of significant nodal alterations between the two approaches, while the KL-based analysis identified a more extensive set of abnormal regions than the JS-based analysis (Supplementary Fig. S6C–D). At the connectivity level, both KL- and JS-based analyses identified altered structural covariance connectivity centred on the visual network. In both approaches, increased connections were mainly distributed within the visual network and in its couplings with other large-scale systems, whereas decreased connections were observed in a smaller set of visual-related network blocks. The functional-network matrices and chord diagrams showed that the JS-based analysis preserved the overall network distribution of the KL-based findings, but with fewer significant blocks and weaker relative connection weights (Supplementary Fig. S6E–F). Overall, the JS-based analyses yielded a spatially and topologically comparable, but less extensive, pattern of abnormalities relative to the KL-based results.

Supplementary Results S3: **Density-range sensitivity analysis**

Density-range sensitivity analyses using K = 0.20–0.50 and K = 0.30–0.60 showed partial, but not uniform, robustness to threshold selection. The modularity difference was consistently observed across the primary and additional density ranges, whereas several other global metrics, including small-worldness-related measures, varied across density ranges. Detailed results are provided in Supplementary Table S26.

## Supplementary Results S4**:**

## **hd-EEG graph-theoretical network alterations**

In the MS-versus-HC comparison, significant global topological differences were limited to a small number of frequency bands. MS showed higher global efficiency (Eg) in the theta band and higher modularity (Q) in the high alpha band; Small-worldness (σ) was also higher in the high alpha band. No other global topological metric survived correction. At the nodal level, eight significant regional abnormalities were identified after FDR correction (Supplementary Table S15). In the low alpha band, MS showed higher nodal degree in the left lateral orbital gyrus. In the high-alpha band, MS showed higher nodal degree in the right posterior orbital gyrus. In the low-beta band, MS showed higher nodal degree in the right dorsolateral superior frontal gyrus and lower nodal betweenness in the right lingual gyrus and left middle temporal gyrus. In the middle-beta band, MS showed lower nodal betweenness in the right medial superior frontal gyrus. In the delta band, MS showed lower nodal efficiency in the left putamen, right thalamus, and right superior temporal pole/superior temporal gyrus region. Overall, significant nodal alterations were distributed across frontal, temporal, and subcortical regions and involved frequency-specific changes in nodal degree, betweenness, and efficiency. In the disability stratification analysis, the high-disability group showed lower Eloc and higher Q in the high-alpha band, higher Q in the delta band, and lower Eg in the theta band. At the nodal level, three significant abnormalities were identified after FDR correction (Supplementary Table S16). In the middle-beta band, the high-disability group showed lower nodal degree in the left inferior frontal gyrus, opercular part, and lower nodal efficiency in the right middle temporal gyrus. In the theta band, the high-disability group showed higher nodal betweenness in the right posterior orbital gyrus.

In the cognitive stratification analysis, no significant global topological metric survived correction. Significant differences were confined to the nodal level, with six abnormalities retained after FDR correction (Supplementary Table S18). In the high-beta band, the CI group showed higher nodal betweenness in the left superior frontal gyrus, medial part. In the theta band, the CI group showed higher nodal degree in the left superior frontal gyrus, medial part, higher nodal efficiency in the left middle frontal gyrus, and lower nodal efficiency in the left parahippocampal gyrus. In the delta band, the CI group showed lower nodal degree in the left middle frontal gyrus and lower nodal efficiency in the right parahippocampal gyrus.

In the disease activity stratification analysis, no significant global topological difference was identified. Five significant nodal abnormalities were retained after FDR correction (Supplementary Table S17). In the high alpha band, the EDA group showed higher nodal efficiency in the left insula. In the theta band, the EDA group showed lower nodal efficiency in the left amygdala. In the low-beta band, the EDA group showed lower nodal efficiency in the right hippocampus. In the delta band, the EDA group showed lower nodal degree in the right amygdala and lower nodal efficiency in the left insula.

hd-EEG network connectivity abnormalities

In the MS-versus-HC comparison, significant connectivity abnormalities were identified in the low-alpha and low-gamma bands only, and both were in the MS > HC direction (Fig. 5B). In the low-alpha band, altered edges were relatively numerous and widely distributed, involving frontal connections with temporal, parietal, occipital, and subcortical regions, together with some subcortical-to-posterior cortical connections. In the low-gamma band, the number of altered edges was smaller and the spatial distribution was more restricted; the main pattern again involved frontal regions connecting with parietal, occipital, temporal, and subcortical regions. Functional-network mapping showed that, in the low-alpha band, significant network-pair level increases were confined to within-network VN and VAN. In the low-gamma band, the only significant increase was within-network DAN.

In the disability stratification analysis, connectivity abnormalities were detected primarily in the delta band, with the predominant direction being high disability > low disability (Fig. 5B). The altered edges were broadly distributed and involved multiple anatomical divisions. Network-pair analysis further showed that the delta-band abnormalities in the disability stratification were distributed across both within-network and between-network pairs (Fig. 5C).

In the cognitive stratification analysis, no significant connectivity abnormality was retained at the network level. Compared with the disability stratification, cognitive abnormalities in hd-EEG were therefore restricted primarily to nodal topological measures.

In the disease-activity stratification analysis, connectivity abnormalities were frequency-specific. Compared with the NEDA group, the EDA group showed increased connectivity in the low-beta band and decreased connectivity in the high-alpha band (Fig. 5B). Functional-network mapping showed that the disease-activity-related abnormalities were distributed across multiple network pairs (Fig. 5C), indicating that the significant connectivity changes were not confined to a single canonical network

Supplementary Results S5:

**EEG-based classification performance**
For EEG-based MS-versus-HC classification, models were trained separately for each frequency band. Among the nine EEG frequency bands, the theta band showed the highest classification performance and was therefore selected for visualisation in Supplementary Figure S2. In this band, the connectivity-based model outperformed the topology-based model, with AUC values of 0.560 and 0.430, respectively. Overall, EEG-based classification performance remained modest, but connectivity features showed relatively better discriminative ability than topological features in the best-performing band.

References

1.	Reich DS, Lucchinetti CF, Calabresi PA. Multiple Sclerosis. *N Engl J Med*. 2018;378(2):169-180.

2.	Schmidt P, Gaser C, Arsic M*, et al.* An automated tool for detection of FLAIR-hyperintense white-matter lesions in Multiple Sclerosis. *Neuroimage*. 2012;59(4):3774-3783.

3.	Gaser C, Dahnke R, Thompson PM*, et al.* CAT: a computational anatomy toolbox for the analysis of structural MRI data. *Gigascience*. 2024;13.

4.	Krüger J, Opfer R, Spies L*, et al.* Voxel-based morphometry in single subjects without a scanner-specific normal database using a convolutional neural network. *Eur Radiol*. 2024;34(6):3578-3587.

5.	Guo C, Ferreira D, Fink K*, et al.* Repeatability and reproducibility of FreeSurfer, FSL-SIENAX and SPM brain volumetric measurements and the effect of lesion filling in multiple sclerosis. *Eur Radiol*. 2019;29(3):1355-1364.

6.	Wang P, Cai H, Luo R*, et al.* Measurement of Cortical Atrophy and Its Correlation to Memory Impairment in Patients With Asymptomatic Carotid Artery Stenosis Based on VBM-DARTEL. *Front Aging Neurosci*. 2021;13:620763.

7.	Rolls ET, Huang CC, Lin CP*, et al.* Automated anatomical labelling atlas 3. *Neuroimage*. 2020;206:116189.

8.	Lai H, Kong X, Zhao Y*, et al.* Patterns of a structural covariance network associated with dispositional optimism during late adolescence. *Neuroimage*. 2022;251:119009.

9.	Wang H, Jin X, Zhang Y*, et al.* Single-subject morphological brain networks: connectivity mapping, topological characterization and test-retest reliability. *Brain Behav*. 2016;6(4):e00448.

10.	Li Y, Wang N, Wang H*, et al.* Surface-based single-subject morphological brain networks: Effects of morphological index, brain parcellation and similarity measure, sample size-varying stability and test-retest reliability. *Neuroimage*. 2021;235:118018.

11.	Watts DJ, Strogatz SH. Collective dynamics of 'small-world' networks. *Nature*. 1998;393(6684):440-442.

12.	Bullmore E, Sporns O. Complex brain networks: graph theoretical analysis of structural and functional systems. *Nat Rev Neurosci*. 2009;10(3):186-198.

13.	Matsuo K, Harada K, Fujita Y*, et al.* Distinctive Neuroanatomical Substrates for Depression in Bipolar Disorder versus Major Depressive Disorder. *Cereb Cortex*. 2019;29(1):202-214.

14.	Orrù G, Pettersson-Yeo W, Marquand AF*, et al.* Using Support Vector Machine to identify imaging biomarkers of neurological and psychiatric disease: a critical review. *Neurosci Biobehav Rev*. 2012;36(4):1140-1152.
